# Revision of the Atratus Group of *Culex* (*Melanoconion*) (Diptera: Culicidae)

**DOI:** 10.1186/s13071-020-3982-x

**Published:** 2020-05-27

**Authors:** Ivy Luizi Rodrigues de Sá, Rosa Sá Gomes Hutchings, Roger William Hutchings, Maria Anice Mureb Sallum

**Affiliations:** 1 Departamento de Epidemiologia, Faculdade de Saúde Pública, Universidade de São Paulo, Av. Doutor Arnaldo 715, São Paulo, SP 01246-904 Brazil; 2 Laboratório de Bionomia e Sistemática de Culicidae, Coordenação de Biodiversidade, Instituto Nacional de Pesquisas da Amazônia, Av. André Araújo 2.936, Manaus, AM 69067-375 Brazil

**Keywords:** Taxonomy, Revision, Identification keys, Morphology, Illustrations, Distribution, New species, Culicini

## Abstract

**Background:**

Despite the importance of some species of *Culex* (*Melanoconion*) (Diptera: Culicidae) as vectors of several arboviruses that cause diseases in humans and other animals, there are few taxonomic studies focusing on species of the subgenus, especially providing morphological keys for species identification.

**Results:**

Thirteen species of the Atratus Group of *Culex* (*Melanoconion*) were reviewed, five new species are described, and two taxonomic changes are proposed: *Cx*. (*Mel*.) *exedrus* Root, 1927 and *Cx*. (*Mel*.) *loturus* Dyar, 1925 are resurrected from synonymy with *Cx*. (*Mel*.) *dunni* Dyar, 1918 and *Cx*. (*Mel*.) *zeteki* Dyar, 1918, respectively. The Atratus Group now includes fourteen species: *Cx*. (*Mel*.) *atratus* Theobald, 1901; *Cx*. (*Mel*.) *caribeanus* Galindo & Blanton, 1954; *Cx.* (*Mel.*) *columnaris* Sá & Hutchings n. sp.; *Cx.* (*Mel.*) *commevynensis* Bonne-Wepster & Bonne, 1919; *Cx*. (*Mel*.) *comptus* Sá & Sallum n. sp.; *Cx*. (*Mel*.) *dunni*; *Cx*. (*Mel*.) *ensiformis* Bonne-Wepster & Bonne, 1919; *Cx*. (*Mel*.) *exedrus*; *Cx*. (*Mel*.) *longisetosus* Sá & Sallum n. sp.; *Cx*. (*Mel*.) *longistylus* Sá & Sallum n. sp.; *Cx*. (*Mel*.) *loturus*; *Cx*. (*Mel*.) *spinifer* Sá & Sallum n. sp.; *Cx*. (*Mel*.) *trigeminatus* Clastrier, 1970; and *Cx*. (*Mel*.) *zeteki*. Keys, descriptions and illustrations for the identification of the male, female, pupal and fourth-instar larval stages of each species are provided. The treatment of each species includes a complete synonymy, descriptions of available life stages, a taxonomic discussion, updated bionomics and geographical distribution, and a list of material examined.

**Conclusions:**

The taxonomy of the Atratus Group of *Culex* (*Melanoconion*) is updated, including descriptions of five new species. The number of valid species is greater than the number recognized in the previous taxonomic study of the group, increasing from seven to 14 species. Distributional and bionomical data are updated. Morphology-based identification keys for females, males, fourth-instar larvae and pupae provided in this study will facilitate species identification.

## Background

Species of the subgenus *Melanoconion* Theobald, 1903 of *Culex* Linnaeus, 1758 are considered to be of public health importance because they are vectors of several arboviruses, such as the West Nile virus, viruses of the Venezuelan equine encephalitis complex, and eastern equine encephalomyelitis virus [1–5]. Despite their medical importance, there are few taxonomic studies that focus on species of this subgenus, especially providing key characters for species identification [6–9]. Although some morphological characters of the fourth-instar larvae and pupae can be useful for species identification, there are few studies that focus primarily on identification of the immature stages. The most complete studies are those by Foote [7] and Sirivanakarn [8]. Thus, for accurate species identification, it is necessary to examine features of the dissected male genitalia, with the structures viewed in dorsal, lateral and ventral aspects [9, 10].

The subgenus *Melanoconion* includes 160 valid species and 79 synonyms for several species from both the Spissipes and the Melanoconion Sections [8, 10–12]. The current internal classification of the subgenus *Melanoconion* was proposed by Sirivanakarn [8], with some alterations proposed by Sallum & Forattini [9] based on morphological similarities shared by species.

The Spissipes Section comprises 23 species separated into eight groups and three subgroups [9]. The Melanoconion Section consists of 137 species separated into 13 groups and 20 subgroups [8]. Recently, Torres-Gutierrez et al. [13] investigated the phylogenetic relationships among species of the Spissipes and Melanoconion Sections using DNA sequences of the mitochondrial gene cytochrome *c* oxidase subunit 1 (*cox*1) and two nuclear genes: hunchback (*hb*) and carbamoyl-phosphate synthetase 2, aspartate transcarbamylase and dihydroorotase (*CAD*) of 43 species. The authors demonstrated the monophyly of the Spissipes and Melanoconion Sections, and that most of the morphology-based groups of the Spissipes Section are also monophyletic, corroborating the morphological classification previously proposed by Sirivanakarn [8]. In contrast, some incongruence was found in the internal classification of the Melanoconion Section in comparison with the placement of species into monophyletic lineages recovered in the molecular analyses. The monophyly of the Atratus and Pilosus Groups were corroborated in all analyses. However, it is important to note that the taxon sampling employed in the molecular phylogenetic analyses was limited, with underrepresented groups/subgroups.

The Atratus Group includes seven valid species [8] and five synonyms [10]: *Cx*. *atratus* Theobald, 1901 (syns *Cx*. *advieri* Senevet, 1938; *Cx*. *falsificator* Dyar & Knab, 1909); *Cx*. *caribeanus* Galindo & Blanton, 1954; *Cx*. *commevynensis* Bonne-Wepster & Bonne, 1919; *Cx*. *dunni* Dyar (syns *Cx*. *exedrus* Root, 1927; *Cx*. *ruffinis* Dyar & Shannon, 1924); *Cx*. *ensiformis* Bonne-Wepster & Bonne, 1919; *Cx*. *trigeminatus* Clastrier, 1970; and *Cx*. *zeteki* Dyar, 1918 (syn. *Cx*. *loturus* Dyar, 1925). The geographical distribution of the Atratus Group ranges from southern South America to northern Central America with *Cx*. *atratus* dispersed on some Caribbean islands, and *Cx*. *dunni* as the only member of the group recorded in Mexico [14–16].

*Culex dunni* has epidemiological importance as a potential vector of arboviruses that can infect and cause encephalitis in humans, as it has been found naturally infected with Pacora (PCA) virus [17]. In addition, *Cx. dunni* has been reported to be vector of Venezuelan equine encephalitis (VEE) virus in Panama [18].

Several taxonomic changes have been made related to the species of the Atratus Group before the classification proposed by Sirivanakarn [8]. Dyar [19] placed *Culex atratus*, *Cx*. *zeteki*, *Cx*. *dunni*, *Cx*. *commevynensis*, *Cx*. *ruffinis* and *Cx*. *loturus* in the Melanoconion Section. Edwards [20], based on adult characteristics, divided the subgenus into Groups A, B and C, and placed *Cx*. *commevynensis* in Group B, and *Cx*. *atratus*, *Cx*. *zeteki*, *Cx*. *dunni* (syn. *Cx*. *ensiformis*), *Cx*. *ruffinis* (syn. *Cx*. *exedrus*) and *Cx*. *loturus* in Group C. Komp [21] considered *Cx*. *ruffinis* as a synonym of *Cx*. *dunni* and *Cx*. *loturus* as a synonym of *Cx*. *zeteci*. Rozeboom & Komp [6] corrected the spelling of *Cx*. *zeteci* to *Cx*. *zeteki*, in accordance with provisions of the International Code of Zoological Nomenclature. Galindo & Blanton [22] described *Cx*. *caribeanus* and Clastrier [23] described *Cx*. *trigeminatus*, both based on unique features of the male genitalia. Sirivanakarn [8] classified the species of the subgenus into groups, creating the Atratus Group.

Accurate species identification is necessary for studies focusing on biology, ecology, vectorial capacity and vector competence. This study aimed to review the taxonomy of the Atratus Group and update the data on species bionomics and distributions. Additionally, five new species are formally named and described, two species are elevated from synonymy and illustrated identification keys to the species level are provided for females, males, fourth-instar larvae and pupae.

## Methods

The specimens examined during this study are from the Coleção Entomológica de Referência, Faculdade de Saúde Pública, Universidade de São Paulo (FSP-USP), São Paulo, Brazil and from the Coleção de Invertebrados, Instituto Nacional de Pesquisas da Amazônia (INPA), Manaus, Brazil. All specimens come from field collections made in several localities in the Brazilian states of Acre, Amazonas, Mato Grosso do Sul, Minas Gerais, Pará, Rondônia and São Paulo. Type specimens of the nominal species, deposited in the Diptera Collection in the National Museum of Natural History (USNM), Washington, D.C., USA and in the Natural History Museum (NHM), London, UK, were also examined, except for the types of *Cx*. *commevynensis* Bonne-Wepter & Bonne, 1919 and *Cx*. *trigeminatus* Clastrier, 1970. Female and male genitalia along with immature specimens from the same locality and habitat were examined when available. When available, male genitalia, larval and pupal exuviae associated to the pinned adult were mounted on the same slide. Character measurements, of 2–5 specimens when available, were obtained in the same manner as Sallum & Hutchings [24]. Illustrations of male genitalia structures were produced using a Leitz Wetzlar Diaplan microscope with a Leitz Wetzlar drawing tube. All measurements are in millimeters and are given as the range followed by the mean and the number of measurements in parentheses. The descriptions follow the morphological terminology in Harbach & Knight [25, 26], with some modifications made by Harbach et al. [27]. Only the morphological characters that are unusual and diagnostic for each species are detailed. The *Culex* classification adopted is that proposed by Harbach [11]. The Anophelinae classification adopted is that proposed by Foster et al. [28].

Geographical distributions are based on both literatute records and material examined, including field collections and museum specimens examined. Distribution records of the material examined are listed in the following format: country, state, municipality and/or locality name, latitude and longitude.

*Culex commevynensis* is not included in this revision because the type specimen could not be examined.

To comply with the regulations set out in Article 8.5 of the amended 2012 version of the *International Code of Zoological Nomenclature* (ICZN) [29], details of all new taxa have been submitted to ZooBank. The Life Science Identifier (LSID) of the article is urn:lsid:zoobank.org:pub:837DA7C4-9E36-4E25-9F4E-17241FA79DAD. For each new taxon, the Life Science Identifier (LSID) is reported in the taxonomic summary.

The abbreviations used are: L, larva; Le, larval exuviae; P, pupa; Pe, pupal exuviae; ♂, male; ♀, female; ♂G, male genitalia; Syn., synonym; distr., distribution; tax., taxonomy; info., information; desig., designation; emend., emendation; FSP-USP, Faculdade de Saúde Pública of Universidade de São Paulo, São Paulo, Brazil; INPA, Instituto Nacional de Pesquisas da Amazônia, Manaus, Amazonas, Brazil; NHM, Natural History Museum, London, UK; USNM, National Museum of Natural History, Washington DC, USA; MNHN, Museum National dʼHistoire Naturelle, Paris, France.

## Results

**Atratus Group**

According to Sirivanakarn [8], the following combination of morphological features diagnose the adults of the Atratus Group: head with narrow decumbent scales on central area of vertex and patch of broad decumbent scales laterally; pleural integument of thorax with pale and dark stripes across mesokatepisternum and mesepimeron; patch of numerous pale scales on upper corner of mesokatepisternum. Gonocoxite of male genitalia small, narrow, oblong; gonostylus narrow, simple, without a subapical crest; basal hook of lateral plate of aedeagus sclerotized, slender, in form of a curved arm. The morphological characteristics of the pupae are as follows: seta 9-VIII inserted before caudolateral angle of segment; seta 11-C usually single; trumpet long with index 10 or greater. Larvae can be recognized by the following combination of characteristics: margin of saddle with small spicules; seta 2-C absent; seta 14-C inserted at same level as 15-C or slightly anterior; siphon slender, long, with 4–6 pairs of posterolateral elements and with 3 or 4 pairs of dorsolateral elements.

**Taxonomic treatment**

***Culex*** (***Melanoconion***) ***atratus*Theobald, 1901**

1901: *Culex atratus* Theobald, 1901: 55 [30] (♂, ♀) lectotype ♂, paralectotype ♀ deposited in the NHM; topotypes ♂, ♀ deposited in the USNM. Type locality: Ferry Swamp, Jamaica.
1909: *Culex falsificator* Dyar & Knab, 1909: 258 [31] (♂) lectotype ♂ (USNM). Type locality: La Havana, Cuba.
1938: *Culex advieri* Senevet, 1938: 185 [32] (♂, ♂G) holotype ♂ (MNHN). Type locality: Prise d’Eau, Guadeloupe, Lesser Antilles.

*Melanoconion atratus* of Theobald (1903: 238) [33] (L, P, distr.); Dyar (1905: 49) [34] (type species desig. for *Melanoconion*).

*Culex atratus* of Howard et al. (1915: 388) [35] (♀, ♂, ♂G, L, P).

*Culex* (*Melanoconion*) *atratus* of Dyar (1923: 187) [36] (♂G); Bonne & Wepster-Bonne (1925: 268) [37] (♂, ♂G, L); Rozeboom & Komp (1950: 87) [6] (♂G); Foote (1954: 21) [7] (L, P); Belkin et al. (1965: 32) [38] (type info., distr.); Belkin (1968: 13) [39] (type info.); Belkin et al. (1970: 78) [40] (♂, ♀, L, P, distr.); Pecor et al. (1992: 12) [15] (distr., type info.); Kobayashi (1999: 9) [41] (tax.); Torres-Gutierrez & Sallum (2015: 12) [10] (type info., distr.).

*Culex falsificator* of Pazos (1909: 50) [42] (distr.); Pazos (1914: 17) [43] (tax.); Howard et al. (1912: 425) [44] (♀, ♂, ♂G).

*Culex* (*Melanoconion*) *falsificator* of Edwards (1932: 214) [20] (synonymy with *Cx*. *atratus*); Stone & Knight (1957: 49) [45] (desig. lectotype); Belkin et al. (1965: 15) [38].

*Culex advieri* of Rozeboom & Komp (1950: 87) [6] (synonymy with *Cx*. *atratus*)

*Culex* (*Melanoconion*) *advieri* of Floch & Abonnenc (1945: 29) [46] (♂, ♂G, L); Belkin et al. (1965: 27) [38] (type info., bionomics); Belkin (1968: 12) [39].

***Type material*:** Lectotype, pinned adult male (NHM 010630134) in poor condition associated with male genitalia, and paralectotype pinned adult female (NHM 0106300135) in good condition in the Diptera Collection, Natural History Museum (NHM), London, UK; topotypic male and female in the Diptera Collection, National Museum of Natural History (USNM), Washington, DC, USA. Lectotype male and paralectotype female of *Cx*. *atratus* examined as photographs provided by the Natural History Museum, London, UK, for comparisons.

***Material examined*:** 36 specimens: 25 ♂G, 5 Le, 3 Pe, 7♂. USNM, Jamaica: JA798-90 (♂); JA 798-30 (G♂, ♂); JA798-32 (♀); JA6- 101 (♂G); JA25-102 (♂G); JA2/3-84 (♂G); JA871-102 (♂G); JA744-90 (♂G, ♂); JA798-96 (♂G, ♂); JA414-103 (♂G); JA701-67/208-2 (♂G); JA727-102 (♂G, ♂); JA719-6/208-3 (♂G); JA871-103 (♂G); JA899-10 (♂G); JA701-67/213-15 (♂G); JA719-67/213-16 (♂G); JA759-91 (♂G); JA798-40 (♂G); JA744-95 (♂G); JA862-67/213-17 (♂G); JA862-67/208-4 (♂G); JA744-1 (Le); JA798-3 (Le); JA899 (Le); JA862-2 (Le). Dominican Republic: RD1 (Le, Pe); RD2 (Pe); RD3 (Pe). French West Indies: FWI201-14 (♂G); FWI198-101 (♂G). Haiti: HAT11-103 (♂G); HAT8-680923-8 (♂G). Synonym *Culex falsificator*: Lectotype, pinned adult male (USNM no. 12108), with associated dissected genitalia (USNM no. 408) in good condition in the Diptera Collection, National Museum of Natural History (USNM), Washington, DC, USA.

***Distribution*:***Culex atratus* has been found in Cuba [15]; Jamaica [3]; Dominica [47]; Dominican Republic, French West Indies, Guadeloupe, Haiti, Puerto Rico, Surinam, Trinidad, Virgin Islands [15]; Brazil [48–50]; Cayman Islands [51]; Florida Keys, USA [52]; Venezuela [53] and Panama [54]. The occurrence of the species in Brazil needs to be verified with new collections in the localities where it has been recorded.

**Description**

**Fig. 1:**
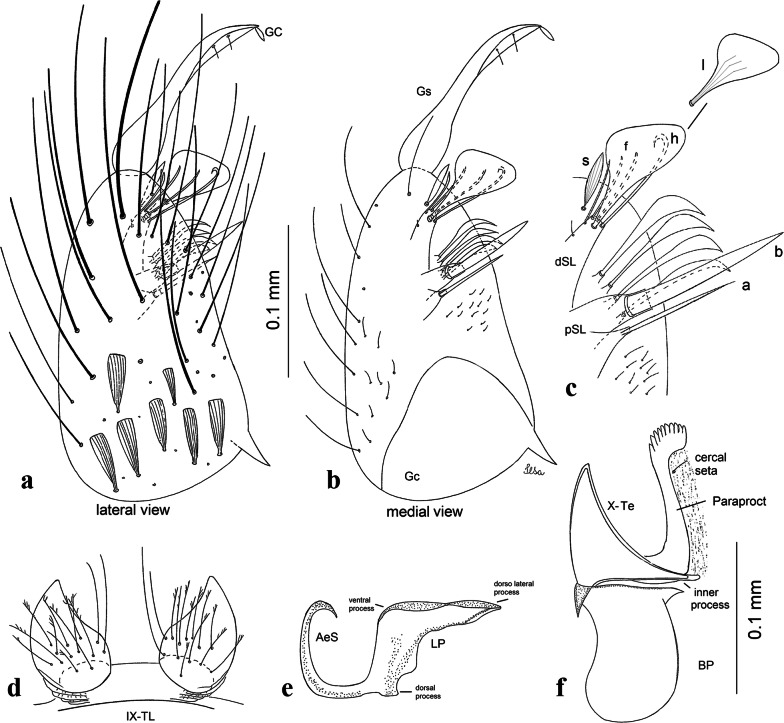
*Culex* (*Melanoconion*) *atratus*, male genitalia. **a** Gonocoxite in lateral view. **b** Gonocoxite in medial view. **c** Setae on the subapical lobe of the gonocoxite. **d** Tergum IX lobe. **e** Aedeagus. **f** Proctiger. *Abbreviations*: Gc, gonocoxite; Gs, gonostylus; GC, gonostylar claw; dSL, distal division of subapical lobe; pSL, proximal division of subapical lobe; IX-TL, tergum IX lobe; AeS, aedeagal sclerite; LP, lateral plate; BP, basal plate; X-Te, tergum X

**Fig. 2:**
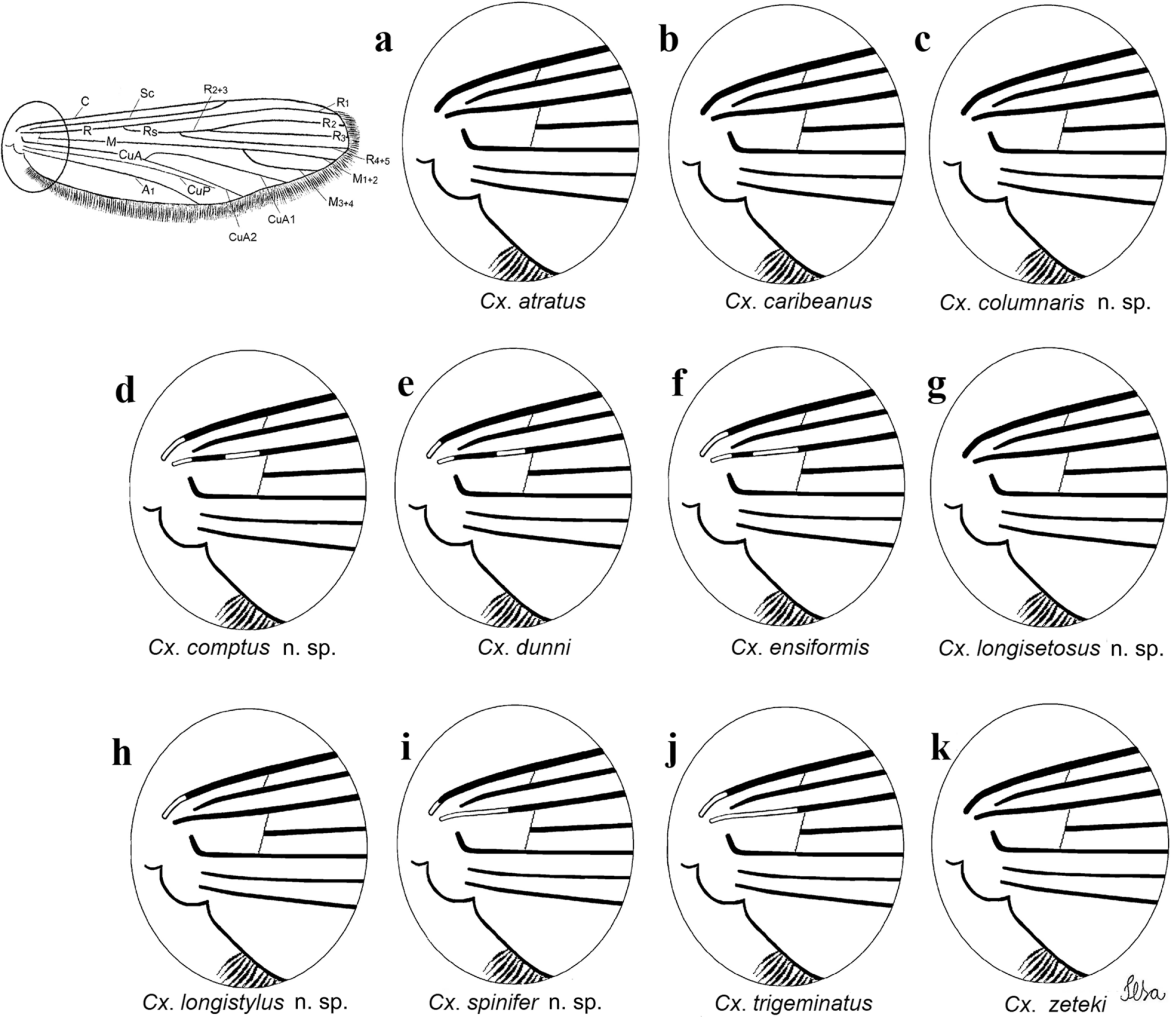
Comparison of proximal spots on the wings of the species of the Atratus Group, in dorsal view. **a***Culex atratus*. **b***Culex caribeanus*. **c***Culex columnaris* n. sp. **d***Culex comptus* n. sp. **e***Culex dunni*. **f***Culex ensiformis*. **g***Culex longisetosus* n. sp. **h***Culex longistylus* n. sp. **i***Culex spinifer* n. sp. **j***Culex trigeminatus*. **k***Culex zeteki*. *Abbreviations*: C, costa; SC, subcostal vein; R, radius; RS, radial sector; R_2+3_, radius 2+3; R_1_, radius 1; R_2_, radius 2; R_3_, radius 3; R_4+5_, radius 4+5; M, media; M_1+2_, media 1+2; M_3+4_, media 3+4; CuA, cubitus anterior; CuP, cubitus posterior; A_1_, Anal vein; CuA_1_, cubitus anterior 1; CuA_2_, cubitus anterior 2

***Male*.** [Figs. 1, 2a] Small body, scutum covered with dark scales with reddish-brown reflections, pale scales on thoracic pleura. *Head*: antenna dark, verticillate; length 1.17‒1.25 (1.20) (*n* = 3); proboscis entirely dark-scaled, length 1.56–1.74 (1.67) (*n* = 3); maxillary palpus dark-scaled, length 2.08–2.21 (2.13) (*n* = 3); palpomere III with few long, strong setae at apex and inconspicuous basal white ring; palpomeres IV-V entirely covered with long, strong setae; clypeus and antennal pedicel dark. Vertex with narrow, white decumbent falcate scales on central area and erect, dark forked scales; large lateral patch of broad, decumbent white scales extending dorsally; ocular line with narrow, white falcate scales extending dorsally; occiput with dark erect forked scales. *Thorax*: integument brown; scutum covered with narrow, dark brown falcate scales with lightly golden-coppery reflections. Scutal setae large, dark brown with golden-coppery reflections. Scutellar scales similar to prescutellar scales on median and lateral lobes; median lobe with 4–6 large, dark setae; lateral lobes each with 3 or 4 setae. Pleural integument with pattern of pale and dark brown areas as follows: dark brown on postspiracular area, upper mesokatepisternum, upper and lower mesepimeron; pale on lower mesokatepisternum, mesomeron. Pleural setae with 2 types of colouring: setae dark brown with bronzy reflections: 7 or 8 antepronotal, 4–6 prealar; and pleural setae pale golden, hyaline: 4 or 5 upper mesokatepisternal, 3–5 lower mesokatepisternal, 3 or 4 upper mesepimeral and 1 large lower mesepimeral. Pleura with patch of broad, white scales on upper mesokatepisternum; lower mesokatepisternum with few scales not forming patch. *Wing*: dark-scaled, length 2.26–2.31 (2.29) (*n* = 3). Dorsal scales broad, dark distally on veins R_1_, R_2_, R_3_, R_4+5_, M_1+2_ and M_3+4;_ appressed, dark spatulate scales on veins C, Sc, R, proximally on M, Cu_1_, Cu_2_ and A_1_; linear scales on R_s_, R_2+3_; remigium with appressed spatulate scales and 2 setae. *Halter*: scabellum yellowish; pedicel yellowish, narrow, with brown dorsal strip; capitellum brown, with few scales with golden reflections. *Legs*: coxae pale; ventral surface of fore- and midfemur with longitudinal stripe of white scales; tibiae dark-scaled; joints of fermur-tibia and tibia-tarsomere I with ring of pale scales; tarsi entirely dark-scaled. *Abdomen*: tergum I with dark scales, terga III-VIII dark-scaled with basolateral patches of white scales; sterna II-VII with broad basal white bands. *Genitalia*: ninth tergal lobes pear-shaped, each with 12–14 slender, aciculate setae inserted at 0.67 from base, apex glabrous; distance between lobes 0.6 of width of one lobe at base. Gonocoxite small, narrow, oblong; subapical lobe divided into 2 columnar divisions; proximal division with 2 parallel, apically pointed setae (*a* and *b*); seta *a* shorter, slender, inserted basal to seta *b*; seta *b* spatulate and stronger than seta *a*. Distal division with short columnar process, with 5 setae: 3 filiform, narrow, pointed, apically inserted, subequal in size (setae *f*), 1 long seta, with hook-like apex (seta *h*), 1 large, broad, asymmetrical, ribbed seta arising subapically (seta *l*); 1 saber-like, ribbed seta (seta *s*) arising apically. Gonocoxite with 4 or 5 broad, hyaline, flattened, apically curved setae borne ventromesally between proximal and distal divisions. Gonostylus slender, slightly curved, tapering towards apex; apex moderately blunt, ventral surface with 2 apical hyaline setae; one short leaf-like gonostylar claw arising apically. Aedeagal sclerite and lateral plate equal in length; lateral process of lateral plate sclerotized, slightly pointed, directed dorsolaterally; ventral process almost straight. Aedeagal sclerite curved in lateral view. Proctiger with tergum X somewhat triangular, slender in outline, inner process pointed. Basal plate with concave inner margin. Paraproct elongate, crown with 9 or 10 simple blades. Cercal sclerite with 1 or 2 setae.

***Female*.** Not examined.

**Fig. 3:**
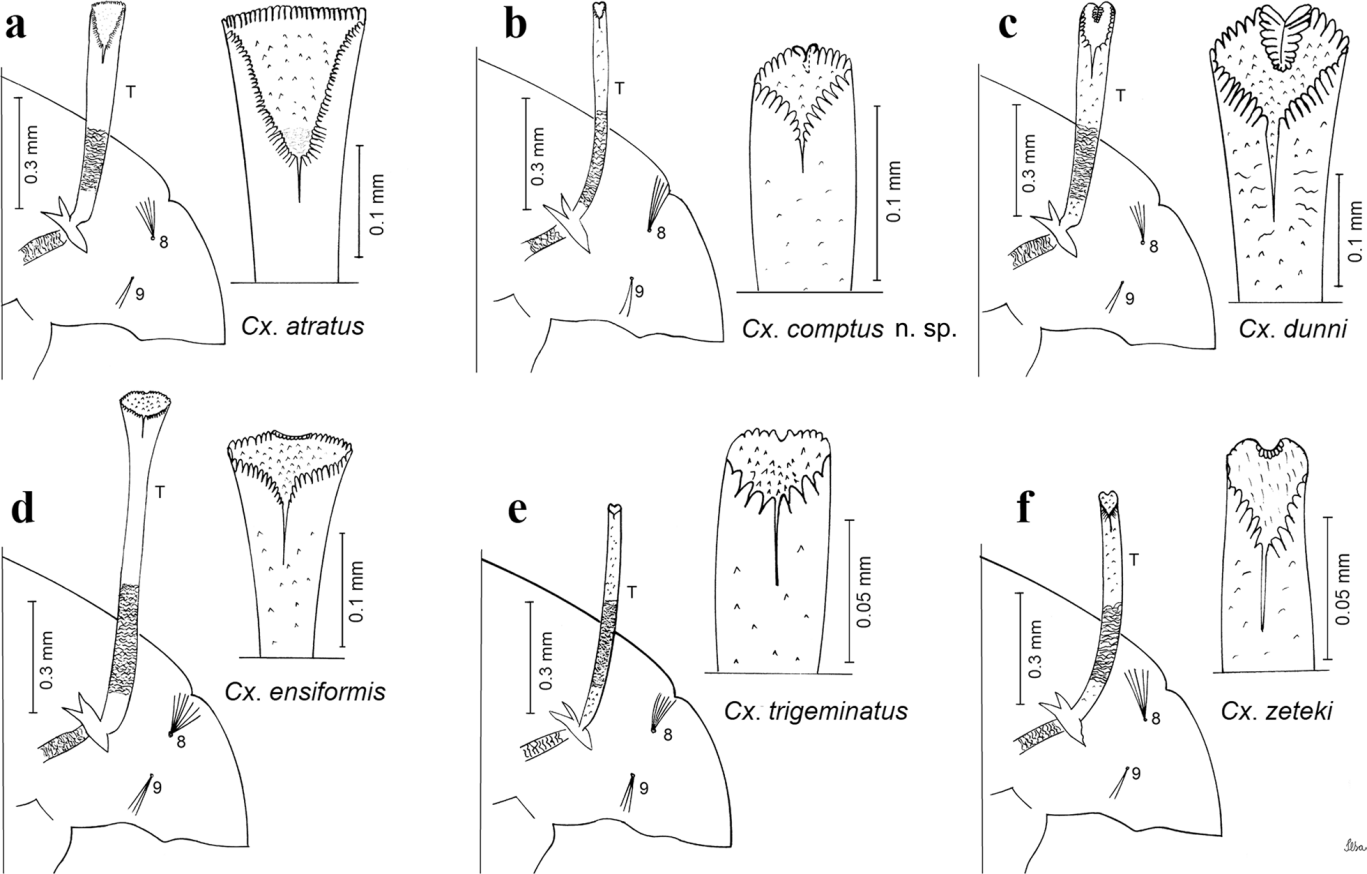
Comparison of trumpets in pupae of species of the Atratus Group. **a***Culex atratus*. **b***Culex comptus* n. sp. **c***Culex dunni*. **d***Culex ensiformis*. **e***Culex trigeminatus*. **f***Culex zeteki*. *Abbreviation*: T, trumpet

**Fig. 4:**
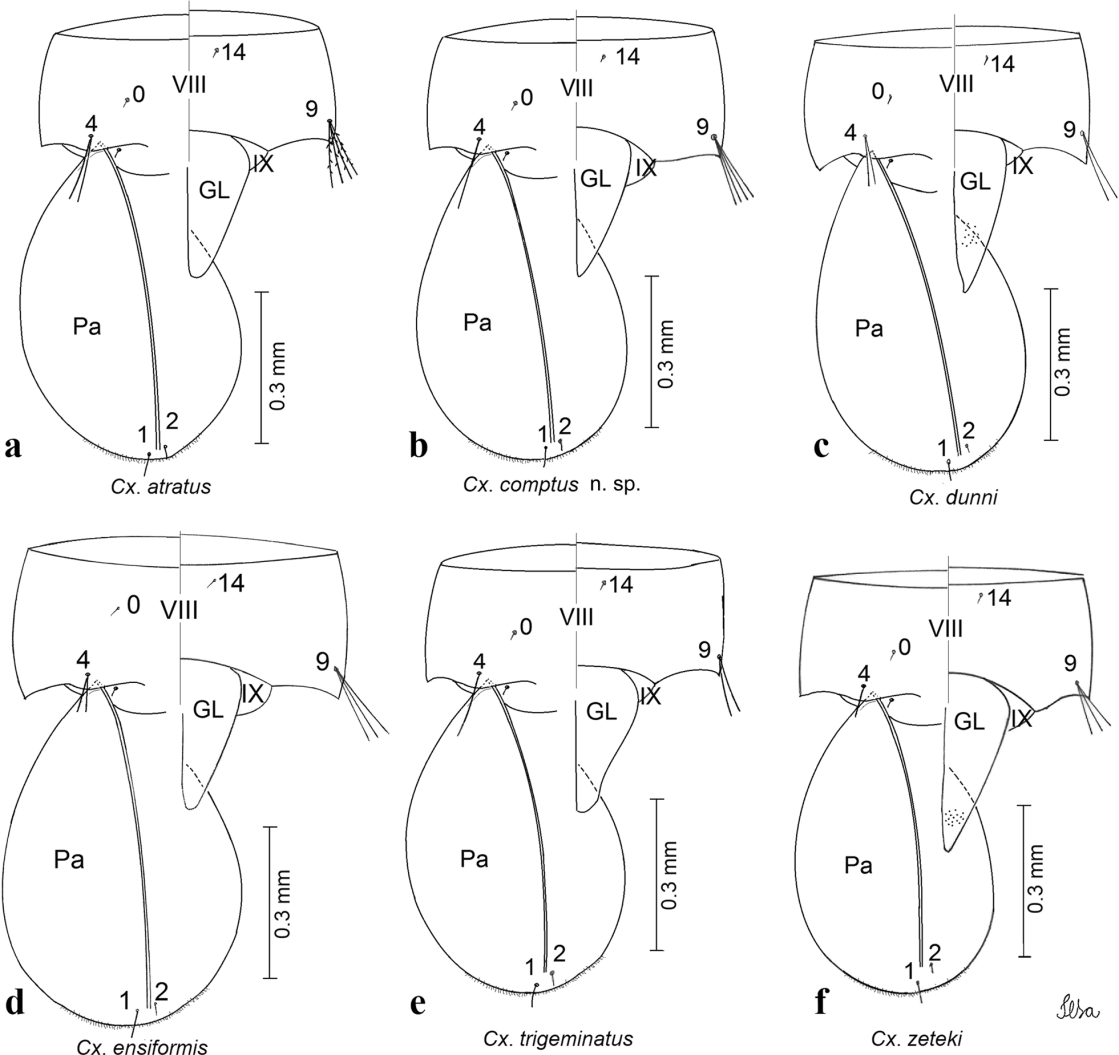
Comparison of pupal segment VIII in species of the Atratus Group. **a***Culex atratus*. **b***Culex comptus* n. sp. **c***Culex dunni*. **d***Culex ensiformis*. **e***Culex trigeminatus*. **f***Culex zeteki*. *Abbreviations*: Pa, paleta; VIII, segment VIII; GL, genital lobe

***Pupa.*** [Figs. 3a, 4a] Integument lightly tanned. *Cephalothorax*: setae 1,2-CT 4-branched (*n* = 2); setae 3,4-CT 2-branched; seta 5-CT 4-branched; seta 6-CT 2-branched; seta 7-CT 3-branched; seta 8-CT 5- or 6-branched; seta 9-CT 2- or 3-branched; seta 10-CT 5- or 6-branched; seta 11-CT single; seta 12-CT 2- or 3-branched. Trumpet moderately tanned. Pinna small, V-shaped in lateral view; tracheoid area, darker, extending almost 0.45 from base; trumpet index *c.*7. *Abdomen*: lightly tanned; seta 9-VIII with 4 aciculate branches. Paddle weakly tanned; setae 1,2-Pa single, 1-Pa longer than 2-Pa.

**Fig. 5:**
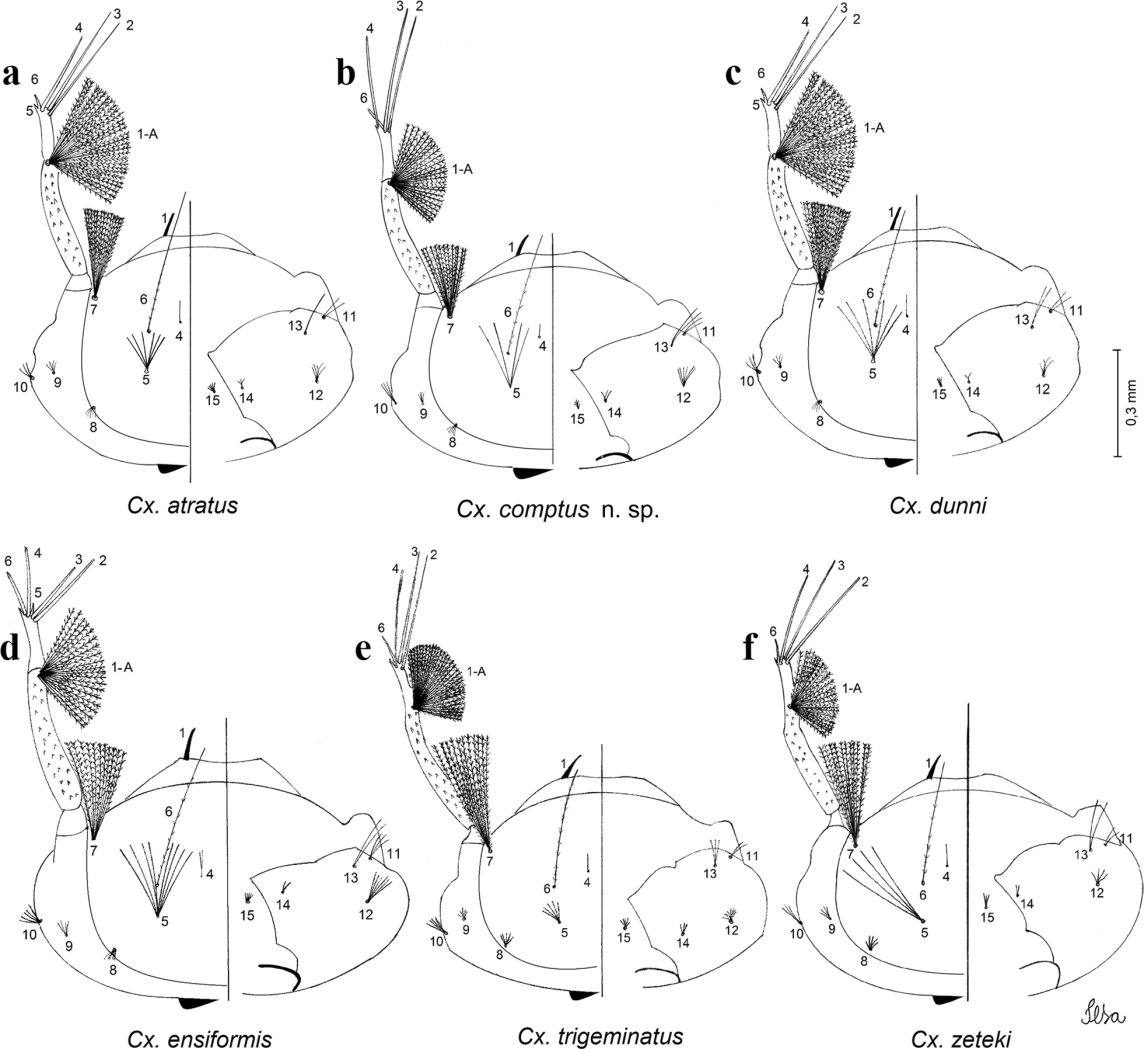
Comparison of heads in fourth-instar larvae of species of the Atratus Group. **a***Culex atratus*. **b***Culex comptus* n. sp. **c***Culex dunni*. **d***Culex ensiformis*. **e***Culex trigeminatus*. **f***Culex zeteki*

**Fig. 6:**
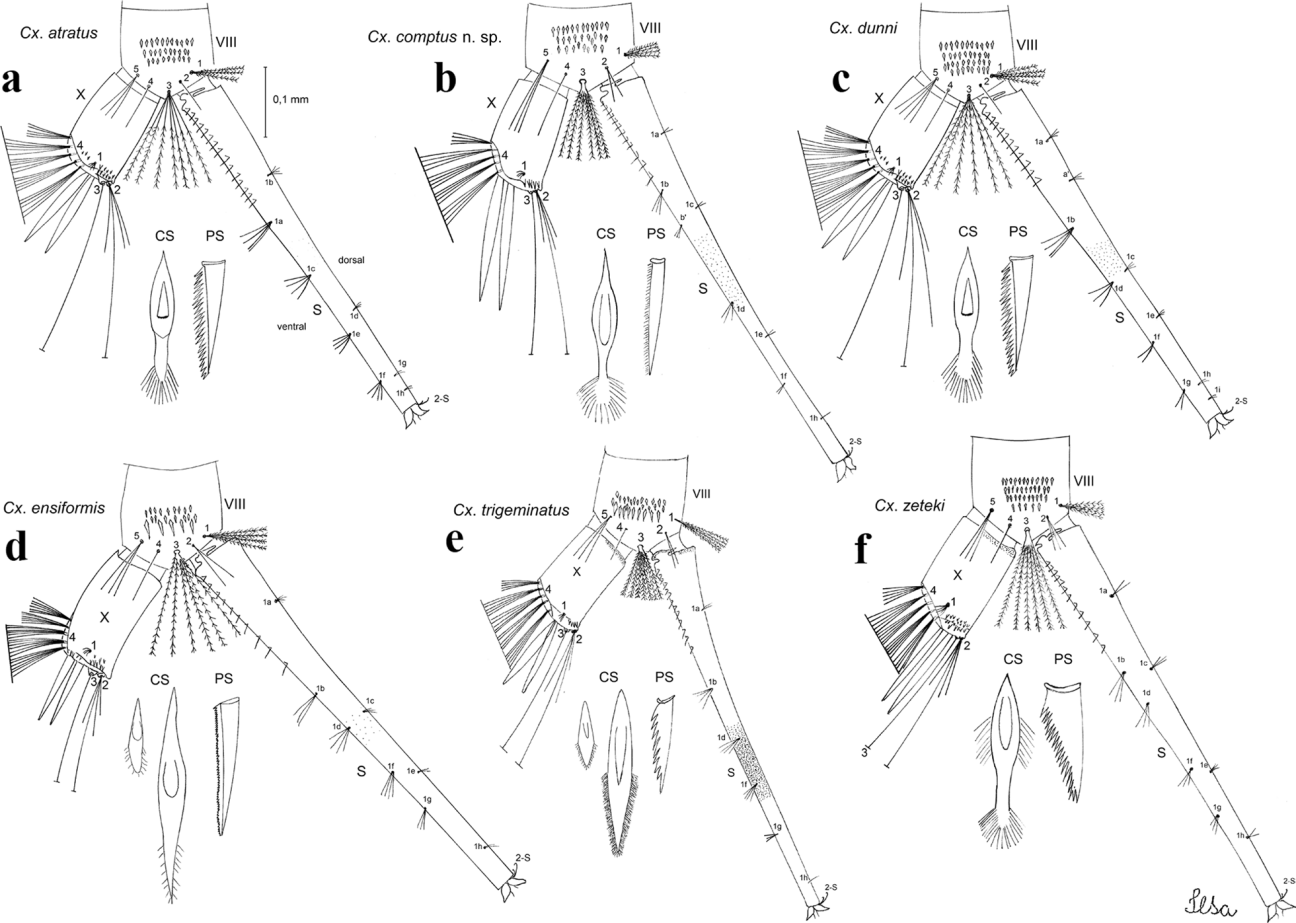
Comparison of segment VIII and siphon in fourth-instar larva of species of the Atratus Group. **a***Culex atratus*. **b***Culex comptus* n. sp. **c***Culex dunni*. **d***Culex ensiformis*. **e***Culex trigeminatus*. **f***Culex zeteki*. *Abbreviations*: S, siphon; VIII, segment VIII; X, segment X; CS, comb scale, in detail; PS, pecten spine, in detail

***Larva*.** [Figs. 5a, 6a] *Head*: wider than long; capsule moderately tanned; lateralia and collar darker; length and width not measured; dorsomentum with 1 large median tooth and 4 small teeth on either side. Antenna lightly tanned with dark rings at base and level of seta 1-A; setae 2,3-C absent (*n* = 2); seta 4-C single; seta 5-C with 6 long branches reaching 6-C insertion; seta 6-C single, long, reaching anterior margin of head, with sparse minute spicules on basal 0.5; seta 7-C with 12 aciculate branches; seta 8-C 5- or 6 branched; seta 9-C 4-branched; seta 10-C 3-branched; seta 11-C double; seta 12-C 4- or 5-branched; seta 13-C single; seta 14-C 3-branched; seta 15-C with multiple hyaline branches. *Thorax*: integument hyaline; pleura without darker patches under integument. *Abdomen*: integument hyaline; comb of segment VIII with 19–28 sub-equal scales arranged in 3 rows. Segment X with complete saddle, apico-lateral margin dark with spicules; seta 1-X with 4 hyaline branches; seta 2-X with 1 long branch, 3 shorter; seta 3-X single; ventral brush (seta 4-X) with 5 pairs of 5-branched setae. Anal papillae slender, gradually tapering to apex. *Siphon*: long, at least 3 times longer than saddle, darker in mid-length; pecten with 18 spines on basal 0.3. Seta 1-S usually in 4 ventral pairs and 4 dorsal pairs; seta 2-S hook-shaped with small, curved secondary branch.

***Bionomics.*** Immature stages of *Cx. atratus* were collected in permanent and semi-permanent partially shaded habitats, such as ponds, stream margins, swamps and ditches, in association with herbaceous vegetation such as reeds, grass and algae, in fresh, clear or dark water. Larvae and pupae were found in association with *Nyssorhynchus albimanus* Wiedemann, 1820 and *Anopheles grabhamii* Theobald, 1901 and less frequently with *Cx*. *nigripalpus* Theobald, 1901 *Uranotaenia socialis* Theobald, 1901 and *Ur*. *cooki* Root, 1937 [40]. Larvae and pupae of *Cx*. *atratus* were found in artificial containers with *Aedes albopictus* (Skuse, 1894) in Florida Key, Florida, USA [52]. In the Cayman Islands, Davies [51] found larvae in low saline mangroves. Females were collected during human landing collections in Jamaica [55].

**Remarks**

*Culex atratus* was described as a species of the genus *Culex* by Theobald [30] based on males and females from Jamaica. Afterwards, Theobald [33] transferred *Cx*. *atratus* to the newly created genus *Melanoconion*, based on the arrangement of wing scales. Dyar [34] selected *Cx*. *atratus* as the type of genus *Melanoconion*, and Howard et al. [35] described the immature stages. Dyar & Knab [31] described *Cx*. *falsificator* from adults collected in Cuba, and Bonne & Bonne-Wepster [37] synonymized *Cx*. *falsificator* with *Cx*. *atratus*, which was accepted by Edwards [20] and Belkin [39]. *Culex advieri* was described by Senevet [32] from males collected in Guadeloupe. Floch & Abonnec [46] associated and described larvae of *Cx*. *advieri* from Guadeloupe. Rozeboom & Komp [6] synonymized *Cx*. *advieri* with *Cx*. *atratus*; the synonymy was also recognized later by Belkin et al. [38] and Belkin [39]. In spite of the morphological similarity between the species of the Atratus Group, adults of *Cx*. *atratus* can be identified by the following combination of characters: wings dark-scaled; small patch of pale scales on upper mesokatepisternum; scutum with very narrow, bronzy scales; mesepimeron entirely dark, without median pale area; terga II-VIII with basolateral patches of white scales. Males can be readily distinguished from the other species of the Atratus Group by the presence of 4 or 5 broad, hyaline, flattened, apically curved setae arising ventromesally between the proximal and distal divisions of the gonocoxite. In addition, other characteristics of the male genitalia can be employed to identify *Cx*. *atratus*: lateral plate without apical process and lateral process directed dorsolaterally, and ninth tergal lobe pear-shaped with aciculate setae arising at basal 0.6. Fourth-instar larvae can be distinguished by having the scales of the comb of segment VIII of equal size and arranged in four irregular rows; seta 5-C reaching the insertion of seta 6-C; siphonal pecten spines with large coarse marginal denticles. *Culex atratus* pupae can be distinguished by having a V-shaped pinna and seta 9-VIII with 4 or 5 aciculate branches.

***Culex*(*Melanoconion*)*caribeanus*Galindo & Blanton, 1954**

1954: *Culex* (*Melanoconion*) *caribeanus* Galindo & Blanton, 1954: 244 [22] (♂) holotype ♂ deposited in the USNM. Type locality: Mojinga Swamp, Canal Zone, Panama.

*Culex* (*Melanoconion*) *caribeanus* of Pecor et al. (1992: 16, 124) [15] (distr., type info.); Kobayashi (1999: 9) [41] (♀, ♂, L, P); Hutchings et al. (2016: 7) [56] (distr.).

***Type material*:** Holotype, adult male mounted on slide with dissected male genitalia (USNM 01347) and paratype male mounted on slide with dissected male genitalia (USNM 01160) deposited in the Diptera Collection, National Museum of Natural History (USNM), Washington, DC, USA.

***Material examined*:** 4 specimens: 4 ♂G, 2 ♂: INPA, Brazil: Amazonas State, Ipixuna Municipality, Lago Grande, Seringal Recreio, Gregório River (− 7.16828, − 70.81847), coll. Hutchings et al. 2011, 20–21.v.2011, det. Hutchings & Sallum, 3.vii.2012 (ProN-021394, ♂, ♂G); coll. Hutchings et al. 2011, 18–19.v.2011, det. Hutchings & Sallum, 3.vii.2012 (ProN-025011, ♂, ♂G). Amazonas State, Barcelos Municipality, Ararinha, Padauari River (0.49704, − 64.05336), coll. Hutchings et al. 2010, 6–7.vi.2010, det. Hutchings & Sá, 01.iii.2017 (ProN-022837, ♂G). Amazonas State, Maués Municipality, Picada Pirarara, Abacaxis River (− 5.25258, − 58.69786), coll. Hutchings et al. 2008, 28–29.v.2008, det. Sá, 01.iii.2017 (ProN-002595, ♂G).

***Distribution*:***Culex caribeanus* has been found in Mojinga Swamp, Canal Zone, Panama [22] and in the Municipalities of Maués (as *Cx*. *trigeminatus*) [57], Barcelos [56] and Ipixuna in Amazonas State, Brazil (present study).

**Description**

**Fig. 7:**
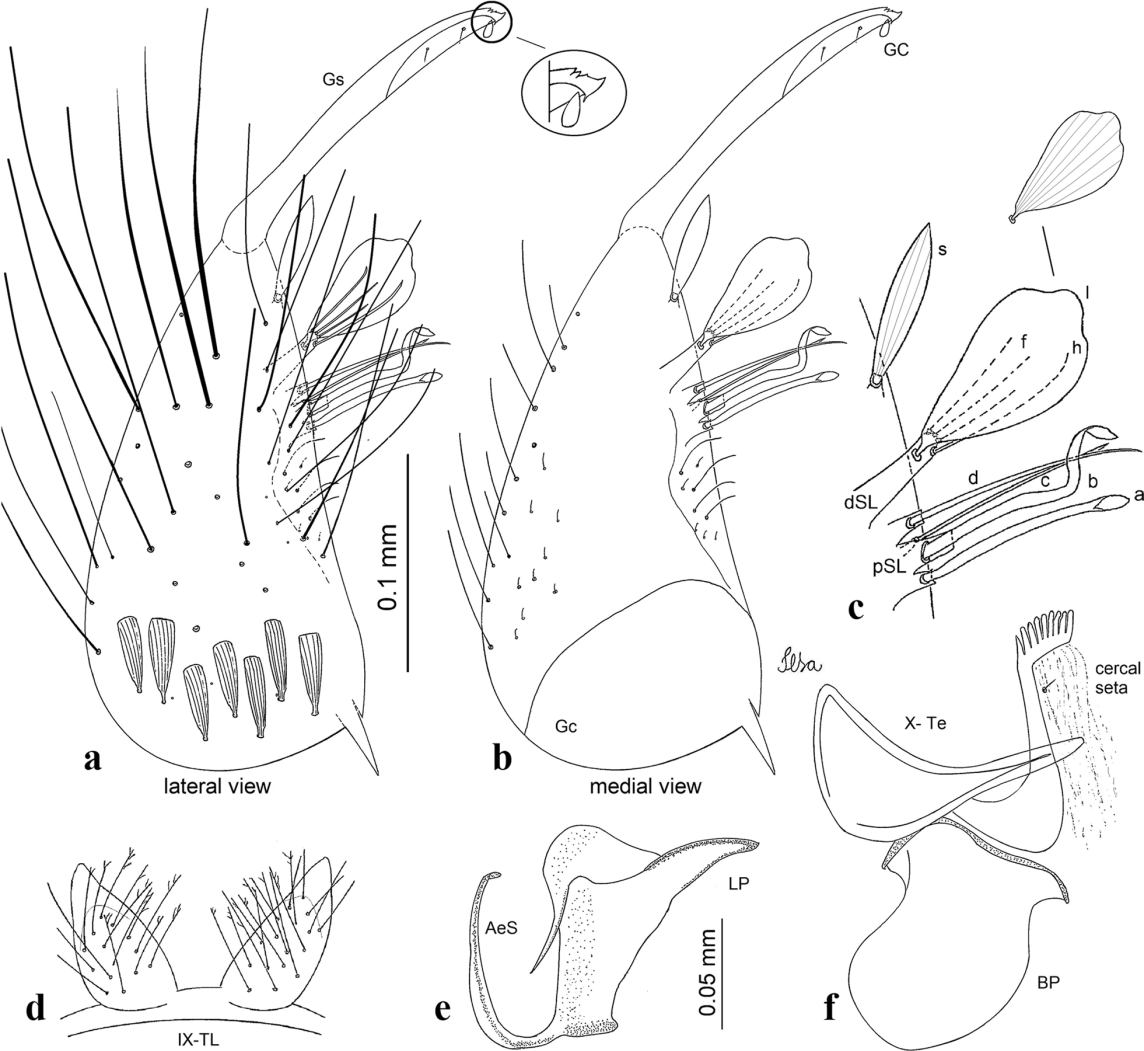
*Culex* (*Melanoconion*) *caribeanus*, male genitalia. **a** Gonocoxite in lateral view. **b** Gonocoxite in medial view. **c** Setae on the subapical lobe of the gonocoxite. **d** Tergum IX lobe. **e** Aedeagus. **f** Proctiger. *Abbreviations*: Gc, gonocoxite; Gs, gonostylus; GC, gonostylar claw; dSL, distal division of subapical lobe; pSL, proximal division of subapical lobe; IX-TL, tergum IX lobe; AeS, aedeagal sclerite; LP, lateral plate; BP, basal plate; X-Te, tergum X

***Male.*** [Figs. 2b, 7] *Head*: antenna dark, verticillate, length 1.04–1.38 (1.21) (*n* = 2); proboscis dark-scaled, with conspicuous, median, dorsal patch of whitish scales, length 1.30–1.51 (1.40) (*n* = 2); maxillary palpus length 1.69–1.90 (1.79) (*n* = 2); palpomere I entirely whitish-scaled; palpomere II with basal patch of whitish scales; palpomere III with conspicuous patch of whitish scales on median portion. *Thorax*: scutum covered with bronzed scales, except whitish scales on anterior promontory, scutal fossa, dorsocentral and supraalar areas forming a pattern. Scutellar scales whitish; median lobe with 4 or 5 setae; lateral lobes with 3 or 4 setae each. Pleural setae with 2 types of colouring: dark brown with bronzy reflections: 7 or 8 antepronotal, 5 or 6 prealar; and pleural setae golden, hyaline: 4 or 5 upper mesokatepisternal, 3 or 4 lower mesokatepisternal, 5 or 6 upper mesepimeral, and 1 large lower mesepimeral. Pleura with patch of broad, white scales on upper mesokatepisternum; lower mesokatepisternum with few scales not forming patch. *Wing*: dark-scaled as in *Cx*. *atratus*; length 2.31–2.47 (2.39) mm (*n* = 2). *Halter*: scabellum and pedicel whitish; capitellum brown with few scales with golden reflections. *Legs*: fore- and midfemora with preapical ring of white scales. *Abdomen*: tergum I dark-scaled; terga III-VIII dark-scaled with basal bands of white scales. *Genitalia*: tergum IX lobes with concave inner margin, pointed and apex glabrous, median portion each with 14–16 slender, simple and aciculate setae; distance between lobes smaller than half basal width of 1 lobe. Gonocoxite narrow, oblong; proximal division of subapical lobe with 4 parallel, apically pointed setae (setae *a*, *b*, *c* and *d*): seta *a* more basal, spoon-shaped; seta *b* longer than others, spatulate, sinuous subapically; seta *c* thin, slender, filiform, inserted between setae *b* and *d*; seta *d* implanted on tubercle apical to seta *b*, filiform, long. Distal division with elongated columnar process, with 5 setae: 3 filiform, narrow, pointed, apically inserted, subequal in size (setae *f*), 1 filiform, with hook-like apex (seta *h*) and 1 large, broad, asymmetrical, ribbed seta arising subapically (*l* seta); 1 saber-like, ribbed seta (seta *s*) arising apically. Gonocoxite with 4 or 5 slender, hyaline, short, inconspicuous setae borne ventromesally between proximal and distal divisions. Gonostylus as in *Cx*. *atratus*, except on dorsal surface of apex with 2 or 3 inconspicuous folds. Ventral process of lateral plate with large convexity on upper border and conspicuous pointed projection directed ventrobasally. Proctiger with tergum X somewhat triangular in outline, inner process pointed and long.

***Female, pupa and larva.*** Unknown.

***Bionomics.*** Adult males were collected using CDC light traps with UV lamps in upland (*terra firme*) Amazon Forest [56].

**Remarks**

*Culex caribeanus* was described by Galindo & Blanton [22] from males collected in the Canal Zone, Panama. The adult female, fourth-instar larva and pupa have not been described. Recently, Hutchings et al. [56] found the species for the first time in Brazil, in the Amazon Forest. Adults of *Cx*. *caribeanus* can be misidentified as *Cx*. *trigeminatus* if the male genitalia are not properly dissected and mounted in lateral view. Based on characteristics of the female, *Cx*. *caribeanus* is similar to *Cx*. *trigeminatus* in possessing preapical rings of white scales on the fore- and midfemora and proboscis with patch of whitish scales on the median portion of ventral surface. *Culex caribeanus* differs from *Cx*. *trigeminatus* in having palpomere I entirely white-scaled, palpomere II with basal patch of white scales, palpomere III with conspicuous patch of white scales on median portion close to pale patch of proboscis, and wings entirely dark-scaled on ventral and dorsal surfaces. In *Cx*. *trigeminatus*, the palpomeres I and II are dark-scaled, palpomere III has a small basal patch of pale scales, and palpomere IV has an inconspicuous proximal patch of whitish scales, and the wings have veins C and R with basal patches of white scales. The male genitalia of *Cx*. *caribeanus* have simple and aciculate setae on the median portion of the ninth tergal lobes, whereas in *Cx*. *trigeminatus* the setae are simple. In addition, *Cx. caribeanus* differs from *Cx*. *trigeminatus* in possessing a pronounced convexity on the apical margin of the lateral plate, and a conspicuous pointed projection directed ventrobasally in the ventral process. In *Cx. trigeminatus*, the apical margin of the ventral process of the lateral plate is straight and bears a short projection.

***Culex*(*Melanoconion*)*columnaris*Sá & Hutchings n. sp.**

***Type locality*:** Senador Guiomard Municipality in Fazenda Experimental Catuaba, UFAC, BR-364 Km 23 (− 10.05739, − 67.60013), Acre State, Brazil. Adults were collected using a CDC trap with UV light in *terra firme* forests at an elevation of 205 m.

***Type material*:** Holotype, pinned adult male with associated dissected genitalia on slide (specimen field no. rBIA-000462, accession no. INPA-DIP 004565), with following collection data: Brazil: Acre State, Senador Guiomard Municipality, Fazenda Experimental Catuaba, UFAC, BR-364 Km 23 (− 10.05739, − 67.60013), coll. Hutchings & Carmo, 23-24.viii.2016, det. Sá, 2017, deposited in the Coleção de Invertebrados, Instituto Nacional de Pesquisas da Amazônia (INPA), Manaus, Amazonas State, Brazil. Paratypes: 2 pinned adult males with dissected genitalia on separate slides (specimen field no. rBIA-000467, accession no. INPA-DIP 004566 and specimen field no. rBIA-000469, accession no. INPA-DIP 004567) from same collection as holotype and deposited in INPA; and 2 pinned adult males with dissected genitalia on separate slides (specimen field no. rBIA-000470, accession no. FSP-USP E-15905 and specimen field no. rBIA-000472, accession no. FSP-USP E-15906), from same collection as holotype and deposited in the Coleção Entomológica de Referência, Faculdade de Saúde Pública, Universidade de São Paulo (FSP-USP), São Paulo State, São Paulo municipality, Brazil.

***ZooBank registration*:** The Life Science Identifier (LSID) for *Culex* (*Melanoconion*) *columnaris* n. sp. is urn:lsid:zoobank.org:act: 139D4046-50EC-4D2A-AE1B-51502EC47A44.

***Etymology*:** From the Latin adjective *columnaris*, meaning rising in the form of column, in reference to the long columnar process of the proximal division of the subapical lobe.

**Description**

**Fig. 8:**
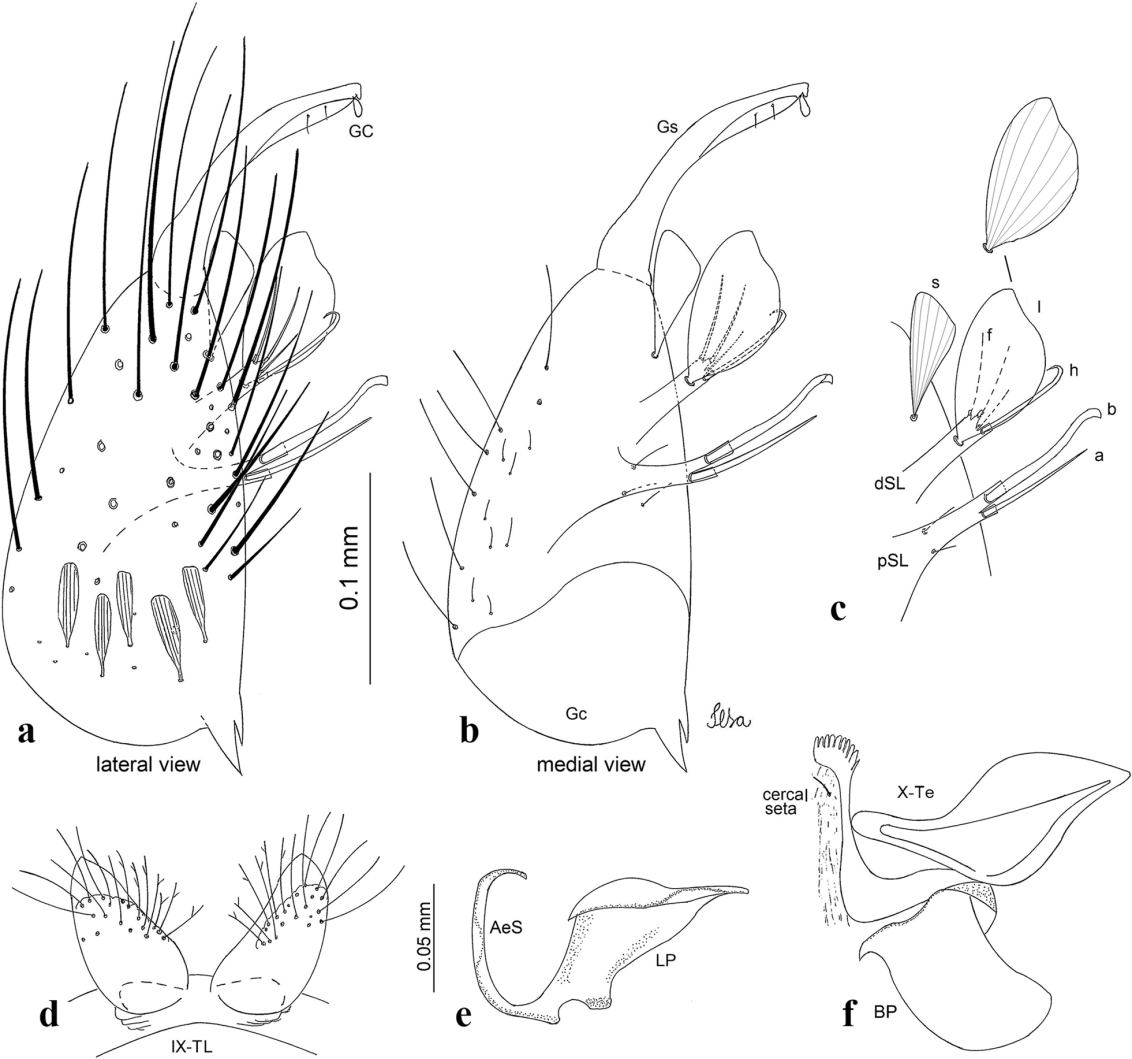
*Culex* (*Melanoconion*) *columnaris* n. sp., male genitalia. **a** Gonocoxite in lateral view. **b** Gonocoxite in medial view. **c** Setae on the subapical lobe of the gonocoxite. **d** Tergum IX lobe. **e** Aedeagus. **f** Proctiger. *Abbreviations*: Gc, gonocoxite; Gs, gonostylus; GC, gonostylar claw; dSL, distal division of subapical lobe; pSL, proximal division of subapical lobe; IX-TL, tergum IX lobe; AeS, aedeagal sclerite; LP, lateral plate; BP, basal plate; X-Te, tergum X

***Male***. [Figs. 2c, 8] Integument dark brown, with pale areas on thoracic pleura. *Head*: antennal length 1.06–1.60 (1.40) (*n* = 5); proboscis entirely dark-scaled, length 1.44–1.70 (1.55) (*n* = 5); maxillary palpus dark-scaled, length 1.82–2.21 (1.96) (*n* = 5); occiput with dark brown erect forked scales. *Thorax*: scutum with narrow, brown, falcate scales with golden reflection, prescutellar area with whitish scales. Scutellar scales whitish, median lobe with 6 setae; lateral lobes each with 3 or 4 large setae. Pleural setae with 2 types of colouring: dark brown: 3–5 antepronotal, 4–6 prealar; and pleural pale golden, slender setae: 4 or 5 upper mesokatepisternal, 3 or 4 lower mesokatepisternal, 4 or 5 upper mesepimeral; lower mesepimeron with one strong, long, pale golden seta. Mesepimeral integument dark, with indistinct pale spot on median area, not dividing upper and lower areas. Pleura with less evident patch of broad, white scales on upper mesokatepisternum; lower mesokatepisternum with few white scales. *Wing*: dark-scaled; length 2.19–2.54 (2.35) (*n* = 5). *Halter*: scabellum, pedicel and capitellum whitish. *Legs*: coxae pale; ventral surface of fore- and midfemur with a longitudinal stripe of white scales; tibiae dark-scaled; joints of femur-tibia and tibia-tarsomere I with ring of pale scales; tarsi entirely dark-scaled. *Abdomen*: tergum I with dark scales; terga III-VII dark-scaled with proximal white bands; tergum VIII with dark scales. *Genitalia*: tergum IX lobes elongate, each with 14 slender, apically bifid, simple setae in median portion; apex glabrous. Distance between lobes about 1/3 of basal width of 1 lobe. Gonocoxite oblong, narrow; proximal division with long, apically divided, columnar process bearing 2 parallel setae (*a* and *b*): seta *a* basal, slender with pointed apex; seta *b* slightly sinuous with apex curved. Distal division with long columnar process with 5 setae: 3 narrow, filiform, apically pointed setae of different sizes (seta *f*), 1 long seta hook-like at apex (seta *h*), and 1 large, long, asymmetrical seta arising subapically (seta *l*); 1 saber-like seta with broad apex and without peduncle on base (seta *s*) arising apically; gonocoxite with 2 short, hyaline setae on ventromesal surface. Gonostylus slender, slightly curved, with moderately pointed apex, ventral surface with 2 apical hyaline setae; 1 short gonostylar claw arising apically. Aedeagus with apical process slightly curved dorsally and ventral process with rounded prominence. Proctiger with tergum X asymmetrical, with outer process rounded.

**Remarks**

The male genitalia and adults of *Cx*. *columnaris* n. sp. bear more morphological similarities to *Cx*. *zeteki* than to other species of the Atratus Group. However, adult specimens of the new species differ from *Cx*. *zeteki* in having the mesepimeron with a slightly light stain on the median area, not divided into upper and lower areas. The male genitalia differ from those of *Cx*. *zeteki* in having the seta *s* without peduncle on base and with a broad apex, IX tergal lobes with bifid setae in the ventromedial region, and a lateral plate without undulations on the apical process. Furthermore, *Cx*. *columnaris* n. sp. has the proximal division of subapical lobe with an apically divided long columnar process which bears only two setae (*a* and *b*).

***Culex*(*Melanoconion*)*comptus*Sá & Sallum n. sp.**

***Type locality*:** Presidente Epitácio Municipality near Horto Florestal (− 21.759401, − 52.09677), São Paulo State, Brazil. Larvae were collected in partially shaded, permanent habitats, with turbid water, associated with *Pistia* sp., in remnants of the Atlantic Forest and in transition areas between the Cerrado and the Atlantic Forest biomes, cohabiting with *Cx*. *dunni*.

***Other localities*:** Bolivia, Brazil, Panama and Suriname. In Brazil, the species occurs in the municipalities of Presidente Epitácio and Dourado, São Paulo State, in Santo Antônio do Içá, Manacapuru and Jutaí, Amazonas State, and in the municipality of Juruti, Pará State (present study).

***Type material*:** Holotype, pinned adult male with dissected genitalia, larval and pupal exuviae on the same slide (specimen field no. SP172-30, accession no. FSP-USP E-15881), with following collection data: Brazil, São Paulo State, Presidente Epitácio Municipality, near Horto Florestal (− 21.759401, − 52.09677), coll. Sá & Chaves, 15.iii.2016, det. Sá, 2016, deposited in the Coleção Entomológica de Referência, Faculdade de Saúde Pública, Universidade de São Paulo (FSP-USP), São Paulo Municipality, São Paulo State, Brazil. Paratypes: 2 pinned adult males with dissected genitalia, larval and pupal exuviae on separate slides (specimen field no. SP172-41, accession no. FSP-USP E-15883 and specimen field no. SP172-44, accession no. FSP-USP E-15885); 2 pinned adult females associated with larval and pupal exuviae on separate slides (specimen field no. SP172-29, accession no. FSP-USP E-15882 and specimen field no. SP172-31, accession no. FSP-USP E-15884); 2 pinned adult males with dissected genitalia and pupal exuviae on separate slides (specimen field no. SP172-118, accession no. FSP-USP E-15886 and specimen field no. SP172-119, accession no. FSP-USP E-15887); 1 pinned adult female with associated pupal exuviae on slide (specimen field no. SP172-122, accession no. FSP-USP E-15888), from same collection as holotype and deposited in FSP-USP; 2 pinned adult males with dissected genitalia on separate slides (specimen field no. ProV-004340, accession no. INPA-DIP 004578 and specimen field no. ProV-005250, accession no. INPA-DIP 004579), with following collection data: Brazil, Amazonas State, Santo Antônio de Içá Municipality, Parana do Canini, Solimões River (− 3.15123, − 68.00142), coll. Hutchings et al. 2003; 1 pinned adult male with dissected genitalia on slide (specimen field no. ProV-006561, accession no. INPA-DIP 004580), with following collection data: Brazil, Amazonas State, Jutai Municipality, São Raimundo, Parana do Cervalho, Solimões River (− 2.70907, − 66.89931), coll. Hutchings et al. 2003; 1 pinned adult male with dissected genitalia on slide (specimen field no. ProV-045575, accession no. INPA-DIP 004581), with following collection data: Brazil, Amazonas State, Manacapuru Municipality, Parana do Cururu, Solimões River (− 3.5753, − 60.80877), coll. Hutchings et al. 2003; and 1 pinned adult male associated with dissected male genitalia (specimen field no. ProV-053604, accession no. INPA-DIP 004582), with following collection data: Brazil, Pará State, Juruti Municipality, Recreio, Parana de Dona Rosa, Amazon River (− 2.07554, − 55.96586), coll. Hutchings et al. 2003, deposited in the Coleção de Invertebrados, Instituto Nacional de Pesquisas da Amazônia (INPA), Manaus, Amazonas State, Brazil.

***Material examined*:** 21 specimens: 10 ♂G, 16 Pe, 14 Le, 4 ♂, 10 ♀. FSP-USP, Brazil, São Paulo State, Presidente Epitácio Municipality (− 21.759401, − 52.09677), coll. Sá & Chaves, 15.iii.2016, det. Sá, 2016: SP172-06 (Le, Pe, ♀); SP172-17 (Le, Pe, ♂G, ♂); SP172-28 (Le, Pe, ♀); SP172-32 (Le, Pe, ♂G, ♂); SP172-34 (Le, Pe, ♂G, ♂); SP172-36 (Le, Pe, ♀); SP172-37 (Le, Pe, ♀); SP172-39 (Le, Pe, ♀); SP172-43 (Le, Pe, ♀); SP 172-45 (Le, Pe, ♀); SP172-46 (Le, Pe, ♀); SP172-47 (Le, Pe, ♀); SP172-49 (Le, Pe, ♀); SP172-110 (Pe, ♂G, ♂). São Paulo State, Dourado Municipality, SP255Km, Obelisco (− 24.075694, − 48.437361) coll. Sallum et al., 7.v.2009, det Sallum 2012: E-15439 (Pe). Dourado, SP255 km, Santa Leonor Farm (− 24.074417, − 48.444389), coll. Sallum et al. 7.v.2009, det. Sallum 2012: E-15440 (Le, Pe, ♂G). USNM, Panama: PA37-115 (♂G); PA2-101 (♂G). Suriname: (USNM) S.S det., 1978: MEP-AC634-20 (♂G); MEP-AC634-21 (♂G). Bolivia: Catalog no. 82164 (♂G).

***ZooBank registration*:** The Life Science Identifier (LSID) for *Culex* (*Melanoconion*) *comptus* n. sp. is urn:lsid:zoobank.org:act: 260B62E3-BD5B-4384-980A-AD7000EC8E17.

***Etymology*:** From the Latin adjective *comptus*, meaning ornate, adorned, in reference to the dark brown to black and pale golden scales that form a pattern on the scutum.

**Description**

***Female*.** Integument dark brown, with pale areas on thoracic pleura. *Head*: antenna dark, flagellum normal, whorls with 5 setae, length 1.00–1.38 (1.21) (*n* = 5); proboscis dark-scaled, length 1.35–1.44 (1.42) (*n* = 5); maxillary palpus dark-scaled, length 0.21–0.26 (0.24) (*n* = 5). Occiput with dark brown erect forked scales. *Thorax:* integument dark brown; scutum covered with narrow, dark brown to black falcate scales; may have pale golden scales forming a pattern on anterior promontory, scutal fossa, dorsocentral, prescutelar and supraalar areas. Scutellar scales whitish; median lobe with 5 or 6 setae; lateral lobes each with 3 or 4 setae. Pleural setae with 2 types of colouring: brown, large: 3–7 antepronotal, 3–5 prealar; and pleural setae golden, hyaline: 4 or 5 upper mesokatepisternal, 3 or 4 lower mesokatepisternal, 4 or 5 upper mesepimeral and 1 large lower mesepimeral. Pleura with patch of broad, white scales on upper mesokatepisternum; lower mesokatepisternum with few white scales. *Wing*: dark-scaled; vein C with small proximal patch of white scales, vein Sc occasionally with inconspicuous proximal patch of white scales, vein R with proximal patch of white scales separated by median patch of dark scales; wing length 2.44–3.05 (2.79) (*n* = 5). *Halter*: scabellum and pedicel whitish; pedicel with narrow, brown, dorsal strip; capitellum brownish. *Legs*: coxae pale; ventral surface of fore- and midfemur with longitudinal stripe of white scales; tibiae dark-scaled; joints of femur-tibia and tibia-tarsomere I with ring of pale scales; tarsi entirely dark-scaled. *Abdomen*: tergum I dark-scaled; terga II-VIII dark-scaled with basal bands of white scales.

**Fig. 9:**
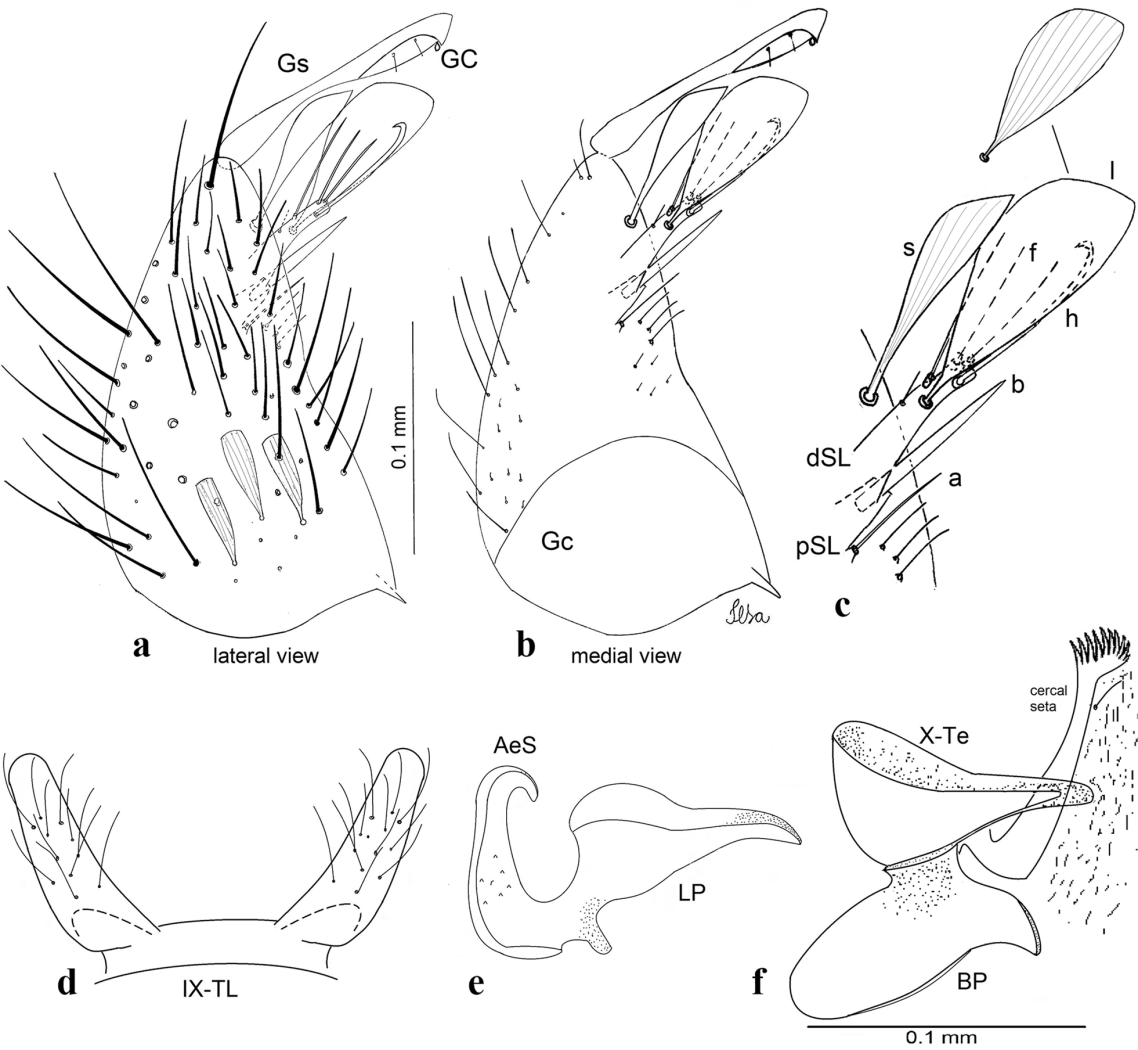
*Culex* (*Melanoconion*) *comptus* n. sp., male genitalia. **a** Gonocoxite in lateral view. **b** Gonocoxite in medial view. **c** Setae on the subapical lobe of the gonocoxite. **d** Tergum IX lobe. **e** Aedeagus. **f** Proctiger. *Abbreviations*: Gc, gonocoxite; Gs, gonostylus; GC, gonostylar claw; dSL, distal division of subapical lobe; pSL, proximal division of subapical lobe; IX-TL, tergum IX lobe; AeS, aedeagal sclerite; LP, lateral plate; BP, basal plate; X-Te, tergum X

***Male*.** [Figs. 2d, 9] Essentially as female, except for following characters: *Head*: antenna verticillate, length 0.92–1.37 (1.10) (*n* = 5); proboscis length 1.48–1.59 (1.55) (*n* = 5); maxillary palpus length 1.54–2.16 (1.89) (*n* = 5). *Wing*: length 2.37–2.63 (2.51) (*n* = 5). *Genitalia*: tergum IX lobes somewhat conical, elongate, narrow with glabrous apex, median portion each with 11–15 slender, simple setae; distance between lobes larger than basal width of 1 lobe. Gonocoxite oblong; proximal division of subapical lobe with 2 parallel, pointed setae (*a* and *b*): seta *a* basal, short, narrower than seta *b*, with pointed apex; seta *b* long, spatulate, with pointed apex and implanted on salient tubercle. Distal division with long columnar process, with 5 setae: 3 filiform, narrow, pointed, apically inserted, subequal in size (seta *f*); 1 filiform, with hooked apex (seta *h*); 1 large, long, broad, asymmetrical, ribbed seta arising subapically (seta *l*); and 1 saber-like, ribbed seta (seta *s*) arising apically. Gonocoxite with 3 or 4 hyaline, filiform, median, inconspicuous setae on ventromesal surface. Gonostylus slender, slightly curved, with moderately blunt apex, ventral surface with 2 apical hyaline setae, gonostylar claw extremely short. Aedeagal sclerite with few, inconspicuous spicules on ventral surface; lateral process pointed and directed dorsolaterally. Proctiger with tergum X somewhat triangular in outline, inner process pointed.

***Pupa*.** [Figs. 3b, 4b] *Cephalothorax*: trumpet cylindrical, pinna small, irregular in shape, pinna length 0.07–0.12 (0.10) (*n* = 10), distal margin opposite meatal cleft, with small emargination; tracheoid area darker, extending 0.20–0.25 (0.23) (*n* = 10) from base; trumpet index 13.8–16.2 (15.4) (*n* = 10). *Abdomen*: seta 9-VIII with 3 simple branches; paddle index 1.32–1.57 (1.47) (*n* = 10).

***Larva*.** [Figs. 5b, 6b] *Head*: length 0.62–0.71 (0.67) (*n* = 10), width 1.04–1.10 (1.06) (*n* = 5). Antennal length 0.49–0.60 (0.55) (*n* = 10). Seta 1-A inserted 0.38–0.40 (0.38) (*n* = 10) from antennal base; seta 4-C double; seta 5-C with 3 or 4 long branches reaching 6-C insertion; seta 13-C double. *Abdomen*: comb of segment VIII with 25–28 sub-equal scales arranged in 3 rows. Segment X length 0.34–0.38 (0.36) (*n* = 10), saddle complete, apicolateral margin dark with spicules; siphon/saddle index 4.17–5.18 (4.67) (*n* = 10). *Siphon*: long, slender, index 8.7–11.0 (9.7) (*n* = 10); pecten with 12 marginal spines on basal 0.30 of siphon. Seta 1-S usually with 4 ventral pairs and 4 dorsal pairs.

**Remarks**

Based on the original description of Bonne-Wepster & Bonne [58], and illustrations presented by Bonne & Wepster-Bonne [37], Sirivanakarn [8] and Pecor et al. [15], the male genitalia of *Cx*. *comptus* n. sp. are more morphologically similar to *Cx*. *commevynensis* than to any other species of the Atratus Group. These species have in common characteristics such as the shape and size of seta *l* of the distal division, the length of the columnar process of the distal division and the number and form of setae in the proximal division. However, the male genitalia of *Cx*. *comptus* n. sp. differ from those of *Cx*. *commevynensis* in possessing slightly conical, narrow and elongate tergum IX lobes. On the other hand, in the original description of the *Cx*. *commevynensis* adult, Bonne-Wepster & Bonne [58] mention the presence of pale golden, narrow and curved scales on the occiput, and pale golden brown, narrow curved scales on the anterior half of scutum and brown on the posterior half. In *Cx*. *comptus* n. sp., the vertex and occiput possess whitish, curved, narrow scales and the scutum is covered with narrow and dark brown to black falcate scales and some pale golden scales forming conspicuous pattern on the anterior promontory, scutal fossa, dorsocentral, prescutelar and supraalar areas. The fourth-instar larva of *Cx*. *comptus* n. sp. differs from the larva of *Cx*. *zeteki* in having three rows of comb scales and slender pecten spines with serrate edges. The pupa of *Cx*. *comptus* n. sp. differs from the pupa of *Cx*. *zeteki* in having the pinna of the trumpet slightly smaller and differs from the pupa of *Cx*. *trigeminatus* in having the trumpet clearer and with the distal margin bearing a more conspicuous notch opposite the meatal cleft.

***Culex*(*Melanoconion*)*dunni*Dyar, 1918**

1918: *Culex* (*Melanoconion*) *dunni* Dyar, 1918: 123 [59] (♂, ♀) lectotype ♂, ♂G, deposited in the USNM. Type locality: Mandingo River, Panama.
1924: *Culex* (*Melanoconion*) *ruffinis* Dyar & Shannon, 1924: 144 [60] (♂, ♂G) lectotype ♂, ♂G deposited in the USNM. Type locality: Barro Colorado Island, Gatun Lake, Canal Zone, Panama.

*Culex* (*Melanoconion*) *dunni* of Dyar (1923: 188) [36] (synonymy with *Cx*. *ensiformis*); Dyar (1928: 340) [19] (♂, ♀, ♂G, L); Komp (1935: 8) [21] (♂G); Foote (1954: 42) [7] (L, P); Stone & Knight (1957: 48) [45] (desig. syntype); Pecor et al. (1992: 21) [15] (distr.); Williams et al. (2007: 78) [61] (Guatemala); Berti et al. (2013: 5) [62] (Venezuela); Torres-Gutierrez & Sallum (2015: 16) [10] (distr.).

*Culex* (*Melanoconion*) *ruffinis* of Dyar (1928: 341) [19] (♂, ♀, ♂G, L); Komp (1935:08) [21] (synonymy with *Cx*. *dunni*).

***Type material*:** Lectotype pinned adult male in good condition (USNM no. 21714), with dissected genitalia on slide (USNM no. 901).

***Material examined*:** 56 specimens: 45 ♂G, 15 Le, 18 Pe, 18 ♂, 16 ♀. FSP-USP, Brazil: São Paulo State, Cananéia Municipality, Vilarinho (− 24.951551, − 47.977989), coll. Sá et al. 14.vii.2015, det. Sá 2015: SP166-03 (Le, Pe); SP166-04 (Pe); SP166-21 (Le, Pe, ♂G). São Paulo State, Presidente Epitácio Municipality (− 21.759401, − 52.09677), coll. Sá & Chaves 2016, det. Sá 2016: SP172-35 (Le, Pe, ♂G, ♂). São Paulo State, Cananéia Municipality, Itapitangui (− 24.935105, − 47.961728), coll. Forattini et al. 1985, det. Sallum 1985: EP014-1 (Le, Pe, ♀); EP058-1 (Pe, ♂G, ♂); EP058-3 (Le, Pe, ♂G); EP070-1 (Le, Pe, ♂G, ♂). São Paulo State, Dourado (− 22.100000, − 48.317778), coll. Forattini et al. 1980, det. Sallum 1980: 02 (♂G); 28 (♂G); 148 (♂G, ♂). São Paulo State, Pariquera-Açu Municipality, Pariquera-Mirim (− 24.729867, − 47.813300), coll. Forattini et al. 24.vii.1984, det. Sallum 1984: HEP414-7 (Le, Pe, ♂G); HEP414-8 (Le, Pe, ♂G); HEP414-9 (Le, Pe, ♀); HEP414-18 (Le, Pe, G♂ ♂); HEP414-20 (Le, Pe, ♀); HEP429-3 (Le, Pe); HEP429-7 (Le, Pe, ♂G); HEP440-7 (Le, Pe, ♀). Minas Gerais State, Goianá Municipality (− 21.538836, − 43.350856), coll. Bergo et al. 30.xi.2008, det. Sallum 2008: MG24-102 (Pe, ♂G), Minas Gerais State, Carmo da Mata Municipality, Rural das Pedras Farm (− 20.545264, − 44.859047), coll. Bergo et al. 13.iv.2010, det. Sallum 2014: MG46-02 (Le, Pe). INPA, Brazil: Pará State, Almeirim Municipality, Arumanduba, Amazon River (− 1.48631, − 52.48706), coll. Hutchings et al. 19–20.viii.2003, det. Hutchings: ProV-047641 (♂G); ProV-047649 (♂G); ProV-047741 (♂G). Pará State, Almeirim Municipality, Paraiso, Paranacuara, Amazon River (− 1.74512, − 53.154), coll. Hutchings et al. 21–22.viii.2003, det. Hutchings: ProV-055512 (♂G). Pará State, Prainha Municipality, Fazenda JK, Amazon River (− 1.86209, − 53.72193), coll. Hutchings et al. 22–23.x.2003, det. Hutchings & Sá: ProV-049099 (♂G). Pará State, Prainha Municipality, Curuauna River (− 2.39349, − 54.08755), coll. Hutchings et al. 24–25.x.2003, det. Hutchings & Sá: ProV-049615 (♂G). Pará State, Juruti Municipality, Recreio, Parana de Dona Rosa, Amazon River (− 2.07554, − 55.96586), coll. Hutchings et al. 30–31.x.2003, det. Sallum, Hutchings & Sá: ProV-053664 (♂G); ProV-053667 (G); ProV-053670 (G); ProV-053674 (G); ProV-053678 (♂G); ProV-053679 (♂G); ProV-053688 (♂G). Amazonas State, Iranduba Municipality, Ramal do Lago Grande (− 3.1983, − 60.28233), coll. Hutchings et al. 8–10.ix.2008, det. Hutchings: IRam-000751 (♂G); IRam-000752 (♂G); IRam-000759 (♂G); IRam-000760 (♂G); IRam-000945 (♂G); IRam-002062 (♂G); IRam-002061 (♂G); IRam-002060 (♂G); IRam-001933 (♂G); IRam-001932 (♂G); IRam-001931 (♂G); IRam-001212 (♂G). Amazonas State, Juruá Municipality, Igarapé de Tamaniqua, Solimões River (− 2.66104, − 65.74101), coll. Hutchings et al. 19.ix.2003, det. Sallum, Hutchings & Sá: ProV-015891 (♂G); ProV-015900 (♂G). Amazonas State, Jutaí Municipality, São Raimundo (− 2.70907, − 66.98831), coll. Hutchings et al. 16–17.ix.2003, det. Sá 1.iii.2017: ProV-006542 (♂G). Amazonas State, Urucara Municipality, Lírio do Vale (− 2.42571, − 57.5024), coll. Hutchings et al. 3.xi.2003, det. Sallum, Hutchings & Sá: ProV-056752 (♂G). Synonym species *Culex ruffinis*: lectotype, pinned adult male in good conditions and male genitalia (USNM no. 1928), deposited in the Diptera Collection, National Museum of Natural History (USNM), Washington, DC, USA.

***Distribution*:***Culex dunni* has been found in Central and South America, including Belize [63], Brazil [10], Colombia [64, 65], Costa Rica [15], French Guiana [66, 67], Guatemala [61], Mexico [14, 16], Nicaragua [15], Panama [15], Suriname [69] and Venezuela [53, 62, 68]. In Brazil, *Cx*. *dunni* was collected in the municipalities of Iranduba, Juruá, Jutaí, Manaus and Urucará, Amazonas State; in Bataguassu Municipality, Mato Grosso do Sul State; Carmo da Mata and Goianá Municipalities, Minas Gerais State; Almeirim, Juruti and Prainha Municipalities, Pará State; Cananéia, Dourado, Pariquera-Açu, and Presidente Epitácio Municipalities in São Paulo State (present study).

**Description**

***Female*.***Head*: antenna dark, flagellum normal, whorls with 4 or 5 setae, length 1.21–1.35 (1.26) (*n* = 5); proboscis dark-scaled, length 0.58–1.44 (1.19) (*n* = 5); maxillary palpus dark-scaled, length 0.18–0.19 (0.19) (*n* = 5). Occiput with dark brown erect forked scales. *Thorax*: integument brown; scutum covered with narrow, bronze falcate scales; possibly with whitish scales on anterior promontory, scutal fossa, dorsocentral and supraalar areas, but not forming a pattern. Scutellar scales withish; median lobe with 5 or 6 setae; lateral lobes each with 3 or 4 setae. Pleural setae with 2 types of colouring: brown with bronzy reflections: 5–7 antepronotal, 3–5 prealar; and pleural setae golden, hyaline: 5 or 6 upper mesokatepisternal, 4 or 5 lower mesokatepisternal, 4 or 5 upper mesepimeral, and 1 large lower mesepimeral. Pleura with patch of broad, white scales on upper mesokatepisternum; lower mesokatepisternum with few white scales. *Wing*: dark-scaled, vein R with 2 proximal patches of white scales separated by large patch of dark scales; occasionally vein C with small proximal patch of white scales; length 2.67–3.00 (2.86) (*n* = 5). *Halter*: scabellum, pedicel and capitellum brownish. *Legs*: as in *Cx*. *atratus*. *Abdomen*: tergum I dark-scaled; terga III-VIII dark-scaled with basal bands of white scales.

**Fig. 10:**
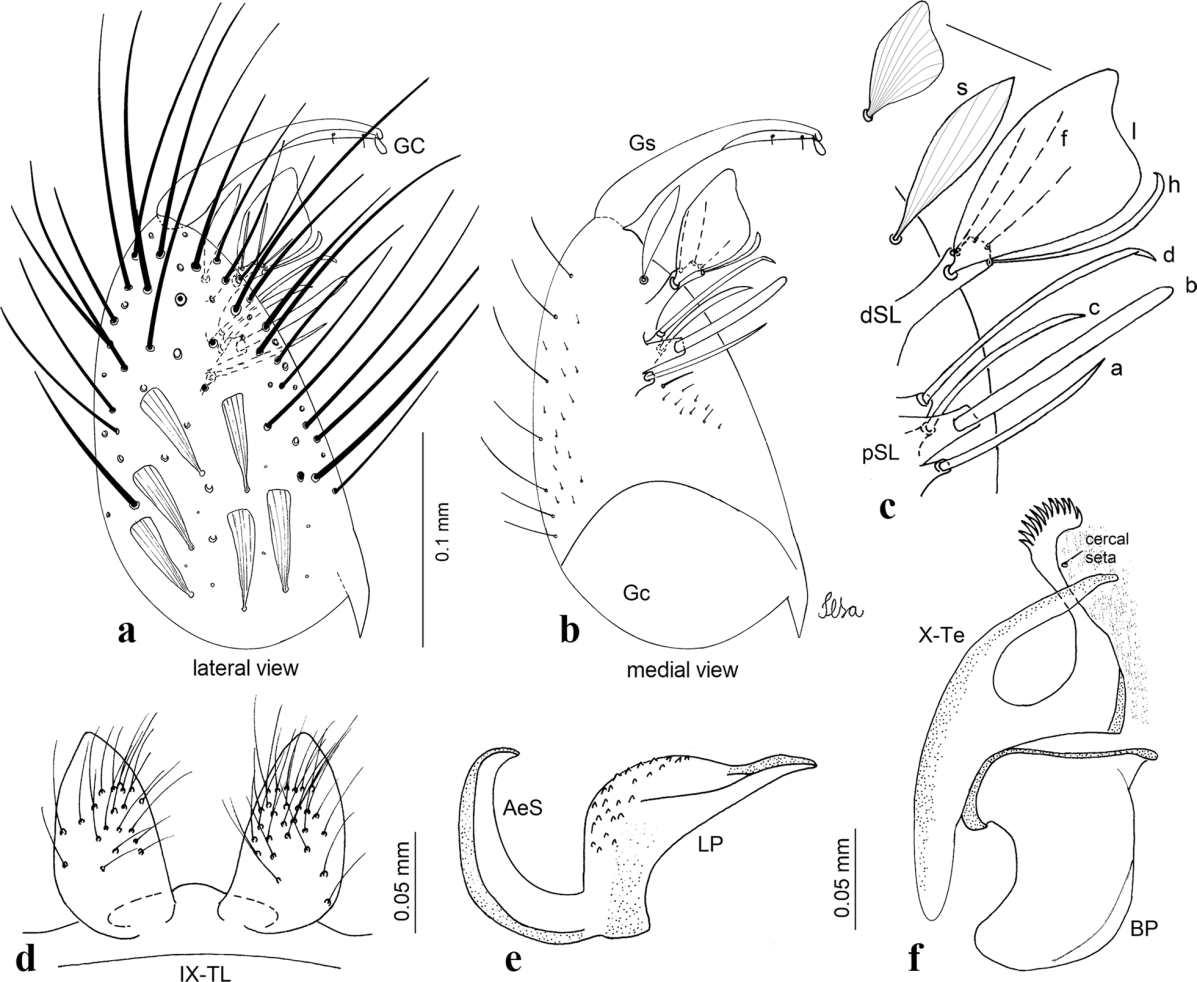
*Culex* (*Melanoconion*) *dunni*, male genitalia. **a** Gonocoxite in lateral view. **b** Gonocoxite in medial view. **c** Setae on the subapical lobe of the gonocoxite. **d** Tergum IX lobe. **e** Aedeagus. **f** Proctiger. *Abbreviations*: Gc, gonocoxite; Gs, gonostylus; GC, gonostylar claw; dSL, distal division of subapical lobe; pSL, proximal division of subapical lobe; IX-TL, tergum IX lobe; AeS, aedeagal sclerite; LP, lateral plate; BP, basal plate; X-Te, tergum X

***Male*.** [Figs. 2e, 10] Essentially as female, except for following characters: *Head*: antenna verticillate, length 1.03–1.19 (1.13) (*n* = 5); proboscis length 1.13–1.68 (1.45) (*n* = 5); maxillary palpus length 1.25–1.77 (1.51) (*n* = 5); palpomere III with inconspicuous whitish basal ring. *Wing*: length 2.49–2.76 (2.61) (*n* = 5). *Genitalia*: tergum IX lobes slightly globose, apex glabrous, median portion each with 15–20 slender, simple setae; distance between lobes smaller than half basal width of 1 lobe. Gonocoxite oblong; proximal division of subapical lobe with 4 parallel setae (*a*, *b*, *c* and *d*): seta *a* more basal, narrow, with pointed apex; seta *b* long, spatulate, rounded apex, implanted on salient tubercle; seta *c* thin, slender, filiform, slightly curved, inserted between setae *b* and *d*; seta *d* filiform, long, spatulate, implanted on tubercle, with blunt apex. Distal division with short columnar process, with 5 setae: 3 filiform, narrow, pointed, apically inserted, subequal in size setae (seta *f*), 1 filiform seta with hooked apex (seta *h*), and 1 large, long, broad, asymmetrical, ribbed seta arising subapically (seta *l*); 1 saber-like, ribbed seta (seta *s*) arising apically. Gonocoxite with 5 slender, hyaline, short, inconspicuous setae on ventromesal surface; sternomesal surface with long, strong evenly dispersed setae. Gonostylus as in *Cx*. *atratus*, with large gonostylar claw, with slightly rounded apex. Aedeagus with ventral process of lateral plate with numerous spicules; lateral process pointed and directed dorsolaterally. Proctiger with tergum X long, sinuous, somewhat elongated in outline, inner process pointed, narrow and long.

***Pupa*.** [Figs. 3c, 4c] Similar to *Cx*. *atratus* except for followings characters. *Cephalothorax*: setae 1,2-CT 4- or 5-branched; seta 4-CT 3-branched; seta 8-CT 4-branched; seta 11-CT single or double; seta 12-CT double. Trumpet with pinna of median size, irregular in shape, length 0.16–0.23 (0.20) (*n* = 10), distal margin opposite to meatal cleft, which has a large and conspicuous emargination; tracheoid area, darker, extending 0.15–0.23 (0.20) (*n* = 10) from base; trumpet index 12.4–20.0 (14.5) (*n* = 10). *Abdomen*: seta 9-VIII with 2 simple branches; paddle index 1.56–1.95 (1.71) (*n* = 10).

***Larva*.** [Figs. 5c, 6c] In general, similar to *Cx*. *atratus* except for followings characters. *Head*: length 0.64–0.79 (0.74) (*n* = 10), width 1.02–1.16 (1.11) (*n* = 5). Antennal length 0.48–0.57 (0.54) (*n* = 10). Seta 1-A inserted 0.34–0.38 (0.37) (*n* = 10) from antennal base; seta 14-C double, strong. *Abdomen*: comb of segment VIII with 25–35 scales of similar size arranged in 3 or 4 rows. Segment X length 0.31–0.35 (0.34) (*n* = 10), siphon/saddle index 3.36–4.22 (3.87) (*n* = 10). *Siphon*: long, slender, index 6.2–8.9 (7.4) (*n* = 10); pecten with 12 marginal spines as from on basal 0.30. Seta 1-S with 4 ventral pairs and 6 dorsal pairs.

***Bionomics*.** Immature specimens of *Cx*. *dunni* were collected in permanent and semi-permanent partially shaded ground habitats, with slightly turbid water, associated with herbaceous vegetation such as *Pistia* sp. The larvae were collected in remnants of the Atlantic Forest in southeastern Brazil and in transition areas between the Cerrado and the Atlantic Forest biomes, in association with *Cx*. *ensiformis* and *Cx*. *comptus* n. sp.

**Remarks**

*Culex dunni* was described by Dyar [59] from specimens collected in Mandingo River, Canal Zone, Panama. Dyar [36], considered *Cx*. *dunni* to be identical to *Cx*. *ensiformis* of Bonne-Wepster & Bonne based on characteristics of the male genitalia. Bonne & Bonne-Wepster [37] mentioned that Dyar might have examined *Cx*. *dunni* with *Cx*. *ensiformis*, and that *Cx*. *ensiformis* can be distinguished from *Cx*. *dunni* by the crescent-shaped plate at base of gonocoxite and the pattern of scales on the scutum. Dyar & Shannon [60] described *Cx*. *ruffinis* from an adult male from Barro Colorado Island, Canal Zone, Panama. Komp [21] synonymized *Cx*. *ruffinis* with *Cx*. *dunni*, considering a possible misinterpretation of some features of the male genitalia in the original description. In addition, the author hypothesized that the male genitalia of *Cx*. *commevynensis* were similar to those of *Cx*. *dunni*, and that the differences noted by Bonne-Wepster & Bonne [58] were the result of distortions caused during the dissection and mounting process. Senevet & Abonnec [66] attributed the wide distribution observed for *Cx*. *dunni* to a probable confusion with *Cx*. *ensiformis*, considered by them as a morphologically close species. Rozeboom & Komp [6] verified that *Cx*. *commevynensis* possesses “a hair-like” seta on the proximal division of the subapical lobe, while *Cx*. *dunni* has several “spines” in that position, distinguishing the species. Later, Foote [7] described the immature stages of *Cx*. *commevynensis* and suspected that this species was not valid; this author considered *Cx*. *dunni*, *Cx*. *commevynensis* and *Cx*. *zeteki* to be closely related species. Although there has been intense discussion about the taxonomy of *Cx*. *dunni*, this species bears characteristics that clearly differ from the other species of the Atratus Group, especially with regard to features of the male genitalia and of the immature forms. The male genitalia of *Cx*. *dunni* differ from those of *Cx*. *ensiformis* and *Cx*. *commevynensis* in having four parallel setae on the proximal division of the subapical lobe while the other species have only two setae. *Culex dunni* has a long seta *l* in the distal division of the subapical lobe and *Cx*. *ensiformis* has a short seta. *Culex dunni* also differs in possessing several spicules on the ventral process of the lateral plate and in having tergum X appearing long, sinuous and elongated in outline while *Cx*. *ensiformis* and *Cx*. *commevynensis* have a lateral plate without spicules and a shorter tergum X. Based on larval characteristics, *Cx*. *dunni* differs from *Cx*. *ensiformis* in having subequal comb scales; double and strong seta 14-C, and short pecten spines with a conspicuously serrate border. Additionally, *Cx*. *dunni* differs from *Cx*. *ensiformis* and the other species in having a pinna of median size and a conspicuous emargination on the distal margin opposite the meatal cleft. With respect to adult specimens, both *Cx*. *dunni* and *Cx*. *ensiformis* bear a patch of pale scales separated by dark scales on the base of vein R and occasionally a small pale patch on the base of vein C. However, *Cx*. *dunni* has bronze scales on the scutum, not forming a pattern and in the male, an inconspicuous whitish basal ring on palpomere III, different to what is observed in *Cx*. *ensiformis*, which possesses scutal scales with different colour that form a pattern and palpomere III of the male dark-scaled.

***Culex*(*Melanoconion*)*ensiformis*Bonne-Wepster & Bonne, 1919**

1919: *Culex* (*Melanoconion*) *ensiformis* Bonne-Wepster & Bonne, 1919: 176 [58] (♂, ♀, ♂G, L). Paratypes ♂, ♂G, ♀ deposited in the USNM. Type locality: Dam, Suriname.

*Culex* (*Melanoconion*) *ensiformis* of Dyar (1923: 188) [36] (synonymy with *Cx*. *dunni*); Bonne & Wepster-Bonne (1925: 272) [37] (resurrected from synonymy, ♂, ♀, ♂G, L); Senevet & Abonnenc (1939: 81) [67] (♂G); Rozeboom & Komp (1950: 98) [6] (synonymy with *Cx*. *zeteki*); Foote (1954: 97) [7]; Belkin (1968: 15) [39] (resurrected from synonymy with *Cx*. *zeteki*, lectotype desig.); Pecor et al. (1992: 25) [15] (distr.); Pecor et al. (2002: 247) [63] (Belize, L, P); Hutchings et al. (2011) [70] (Brazil); Torres-Gutierrez & Sallum (2015: 18) [10] (distr.).

***Type material*:** Paratypes, pinned adult male with associated larval and pupal exuviae on slide, in poor condition (USNM no. 22709-BB638) and pinned adult female with associated larval and pupal exuviae on slide (USNM no. 22709-BB625) deposited in the Diptera Collection, National Museum of Natural History (USNM), Washington, DC, USA.

***Material examined*:** 155 specimens: 77♂G, 98Le, 143Pe, 7♂, 5♀. FSP-USP, Brazil: São Paulo State, Cananéia Municipality, Vilarinho (− 24.951551, − 47.977989), coll. Sá et al. 14.vii.2015, det. Sá 2015: SP166-02 (Le, Pe, ♂G); SP166-07 (Pe, ♂G); SP166-08 (Le, Pe, ♂G); SP166-09 (Le, Pe); SP166-11 (Le, Pe); SP166-12 (Le, Pe); SP166-14 (Le, Pe); SP166-15 (Le, Pe); SP166-16 (Le, Pe); SP166-17 (Le, Pe, ♂G); SP166-18 (Le, Pe); SP166-19 (Le, Pe, ♂G); SP166-20 (Le, Pe, ♂G); SP166-22 (Le, Pe); SP166-24 (Le, Pe); SP166-25 (Le, Pe); SP166-26 (Le, Pe); SP166-27 (Le, Pe); SP166-28 (Le, Pe); SP166-33 (Le, Pe, ♂G); SP166-34 (Le, Pe, ♂G); SP166-35 (Le, Pe, ♂G); SP166-37 (Le, Pe, ♂G); SP166-38 (Le, Pe); SP166-39 (Le, Pe); SP166-40 (Le, Pe, ♂G); SP166-41 (Le, Pe); SP166-42 (Le, Pe); SP166-44 (Le, Pe); SP166-46 (Le, Pe); SP166-47 (Le, Pe, ♂G); SP166-48 (Le, Pe, ♂G); SP166-49 (Le, Pe, ♂G); SP166-52 (Le, Pe, ♂G); SP166-53 (Le, Pe); SP166-55 (Le, Pe); SP166-56 (Le, Pe, ♂G); SP166-57 (Le, Pe, ♂G); SP166-58 (Le, Pe); SP166-59 (Le, Pe); SP166-60 (Le, Pe, ♂G); SP166-62 (Le, Pe); SP166-65 (Le, Pe, ♂G); SP166-66 (Le, Pe); SP166-68 (Le, Pe, ♂G); SP166-69 (Le, Pe); SP166-70 (Le, Pe); SP166-73 (Le, Pe); SP166-75 (Le, Pe); SP166-76 (Le, Pe, ♂G); SP166-77 (Le, Pe, ♂G); SP166-78 (Le, Pe); SP166-79 (Le, Pe, ♂G); SP166-80 (Le, Pe); SP166-81 (Le, Pe); SP166-82 (Le, Pe, ♂G); SP166-88 (Le, Pe); SP166-89 (Le, Pe, ♂G); SP166-91 (Le, Pe); SP166-93 (Le, Pe, ♂G); SP166-94 (Le, Pe, ♂G); SP166-96 (Le, Pe, ♂G); SP166-97 (Le, Pe); SP166-99 (Le, Pe); SP166-100 (Pe); SP166-101 (Pe); SP166-102 (Pe, ♂G); SP166-103 (Pe); SP166-105 (Pe, ♂G); SP166-111 (Pe); SP166-112 (Pe); SP166-113 (Pe, ♂G); SP166-115A (Pe); SP166-115B (Pe); SP166-117 (Pe, ♂G); SP166-118 (Pe); SP166-119 (Pe, ♂G); SP166-120 (Pe); SP166-121 (Pe, ♂G); SP166-301 (Le, Pe); SP166-304 (Le, Pe); SP166-305 (Le, Pe); SP166-311 (Le, Pe); SP166-313 (Le, Pe). São Paulo State, Cananéia Municipality, Folha Larga Farm (− 24.89273, − 47.919048), coll. Sá et al. 14.vii.2015, det. Sá 2015: SP167-07 (Le, Pe); SP167-09 (Le, Pe); SP167-10 (Le, Pe, ♂G); SP167-13 (Le, Pe); SP167-16 (Le, Pe); SP167-17 (Le, Pe, ♂G); SP167-18 (Le, Pe, ♂G); SP167-20 (Le, Pe, ♂G); SP167-21 (Le, Pe, ♂G); SP167-24 (Le, Pe, ♂G); SP167-25 (Le, Pe, ♂G); SP167-32 (Le, Pe, ♂G); SP167-33 (Le, Pe, ♂G); SP167-39 (Le, Pe); SP167-124 (Pe, ♂G); SP167-127 (Pe, ♂G); SP167-129 (Pe, ♂G); SP167-133 (Pe, ♂G); SP167-134 (Pe). São Paulo State, Cananéia Municipality, Vilarinho (− 24.951551, − 47.977989), coll. Sá et al. 12.ii.2016, det. Sá 2016: SP171-07 (Pe, ♂G); SP171-08 (Le, Pe); SP171-09 (Le, Pe); SP171-10 (Le, Pe, ♂G); SP171-11 (Le, Pe); SP171-12B (Le, Pe, ♂G); SP171-111 (Pe, ♂G); SP171-118 (Pe); SP171-119 (Pe, ♂G); SP171-143 (Pe, ♂G); SP171-144 (Pe); SP171-146 (Pe); SP171-147 (Pe). B.M.M-9 (Le, Pe). São Paulo State, Cananéia Municipality, Itapitangui (− 24.935105, − 47.961728), coll. Forattini et al. 1983, det. Sallum 1983: HEP81-04 (Pe, ♂G); HEP81-05 (Pe); HEP 81-06 (Pe, ♀); HEP81-07 (Pe); HEP81-08 (Pe, G♂, ♂); HEP81-11 (Pe, ♂G); HEP81-12 (Pe); HEP81-13 (Pe, ♀); HEP81-14 (Pe, ♂G); HEP81-15 (Pe, G♂, ♂); HEP81-16 (Le, Pe, ♀); HEP81-18 (Le, Pe, ♂G); HEP81-19 (Le, Pe); HEP81-20 (Le, Pe); HEP81-21 (Le, Pe, ♂G); HEP81-23 (Le, Pe, G♂, ♂); HEP81-24 (Pe, ♂G); HEP81-25 (Le, Pe, ♀). São Paulo State, Cananéia Municipality, Itapitangui (− 24.935105, − 47.961728), coll. Forattini et al. 1983, det. Sallum 1983: HEP81-10 (Pe, ♂G); HEP81-31 (Le, Pe, ♂G, ♂); HEP81-33 (Le, Pe, ♀). São Paulo State, Cananéia Municipality, estrada de Cananéia (− 24.995729, − 47.930414), coll. Forattini et al. 12.ix.1984, det. Sallum 1984: HEP434-04 (Le, Pe, ♂G). Amazonas State, Humaitá Municipality, Realidade, (− 7.106919, − 63.11572), coll. Chaves et al. 22.vii.2016, det. Sá 2016: Coleta07-Humaitá-01 (♂G, ♂); Coleta07-Humaitá-02 (♂G, ♂). INPA, Brazil: Amazonas State, Manaus Municipality, Acampamento Colosso, Fazenda Esteio (− 2.40417, − 59.86361), coll. Hutchings et al. 2002, det. Sallum & Hutchings: Fam-000631 (♂, G♂); Fam-000632 (♂G); Fam-002314 (♂G); Fam-002925 (♂G); Fam-002927 (♂G); Fam-002932 (♂G). Amazonas State, Manaus Municipality, Fazenda Esteio (− 2.45278, − 59.75278); coll. Hutchings et al. 2003, det. Sallum & Hutchings: Fam-004667 (♂G). Amazonas, Manaus, Fazenda Esteio (− 2,4278, − 59.7528), coll. Hutchings et al. 2002, det. Sallum & Hutchings: Fam-003224 (♂G). Amazonas State, Manaus Municipality, Fazenda Porto Alegre, BR-174 (− 2.38139, − 59.94222), coll. Hutchings et al. 2002, det. Sallum & Hutchings: Fam-003557 (♂G). Amazonas State, Manaus Municipality, Fazenda Porto Alegre, BR-174 (− 2.355, − 59.9575), coll. Hutchings et al. 2002, det. Sallum & Hutchings: Fam-002343 (♂G). USNM, Brazil: Pará State, Altamira Municipality, km 158, coll. Reinert et al. 9.xi.1974: 446Coll- 111-109 (Pe ♂); 111-133 (Pe ♀); 111-107 (Pe ♀); 111-129 (Pe ♂).

***Distribution*:***Culex ensiformis* has been collected in Belize [63], Bolivia [71], Brazil [70], French Guiana [67] and Suriname. In Brazil, the species was collected in the municipalities of Altamira, Pará State, Manaus and Humaitá in Amazonas State, and in the municipalities of Cananéia, Pariquera-Açu and Presidente Epitácio, São Paulo State (present study).

**Description**

***Female*.***Head*: antenna dark, flagellum normal, whorls with 5 setae, length 1.25–1.74 (1.49) (*n* = 5); proboscis dark-scaled, length 1.16–1.52 (1.40) (*n* = 5); maxillary palpus with dark scales, length 0.24–0.31 (0.27) (*n* = 5). Occiput with dark brown erect forked scales. *Thorax*: scutum covered with narrow, bronze, and golden falcate scales on acrostichal and dorsocentral areas; occasionally whitish scales on anterior promontory, supraalar, and prescutellar areas forming a pattern. Scutellar scales whitish; median lobe with 5 or 6 dark setae; lateral lobes each with 3 or 4 setae. Pleural setae with 2 types of colouring: brown with bronzy reflections: 8 or 9 antepronotal, 4 or 5 prealar; and pleural setae golden, hyaline: 3–5 upper mesokatepisternal, 3 or 4 lower mesokatepisternal, 4 or 5 upper mesepimeral and 1 large lower mesepimeral. Pleura with large patch of broad, white scales on upper mesokatepisternum; lower mesokatepisternum with few white scales. *Wing*: dark-scaled, vein R with 2 proximal patches of white scales separated by small patch of dark scales; vein C with proximal patch of white scales; wing length 2.58–3.38 (3.06) (*n* = 5). *Halter*: scabellum, pedicel and capitellum brownish. *Legs*: as in *Cx*. *atratus*. *Abdomen*: tergum I with dark scales; terga III-VII dark-scaled with basal bands of white scales; tergum VIII dark-scaled.

**Fig. 11:**
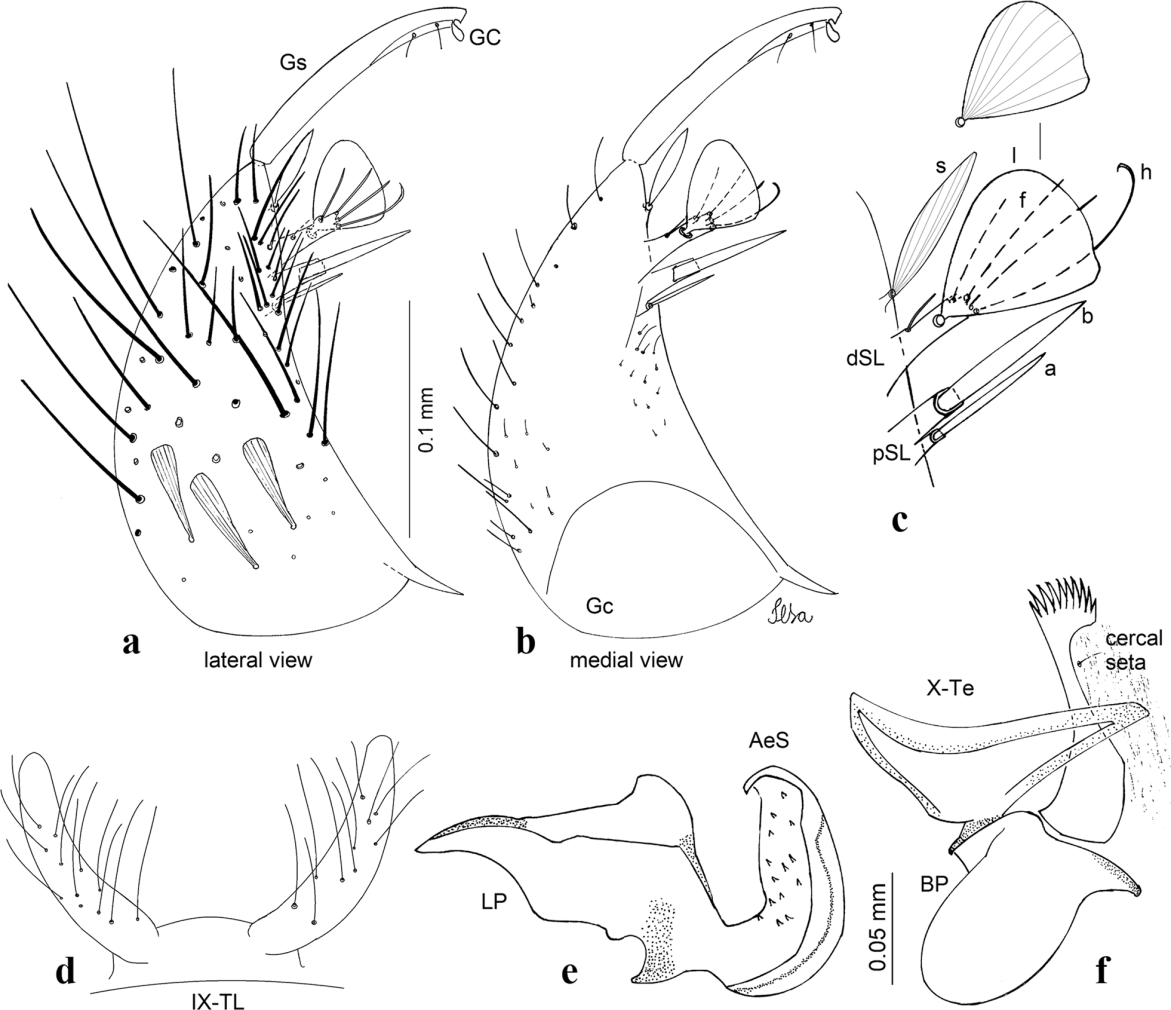
*Culex* (*Melanoconion*) *ensiformis*, male genitalia. **a** Gonocoxite in lateral view. **b** Gonocoxite in medial view. **c** Setae on the subapical lobe of the gonocoxite. **d** Tergum IX lobe. **e** Aedeagus. **f** Proctiger. *Abbreviations*: Gc, gonocoxite; Gs, gonostylus; GC, gonostylar claw; dSL, distal division of subapical lobe; pSL, proximal division of subapical lobe; IX-TL, tergum IX lobe; AeS, aedeagal sclerite; LP, lateral plate; BP, basal plate; X-Te, tergum X

***Male*.** [Figs. 2f, 11] Similar to female, except for following characters: *Head*: antenna verticillate, length 1.05–1.63 (1.36) (*n* = 5); proboscis length 1.39–1.93 (1.65) (*n* = 5); maxillary palpus length 1.71–2.15 (1.99) (*n* = 5). *Wing*: length 2.69–3.16 (2.88) (*n* = 5). *Genitalia*: tergum IX lobes conical, elongate, slender, with apex glabrous, median portion each with 14–20 slender, simple setae; distance between lobes equivalent to basal width of 1 lobe. Gonocoxite oblong; proximal division of subapical lobe with 2 parallel setae (*a* and *b*): seta *a* more basal, narrow, slender, with pointed apex; seta *b* long, spatulate, with pointed apex, implanted on salient tubercle. Distal division with elongate columnar process, with 5 setae: 3 filiform, narrow, pointed, inserted apically, subequal in size (seta *f*), 1 filiform seta with hooked apex (seta *h*), and 1 short, broad, asymmetrical, ribbed seta arising subapically (seta *l*); 1 saber-like, ribbed seta (seta *s*) arising apically. Additionally, 1 small, hyaline, inconspicuous seta basally on columnar process. Gonocoxite with 3 or 4 slender, hyaline, short, inconspicuous setae on ventromesal surface. Gonostylus as *Cx*. *atratus*, with large gonostylar claw, with slightly pointed apex. Aedeagus with ventral process of lateral plate with small convexity; aedeagal sclerite with spicules on ventral surface. Proctiger with tergum X narrow, somewhat triangular in outline, with slightly pointed inner process.

***Pupa*.** [Figs. 3d, 4d] *Cephalothorax*: setae 1,2-CT 4- or 5-branched; seta 3-CT double; seta 4-CT 5-branched; seta 5-CT 3-branched; seta 6,7-CT 3-branched; seta 8-CT 7-branched; seta 9-CT 3-branched; seta 10-CT 6-branched; seta 11-CT double; seta 12-CT 4-branched. Trumpet long, slender, with dilated apex; pinna small, opening circular, pinna length 0.07–0.13 (0.11) (*n* = 10), distal margin opposite meatal cleft with shallow depression; tracheoid area extending 0.20–0.34 (0.29) (*n* = 10) from base; trumpet index 16.2–29.7 (24.2) (*n* = 10). *Abdomen*: seta 9-VIII with 2 simple branches; paddle index 1.49–1.95 (1.68) (*n* = 10).

***Larva*.** [Figs. 5d, 6d] *Head*: length 0.70–0.81 (0.79) (*n* = 10), width 1.08–1.20 (1.13) (*n* = 10). Antennal length 0.62–0.70 (0.67) (*n* = 10); seta 1-A inserted 0.42–0.46 (0.45) (*n* = 10) from antennal base. Seta 5-C with 8 long branches; seta 11-C 3-branched; seta 13-C double. *Abdomen*: comb of segment VIII with 16–22 scales of different sizes arranged in 2 or 3 rows: upper rows with small, pointed scales; lower row with 5–9 large, pointed scales. Segment X length 0.32–0.39 (0.36) (*n* = 10), siphon/saddle index 4.26–4.95 (4.51) (*n* = 10). *Siphon*: long, slender, index 7.6–10.4 (9.2) (*n* = 10); pecten with 10 spines on basal 0.30 of siphon. Seta 1-S usually with 4 ventral pairs and 4 dorsal pairs.

***Bionomics*.** Immature specimens of *Cx*. *ensiformis* were collected in semipermanent partially shaded groundwater habitats with herbaceous vegetation such as *Pistia* sp. in remnants of the Atlantic Forest in association with *Cx*. *dunni*. Adults were collected in the Amazon Forest.

**Remarks**

*Culex ensiformis* was described by Bonne-Wepster & Bonne [58] from adults and larvae collected in Suriname. Dyar [36] synonymized this species with *Cx*. *dunni*. Bonne & Wepster-Bonne [37] resurrected it from synonymy with *Cx*. *dunni*. According to Bonne & Wepster-Bonne [37], Dyar may have examined *Cx*. *dunni* and *Cx*. *ensiformis* in the same material, because *Cx*. *ensiformis* possesses morphological differences, such as the crescent-shaped lateral plate and the scale pattern of the scutum, that can distinguish it from *Cx*. *dunni*. Senevet & Abonnec [66] considered *Cx*. *ensiformis* to be close to but distinct from *Cx*. *dunni*, and resurrected it from synonymy again. Rozeboom & Komp [6] compared *Cx*. *ensiformis* with *Cx*. *zeteki* and considered the former to be a synonym of *Cx*. *zeteki* based on features of the male genitalia and the color pattern of the scales on the scutum. Likewise, Foote [7] maintained *Cx*. *ensiformis* in synonymy with *Cx*. *zeteki* based on the presence of two types of comb scales in the larva. Belkin [39] considered *Cx*. *ensiformis* as a distinct species close to *Cx*. *commevynensis* but not conspecific with *Cx*. *zeteki*, and designated a lectotype male associated with larval and pupal exuviae while resurrecting *Cx*. *ensiformis* (see taxonomic discussion for *Cx*. *dunni* for additional information). Pecor et al. [63] provided some morphological characteristics to distinguish *Cx*. *ensiformis* and *Cx*. *commevynensis* based on the morphology of the pupal stage. According to these authors, *Cx*. *ensiformis* is most readily distinguished from *Cx*. *commevynensis* and the other species belonging to Atratus Group in the pupal stage, because it bears morphological characteristics markedly unique to this species, as follows: trumpet distinctly flared at the apex and a trumpet index greater than 10. *Culex zeteki* has a trumpet with a smaller pinna than in *Cx*. *ensiformis*. *Culex dunni* has a trumpet with a larger pinna than in *Cx*. *ensiformis* and with a conspicuous emargination. *Culex commevynensis* has a straight pinna which is not flared apically as in *Cx*. *ensiformis*. Regarding the larval stage, *Cx*. *ensiformis* can be distinguished from the other species belonging to the group in having seta 5-C with 5 or more long branches (usually with 8 branches), and comb scales of two different sizes in 2 or 3 rows (the upper rows with small scales and the lower rows with fewer, larger scales). Comb scales of uneven sizes can also be found in *Cx*. *trigeminatus*; however, *Cx*. *ensiformis* differs from *Cx*. *trigeminatus* in having a few large scales and in having seta 5-C with long branches reaching the base of seta 6-C. Adults of *Cx*. *ensiformis* can be distinguished from the other species in possessing vein R with two proximal patches of white scales separated by small patch of dark scales, vein C with a proximal patch of white scales and the scutum with a pattern of whitish, bronze and golden scales, whereas *Cx*. *zeteki* has entirely dark-scaled wings, *Cx. trigeminatus* has a single large patch of whitish scales on vein R, *Cx*. *dunni* has two short patches of whitish scales on vein R and *Cx*. *comptus* n. sp. has dark and whitish scales on the scutum. Regarding male genitalia, *Cx*. *ensiformis* can be readily distinguished from *Cx*. *dunni*, *Cx*. *zeteki* and *Cx*. *trigeminatus* in having two parallel spatulate setae on the proximal division of the subapical lobe and conspicuous spicules on the ventral surface of the aedeagal sclerite. *Culex ensiformis* differs from *Cx*. *comptus* n. sp. in having a short columnar process and a short seta *l* on the distal division of the subapical lobe.

***Culex*(*Melanoconion*)*exedrus*Root, 1927**

1927: *Culex* (*Melanoconion*) *exedrus* Root, 1927: 580 [72] (♂, ♀, ♂G) lectotype ♂, ♂G deposited in the USNM. Type locality: Porto das Caixas, Rio de Janeiro, Brazil.

*Culex* (*Melanoconion*) *exedrus* of Dyar (1928: 341) [19] (synonymy with *Cx*. *ruffinis*); Rozeboom & Komp (1950: 89) [6] (synonymy with *Cx*. *dunni*), Stone & Knight (1957: 49) [45] (desig. lectotype).

***Type material*:** Lectotype, pinned adult male (USNM no. 40529), in poor condition, with dissected genitalia on slide (USNM no. 30-1) deposited in the Diptera Collection, National Museum of Natural History (USNM), Washington, DC, USA.

***Distribution*:***Culex exedrus* has been collected in the Porto das Caixas and Paracambi municipalities, Rio de Janeiro State, Brazil [72].

**Description**

**Fig. 12:**
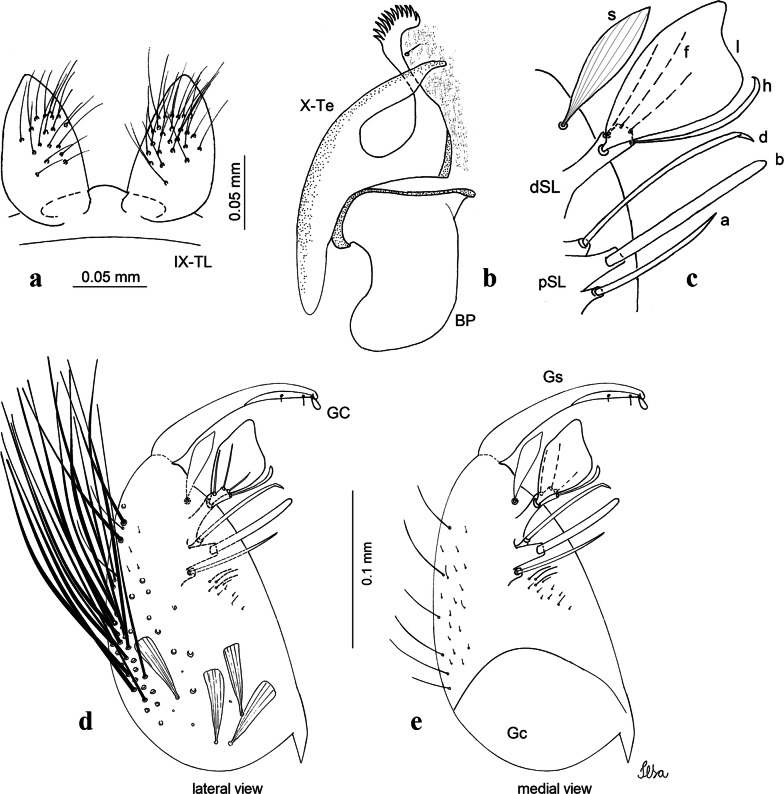
*Culex* (*Melanoconion*) *exedrus*, male genitalia. **a** Tergum IX lobe. **b** Proctiger. **c** Setae on the subapical lobe of the gonocoxite. **d** Gonocoxite in lateral view. **e** Gonocoxite in medial view. *Abbreviations*: Gc, gonocoxite; Gs, gonostylus; GC, gonostylar claw; dSL, distal division of subapical lobe; pSL, proximal division of subapical lobe; IX-TL, tergum IX lobe; BP, basal plate; X-Te, tergum X

***Male*.** [Fig. 12] Essentially similar to *Cx*. *dunni*, except as follows: *Genitalia*: gonocoxite with long, strong setae, aligned from base to apex on sternomesal surface. Proximal division of subapical lobe with 3 parallel setae (*a*, *b* and *c*): seta *a* inserted basally, narrow; seta *b* long, spatulate, borne on salient tubercle; seta *c* filiform, long, spatulate, borne on small tubercle, apex blunt; and 1 saber-like, ribbed seta (seta *s*) with broad apex, arising apically. Proctiger with tergum X long, sinuous, somewhat elongate in outline, inner process pointed, long and wide.

***Female*.** Not examined.

***Pupa and larva*.** Unknown.

***Bionomics*.** Immatures of *Cx*. *exedrus* were collected in ground water sites such as river margins, lagoons and ponds, associated with thick aquatic vegetation [72].

**Remarks**

*Culex exedrus* was described by Root [72] based on male and female specimens from Porto das Caixas and Paracambi, Rio de Janeiro State, Brazil. Dyar [19] synonymized *Cx*. *exedrus* with *Cx*. *ruffinis* Dyar & Shannon [60]. Rozeboom & Komp [6] considered *Cx*. *exedrus* as a junior synonym of *Cx*. *dunni* and maintained this taxonomic status until *Cx*. *exedrus* was resurrected from synonymy. *Culex exedrus* can be distinguished from *Cx*. *dunni* by features of the male genitalia, mainly a large number of long setae visibly lined up on the sternomesal surface of the gonocoxite; additionally, *Cx*. *exedrus* has seta *s* of the gonocoxite with a wider apex and a proctiger with the inner process of tergum X wider than in *Cx*. *dunni*.

***Culex*(*Melanoconion*)*longisetosus*Sá & Sallum n. sp.**

***Type locality*:** Pariquera-Açu Municipality (− 47.88083, − 24.7150), São Paulo State, Brazil. Adults were collected in the Atlantic Forest and in seasonally flooded *várzea* forests along the Amazonas and Solimões Rivers.

***Other localities*:** Pariquera-Açu Municipality, São Paulo State; Santo Antônio do Içá, Jutaí, Coari and Itacoatiara municipalities, Amazonas State; and, in Juruti and Almeirim municipalities, Pará State, Brazil.

***Type material*:** Holotype, pinned adult male with associated dissected genitalia on slide (specimen field no. 543, accession no. FSP-USP E-15891), with following collection data: Brazil, São Paulo State, Pariquera-Açu Municipality (− 47.88083, − 24.7150), coll. Forattini et al., 11.i.1979, with Shannon trap, deposited in the Coleção Entomológica de Referência, Faculdade de Saúde Pública, Universidade de São Paulo (FSP-USP), São Paulo, São Paulo State, Brazil. Paratypes: 7 pinned adult males with associated dissected genitalia on slide from same collection as holotype and deposited in FSP-USP: specimen field no. 2661, accession no. FSP-USP E-15900 (coll. 10.vi.1980), specimen field no. 2695, accession no. FSP-USP E-15892 (coll. 17.vi.1980), specimen field no. 2756, accession no. FSP-USP E-15893 (coll. 11.xii.1980), specimen field no. 02, accession no. FSP-USP E-15894 (coll. 9.ii.1981), specimen field no. 2173, accession no. FSP-USP E-15895 (coll. 10.ii.1981), specimen field no. 01, accession no. FSP-USP E-15896 (coll. 29.i.1981), and specimen field no. 2967, accession no. FSP-USP E-15897 (coll. 12.iii.1981); 1 pinned adult male with associated dissected genitalia on slide (specimen field no. 3582, accession no. FSP-USP E-15898), with following collection data: Brazil, São Paulo State, Pariquera-Açu Municipality, Experimental Farm, 7.v.1984; 1 pinned adult male with associated dissected genitalia on slide (specimen field no. 4048, accession no. FSP-USP E-15899), with following collection data: Brazil, São Paulo State, Pariquera-Açu Municipality, Pariquera-Mirim district (− 24.729867, − 47.813300) on 2-II-1985, both deposited in the FSP-USP; and 5 pinned adult males with associated dissected genitalia on separate slides, from different locations: specimen field no. ProV-053607, accession no. INPA-DIP 004574, with following collection data: Brazil, Pará State, Juruti, Recreio, Parana de Dona Rosa, Amazon River (− 2.07554, − 55.96586), coll. Hutchings et al. 30-31.x.2003, det. Sallum & Hutchings 2016; specimen field no. ProV-047936, accession no. INPA-DIP 004575, with following collection data: Brazil, Pará State, Almeirim Municipality, Arumanduba, Amazon River (− 1.48631, − 52.48706), coll. Hutchings et al. 19-20.x.2003, det Sallum, Hutchings & Sá 2017; specimen field no. ProV-005165, accession no. INPA-DIP 004576, with following collection data: Brazil, Amazonas State, Santo Antônio do Içá Municipality, Parana do Canini, Solimões River (− 3.15123, − 68.00142), coll. Hutchings et al. 15-16.ix.2003, det. Hutchings & Sá 2017; specimen field no. ProV-044118, accession no. INPA-DIP 004577, with following collection data: Brazil, Amazonas State, Coari Municipality, Ilha do Botija, Trocaris, Solimões River (− 3.91375, − 62.84982), coll. Hutchings et al. 25-26.ix.2003, det. Hutchings & Sá 2017; specimen field no. ProV-057487, accession no. INPA-DIP 004573) with following collection data: Brazil, Amazonas State, Itacoatiara Municipality, São Jorge, Parana da Eva, Amazon River (− 3.15751, − 59.32323), coll. Hutchings et al. 7-8.ix.2003, det. Hutchings & Sá 2017, all deposited in the Coleção de Invertebrados, Instituto Nacional de Pesquisas da Amazônia (INPA), Manaus, Amazonas State, Brazil.

***Material examined*:** 3 G♂, 3 ♂. INPA, Brazil: Pará State, Juruti Municipality, Recreio, Parana de Dona Rosa, Amazon River (− 2.07554, − 55.96586), coll. Hutchings et al. 30–31.x.2003, det. Sallum & Hutchings 2016: ProV-053597 (♂G). Pará State, Almeirim Municipality, Arumanduba, Amazon River (− 1.48631, − 52.48706), coll. Hutchings et al. 19–20.x.2003, det. Sallum, Hutchings & Sá 2017: ProV-047940 (♂G). Amazonas State, Jutaí Municipality, São Raimundo, Parana do Cervalho, Solimões River (− 2.70907, − 66.89931), coll. Hutchings et al. 16-17.ix.2003, det. Hutchings & Sá 2017: ProV-007278 (♂G).

***ZooBank registration*:** The Life Science Identifier (LSID) for *Culex* (*Melanoconion*) *longisetosus* n. sp. is urn:lsid:zoobank.org:act: 2F0C0B21-08FE-458E-94D3-EB8D2550B5C8.

***Etymology*:** The name *longisetosus* is derived from a combination of the Latin noun *saeta*, meaning “seta, bristle” and with the Latin adjective *lātus*, meaning “extensive, broad”. *Culex longisetosus* is named in reference to the four long and spatulate setae borne ventromesally between the proximal and distal divisions of the subapical lobe of the male genitalia.

**Description**

**Fig. 13:**
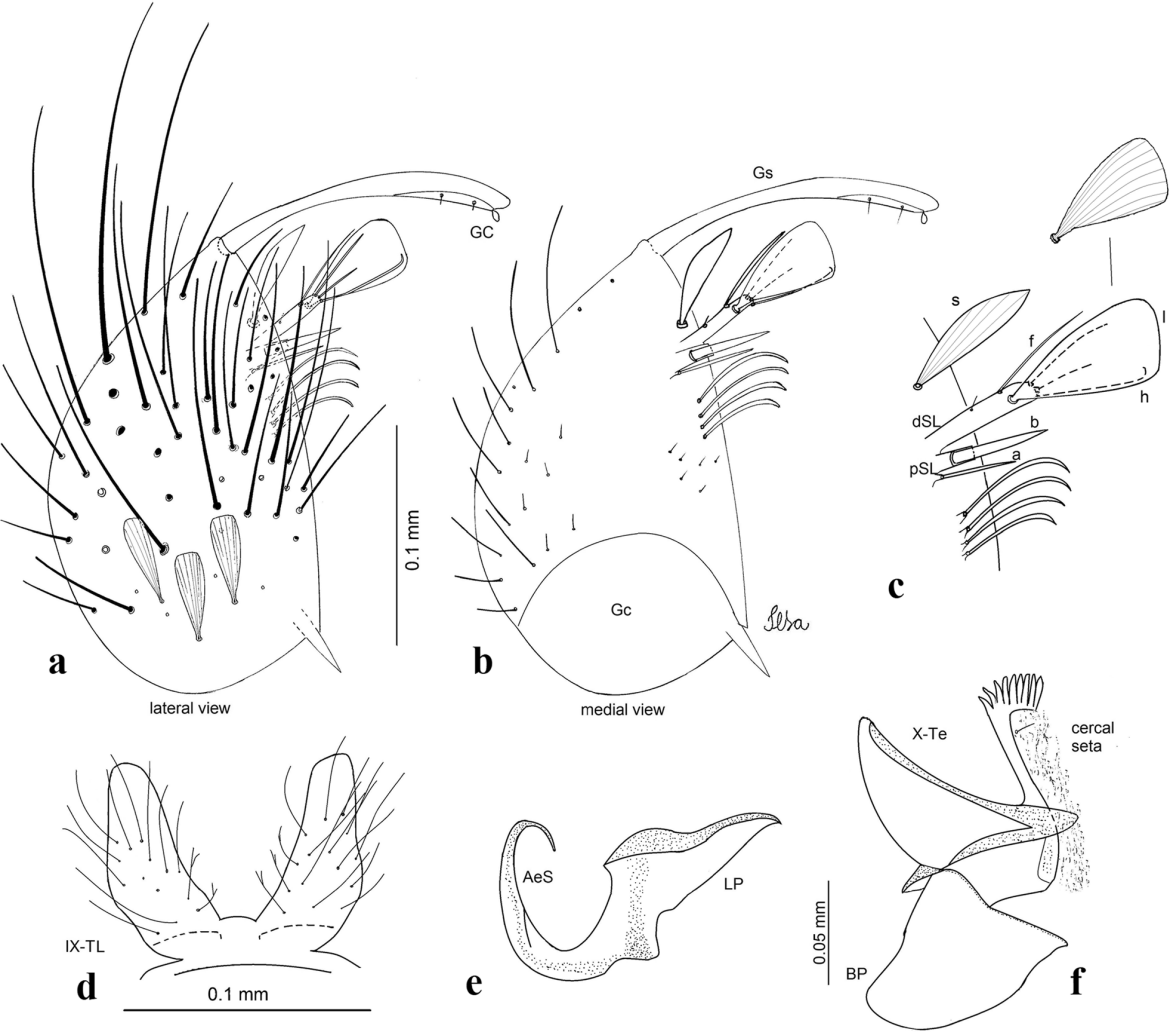
*Culex* (*Melanoconion*) *longisetosus* n. sp., male genitalia. **a** Gonocoxite in lateral view. **b** Gonocoxite in medial view. **c** Setae on the subapical lobe of the gonocoxite. **d** Tergum IX lobe. **e** Aedeagus. **f** Proctiger. *Abbreviations*: Gc, gonocoxite; Gs, gonostylus; GC, gonostylar claw; dSL, distal division of subapical lobe; pSL, proximal division of subapical lobe; IX-TL, tergum IX lobe; AeS, aedeagal sclerite; LP, lateral plate; BP, basal plate; X-Te, tergum X

***Male*.** [Figs. 2g, 13] *Head*: antennal length 1.02–1.71 (1.25) (*n* = 5); proboscis entirely dark-scaled, length 1.04–1.65 (1.43) (*n* = 5); maxillary palpus dark-scaled, length 1.32–1.98 (1.71) (*n* = 4); palpomere II with small, basal patch of whitish scales; palpomere III with inconspicuous proximal patch of whitish scales; palpomeres IV and V dark-scaled, with long, strong setae. Occiput with dark brown, erect forked scales. *Thorax*: scutum covered with narrow, dark brown falcate scales, except anterior promontory and prescutellar area with whitish scales. Median scutellar lobe with 6 dark large setae; lateral lobes each with 4 setae. Pleural setae with 2 types of colouring: dark brown: 3–5 antepronotal, 4 or 5 prealar; and pleural pale golden, slender setae: 4 upper mesokatepisternal, 5 lower mesokatepisternal, 4 or 5 upper mesepimeral; lower mesepimeron with 1 long, strong seta. Pleura with distinct patch of broad, white scales on upper mesokatepisternum; lower mesokatepisternum with few white scales. *Wing*: dark-scaled; length 2.37–2.49 (2.45) (*n* = 5). *Halter*: scabellum and pedicel whitish, capitellum whitish with few brown scales. *Legs*: coxae pale; ventral surface of fore- and midfemur with longitudinal stripe of white scales; tibiae dark-scaled; joints of femur-tibia and tibia-tarsomere I with ring of pale scales; tarsi entirely dark-scaled. *Abdomen*: tergum I with dark scales; terga III-VII dark-scaled, with white basal bands. *Genitalia*: tergum IX lobes elongate, each with 12–14 slender, apically bifid, and simple setae in median portion; apex glabrous; distance between lobes less than basal width of 1 lobe. Gonocoxite oblong, narrow, small; subapical lobe divided into 2 columnar divisions; proximal division with 2 pointed setae (*a* and *b*); seta *a* shorter, slender, inserted basal to seta *b*; seta *b* spatulate, robust; gonocoxite with 4 long, spatulate setae on ventromesal surface; distal division with long columnar process, with 5 setae: 3 filiform, narrow, pointed, different in size (seta *f*), 1 long seta with hooked apex (seta *h*), and 1 large, broad, asymmetrical seta arising subapically (seta *l*); 1 saberlike seta (seta *s*) arising apically. Gonostylus with apex moderately rounded, short leaf-like gonostylar claw borne apically. Aedeagus with ventral process with small convexity.

**Remarks**

Adults of *Cx*. *longisetosus* n. sp. differ from *Cx*. *atratus* in possessing an inconspicuous basal patch of whitish scales on palpomere III and a small basal patch of whitish scales on palpomere II. The male genitalia of *Cx*. *longisetosus* n. sp. can be distinguished from those of other species of the Atratus Group in having a long columnar process in the distal division, elongate and slightly widened from the base to the apex of the ninth tergal lobe, and 4 long, spatulate setae on the ventromesal surface of the gonocoxite.

***Culex*(*Melanoconion*)*longistylus*Sá & Sallum n. sp.**

***Type locality*:** Dourado Municipality (− 22.100000, − 48.317778), São Paulo State, Brazil. Adults were collected in transitional vegetation areas between the Atlantic Forest and Cerrado biomes, and in seasonally flooded *várzea* forest areas along the Amazon River.

***Other localities*:** Dourado and Presidente Epitácio municipalities, São Paulo State; Bataguassu Municipality, Mato Grosso do Sul State; Senador Guiomard Municipality, Acre State; Itacoatiara Municipality, Amazonas State; Almeirim, Prainha, Obidos, Santarém and Juruti municipalities, Pará State.

***Type material*:** Holotype male pinned with associated dissected genitalia on slide (specimen field no. 126, accession no. FSP-USP E-15889), with following collection data: Brazil, São Paulo State, Dourado Municipality (− 22.100000, − 48.317778), coll. Forattini et al. 2.ix.1980, with CDC light trap at the edge of the forest, deposited in the Coleção Entomológica de Referência, Faculdade de Saúde Pública, Universidade de São Paulo (FSP-USP), São Paulo, São Paulo State, Brazil. Paratypes: 1 pinned adult male with dissected genitalia on slide (specimen field no. 163, accession no. FSP-USP E-15890), from the same collection as the holotype and deposited in the same institution (FSP-USP); 1 pinned adult male with dissected genitalia on slide (specimen field no. ProV-050195, accession no. INPA-DIP 004568), with following collection data: Brazil, Pará State, Santarém Municipality, Parana de Ituqui, Amazon River (− 2.47233, − 54.31594), coll. Hutchings et al. 25–26.x.2003, det. Hutchings 2015; 2 pinned adult males with dissected genitalia on separate slides (specimen field no. rBIA-000463, accession no. INPA-DIP 004569 and specimen field no. rBIA000464, accession no. INPA-DIP 004570), with following collection data: Brazil, Acre State, Senador Guiomard Municipality, Fazenda Experimental Catuaba, UFAC, BR-364 km 23 (− 10.05739, − 67.60013), coll. Hutchings & Carmo 23–24.viii.2016, Sá 3.iii.2017; 2 pinned adult males with dissected genitalia on separate slides (specimen field no. ProV-055376, accession no. INPA-DIP 004571 and specimen field no. ProV-055464, accession no. INPA-DIP 004572), with following collection data: Brazil, Pará State, Almeirim Municipality, Paraíso, Paranaquara, Amazon River (− 1.74512, − 53.154), coll. Hutchings et al. 21–22.x.2003, det. Hutchings 2015, deposited in the Coleção de Invertebrados, Instituto Nacional de Pesquisas da Amazônia (INPA), Manaus, Amazonas State, Brazil.

***Material examined*:** 50 G♂, 2 ♂. FSP-USP, Brazil: São Paulo State, Presidente Epitácio Municipality, Peixe River (− 21.5633, − 51.9301), coll. Gomes et al. 10.xii.1997, det. Sá 2015: LAM. no. 01 (♂G); LAM. no. 02 (♂G). São Paulo State, Presidente Epitácio Municipality, João Baiano Farm (− 21.6464, − 52.0077), coll. Gomes et al. 2.v.1998, det. Sá 2015: LAM. no.05 (♂G); LAM. no. 06 (♂G); LAM. no. 07 (♂G). São Paulo State, Presidente Epitácio Municipality, Campinal (− 21.5735, − 51.9803), coll. Gomes et al. 4.i.1999, det. Sá 2015: LAM. no. 03 (♂G). Mato Grosso do Sul State, Bataguassu Municipality, Romualdo Farm (− 21.7411, − 52.2669), coll. Gomes et al. 2.viii.1997, det. Sá 2015: LAM. no. 08 (♂G). INPA, Brazil: Pará State, Almeirim Municipality, Arumanduba, Amazon River (− 1.48631, − 52.48706), coll. Hutchings et al. 19–20.x.2003, Hutchings and Sallum det.: ProV-047634 (♂G); ProV-047673 (♂G); ProV-047692 (♂G); ProV-047764 (♂G); ProV-047962 (♂G). Pará State, Prainha Municipality, Fazenda JK, Parana do Mouratuba, Amazon River (− 1.86209, − 53.72193), coll. Hutchings et al. 22–23.x.2003, det. Hutchings 2015: ProV-048997 (♂G, ♂); ProV-049054 (♂G); ProV-048945 (♂G). Pará State, Prainha Municipality, Boca do Rio Curuauna, Amazon River (− 2.39349, − 54.08755), coll. Hutchings et al. 24-25.x.2003, det. Hutchings 2015: ProV-049608 (♂G, ♂). Pará State, Almeirim Municipality, Paraíso, Paranaquara, Amazon River (− 1.74512, − 53.15400), coll. Hutchings et al. 2003, det. Hutchings 2015: ProV-055464 (♂G, ♂); ProV-055376 (♂G, ♂). Pará State, Obidos Municipality, Ilha do Amador “Ilha Grande”, Parana do Capivara, Amazon River (− 2.10015, − 55.3004), coll. Hutchings et al. 29–30.x.2003, det. Hutchings 2015: ProV-050860 (♂G); ProV-050878 (♂G); ProV-050909 (♂G); ProV-050910 (♂G); ProV-050943 (♂G); ProV-050964 (♂G); ProV-050974 (♂G); ProV-050978 (♂G); ProV-050982 (♂G); ProV-051027 (♂G); ProV-051033 (♂G); ProV-051049 (♂G); ProV-051053 (♂G); ProV-051055 (♂G); ProV-051079 (♂G); ProV-051092 (♂G); ProV-051104 (♂G); ProV-051106 (♂G); ProV-051181 (♂G). Pará State, Juruti Municipality, Recreio, Parana de Dona Rosa, Amazon River (− 2.07554, − 55.96586), coll. Hutchings et al. 30–31.x.2003, det. Hutchings 2015: ProV-053577 (♂G); ProV-053593 (♂G); ProV-053599 (♂G); ProV-053600 (♂G); ProV-053618 (♂G); ProV-053657 (♂G); ProV-053668 (♂G); ProV-053683 (♂G). Amazonas State, Itacoatiara Municipality, São Jorge, Parana da Eva, Amazon River (− 3.15751, − 59.32323), coll. Hutchings et al. 7–8.xi.2003, det. Hutchings 2015: ProV-057494 (♂G); ProV-057497 (♂G). USNM, Ecuador: (as *Cx*. *ensiformis*), coll. 3.xii.1981: EC8-1263(n 866) (♂G). Brazil: São Paulo State, Iguape Municipality (as *Cx*. *ensiformis*), coll. unknown, det. S.S. 1987: no. 050977-14 (♂G); São Paulo State, Cananéia Municipality (as *Cx*. *ensiformis*) coll. unknown, det. S.S. 1987: no. 050977-15 (♂G).

***ZooBank registration*:** The Life Science Identifier (LSID) for *Culex* (*Melanoconion*) *longistylus* n. sp. is urn:lsid:zoobank.org:act:C0FD06D0-B7E2-4775-A8BB-DC776858132F.

***Etymology*:** The specific epithet *longistylus* is a combination of the Latin adjective *longus* (long) and the Latin noun *stylus* (column, pillar), in reference to the long columnar process in the distal division of the subapical lobe of the male genitalia.

**Description**

**Fig. 14:**
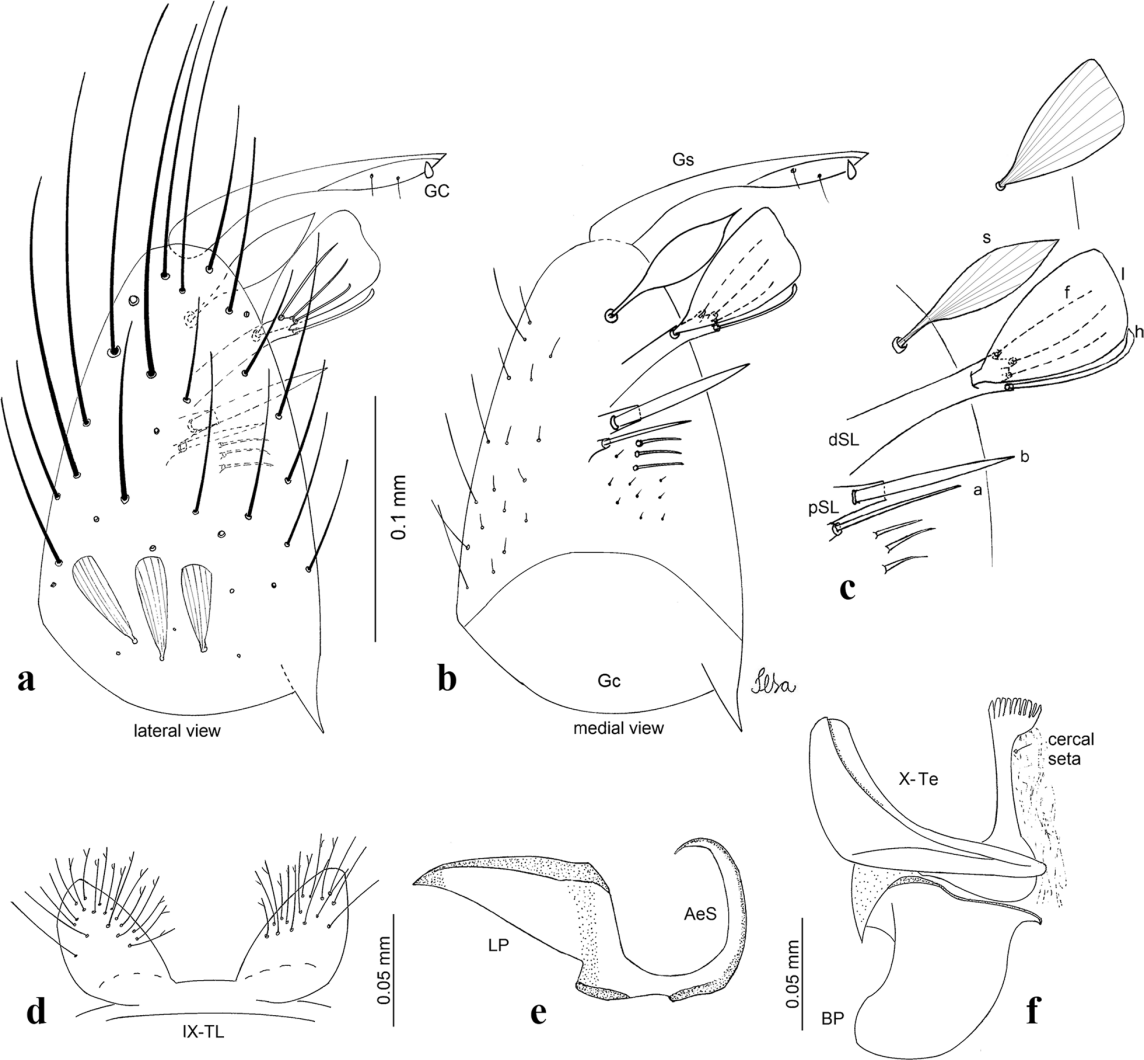
*Culex* (*Melanoconion*) *longistylus* n. sp., male genitalia. **a** Gonocoxite in lateral view. **b** Gonocoxite in medial view. **c** Setae on the subapical lobe of the gonocoxite. **d** Tergum IX lobe. **e** Aedeagus. **f** Proctiger. *Abbreviations*: Gc, gonocoxite; Gs, gonostylus; GC, gonostylar claw; dSL, distal division of subapical lobe; pSL, proximal division of subapical lobe; IX-TL, tergum IX lobe; AeS, aedeagal sclerite; LP, lateral plate; BP, basal plate; X-Te, tergum X

***Male*.** [Figs. 2h, 14] *Head*: antennal length 0.92–1.47 (1.23) (*n* = 6); proboscis entirely dark-scaled, length 1.23–1.64 (1.48) (*n* = 6); maxillary palpus dark-scaled, length 1.40–2.23 (1.73) (*n* = 6). Occiput with dark brown erect forked scales. *Thorax*: scutum covered with narrow, dark brown falcate scales, except prescutellar area with whitish scales. Median scutellar lobe with 6 large, dark setae; lateral lobes each with 4 setae. Pleural setae with 2 types of colouring: dark brown with bronzy reflections: 3–6 antepronotal; 3–5 prealar; and pleural setae golden, hyaline: 4 or 5 upper mesokatepisternal, 4 or 5 lower mesokatepisternal; 5 upper mesepimeral; lower mesepimeron with 1 long, strong seta. Pleura with patch of broad, white scales on upper mesokatepisternum; lower mesokatepisternum with few scales, extending dorsally on posterior margin. *Wing*: mostly dark-scaled, sometimes with minute patch of white scales at proximal end of vein C; length 2.08–2.45 (2.27) (*n* = 6). *Halter*: scabellum and pedicel whitish; capitellum pale brown with few golden scales. *Legs*: coxae pale; ventral surface of fore- and midfemur with longitudinal stripe of white scales; tibiae dark-scaled; joints of fermur-tibia and tibia-tarsomere I with ring of pale scales; tarsi entirely dark-scaled. *Abdomen*: tergum I with dark scales, terga III–VII dark-scaled with white basal bands. *Genitalia*: tergum IX as illustrated (Fig. 14d), tergal lobes each with 15–18 slender, simple, apically bifid setae arising from median portion; apex glabrous; distance between lobes as long as basal width of 1 lobe. Gonocoxite oblong, narrow, small; subapical lobe divided into 2 columnar divisions; proximal division with 2 parallel, apically pointed setae (*a* and *b*); seta *a* short, slender, inserted basal to seta *b*; seta *b* spatulate; gonocoxite with 3 short filiform setae with pointed apices on ventromesal surface; distal division with long columnar process, with 5 setae: 3 narrow, filiform, apically pointed setae, subequal in size (seta *f*), 1 long seta with hook-like apex (seta *h*), and 1 large, broad, ribbed asymmetrical seta arising subapically (seta *l*); 1 saber-like seta (seta *s*) arising apically. Gonostylus slender, slightly curved, tapering towards apex, apex moderately blunt, ventral surface with 2 apical hyaline setae; 1 short leaf-like gonostylar claw. Aedeagus with sclerotized, slightly pointed, dorsolaterally directed lateral process; ventral process straight; apical process convex. Proctiger with tergum X somewhat triangular in outline, inner process pointed. Paraproct elongate, crown with 9 or 10 simple blades. Cercal sclerite with 1 seta.

**Remarks**

Adults of *Culex longistylus* n. sp. differ from the adults of *Cx. atratus* in having dark-scaled wings, occasionally with an inconspicuous patch of white scales on the base of vein C, and dark-scaled terga III-VII with white basal bands. Based on male genitalia, *Cx*. *longistylus* n. sp. can be distinguished from the other species of the Atratus Group in possessing fine, subapically bifid setae interspersed with simple setae on tergum IX lobes, a long columnar process of the distal division with a large, broad and ribbed seta, lateral plate of the phallosome with a straight ventral process, and 3 short filiform setae on the ventromesal surface of the gonocoxite.

***Culex*(*Melanoconion*)*loturus*Dyar, 1925**

1925: *Culex* (*Melanoconion*) *loturus* Dyar, 1925: 241 [73] (♂) holotype ♂ deposited in the USNM. Type locality: Catatumbo River, Zulia, Venezuela.

*Culex* (*Melanoconion*) *loturus* of Dyar (1928: 342) [19] (♂, ♀, ♂G); Komp (1935: 9) [21] (synonymy with *Cx*. *zeteki*).

***Type material*:** Holotype, pinned adult male (USNM no. 28476), in poor condition, with associated genitalia on slide (USNM no. 30-1) deposited in the Diptera Collection, National Museum of Natural History (USNM), Washington, DC, USA.

***Distribution*:***Culex loturus* was collected in Venezuela at the margin of the Catatumbo River [19].

**Description**

**Fig. 15:**
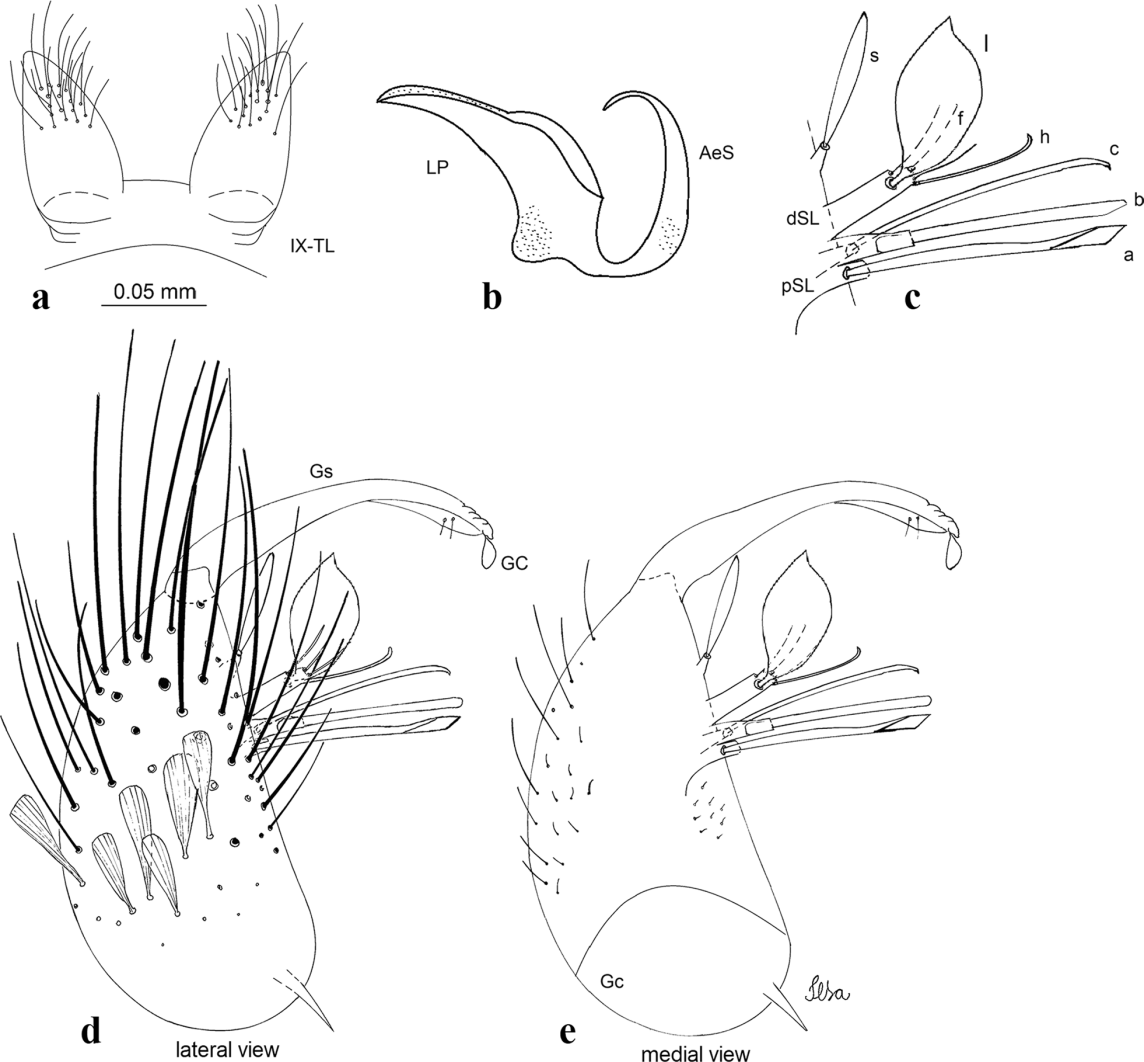
*Culex* (*Melanoconion*) *loturus*, male genitalia. **a** Tergum IX lobe. **b** Proctiger. **c** Setae on the subapical lobe of the gonocoxite. **d** Gonocoxite in lateral view. **e** Gonocoxite in medial view. *Abbreviations*: Gc, gonocoxite; Gs, gonostylus; GC, gonostylar claw; dSL, distal division of subapical lobe; pSL, proximal division of subapical lobe; IX-TL, tergum IX lobe; AeS, aedeagal sclerite; LP, lateral plate

***Male*.** [Fig. 15] Essentially similar to *Cx*. *zeteki*, except as follows: *Genitalia*: distal division of subapical lobe with median columnar process; seta *l* large, long, asymmetrical, ribbed; aedeagus with apical process of lateral plate pointed, without ripples.

***Female, pupa and larva*.** Unknown.

**Remarks**

*Culex loturus* was described by Dyar [73] based on males collected in Venezuela. Later, Dyar [19] mentioned the presence of one appendage on the proximal division of *Cx*. *loturus* and in *Cx*. *zeteki* he mentioned the presence of two appendages. Komp [21] synonymized *Cx*. *loturus* with *Cx*. *zeteki* Dyar [59] based on characteristics of the male genitalia, such as the presence of three setae on the proximal division in both species and other features in *Cx*. *loturus* which are identical to those of *Cx*. *zeteki*. Although *Cx*. *loturus* bears three setae on the proximal division, this species can be distinguished from *Cx*. *zeteki* in possessing a large subapical seta *l* on the distal division, and a slender apical process of the lateral plate that lacks ripples.

***Culex*(*Melanoconion*)*spinifer*Sá & Sallum n. sp.**

***Type locality*:** Pariquera-Açu Municipality (− 24.711237, − 47.873994), São Paulo State, Brazil. Adults were collected in the southeastern Atlantic Forest.

***Type material*:** Holotype, pinned adult male with dissected genitalia on slide (accession no. FSP-USP E-15901), with following collection data: Brazil, São Paulo State, Pariquera-Açu Municipality (− 24.711237, − 47.873994), coll. Forattini et al. 6.iii.1980, det. Sallum 1980, deposited in the Coleção Entomológica de Referência, Faculdade de Saúde Pública, Universidade de São Paulo (FSP-USP), São Paulo, São Paulo State, Brazil. Paratypes: 1 pinned adult male with dissected genitalia on slide (accession no. FSP-USP E-15902) from the same collection as the holotype; 1 pinned adult male with dissected genitalia on slide (accession no. FSP-USP E-15903), coll. Forattini et al. 19.iv.1979, det. Sallum 1980; 1 pinned adult male with dissected genitalia on slide (accession no. FSP-USP E-15904), coll. Forattini et al. 6.ix.1980, det. Sallum 1980, all deposited in FSP-USP.

***ZooBank registration*:** The Life Science Identifier (LSID) for *Culex* (*Melanoconion*) *spinifer* n. sp. is urn:lsid:zoobank.org:act:4C1F6F37-F488-4EBF-9F69-3523902A29F1.

***Etymology*:** From the Latin adjective *spinifer* meaning spiny. *Culex spinifer* is named in reference to the spicules present on the ventral process of the lateral plate of the aedeagus.

**Description**

**Fig. 16:**
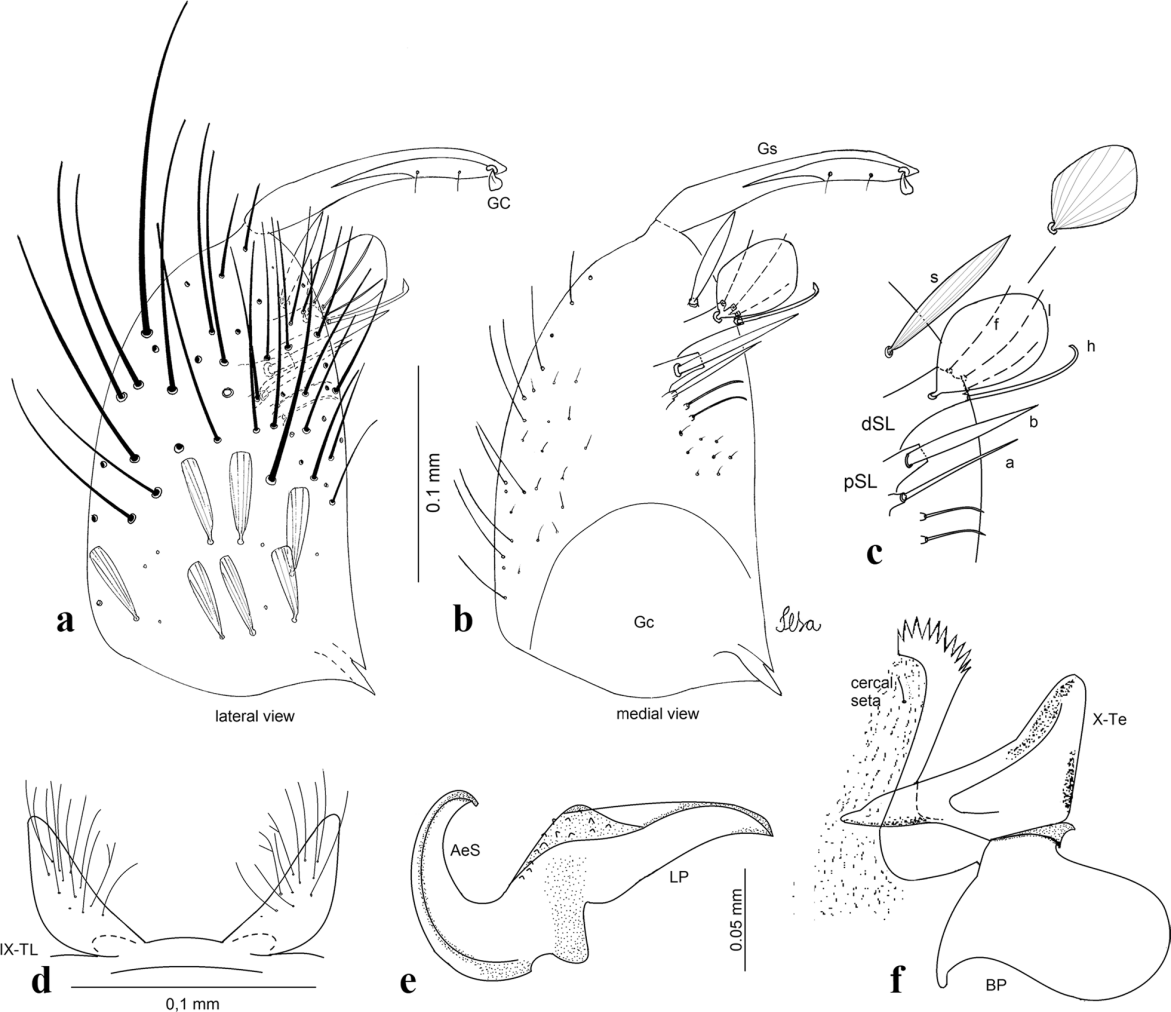
*Culex* (*Melanoconion*) *spinifer* n. sp., male genitalia. **a** Gonocoxite in lateral view. **b** Gonocoxite in medial view. **c** Setae on the subapical lobe of the gonocoxite. **d** Tergum IX lobe. **e** Aedeagus. **f** Proctiger. *Abbreviations*: Gc, gonocoxite; Gs, gonostylus; GC, gonostylar claw; dSL, distal division of subapical lobe; pSL, proximal division of subapical lobe; IX-TL, tergum IX lobe; AeS, aedeagal sclerite; LP, lateral plate; BP, basal plate; X-Te, tergum X

***Male*.** [Figs. 2i, 16] *Head*: antennal length 1.41–1.71 (1.59) (*n* = 4); proboscis dark-scaled, with inconspicuous median, dorsal patch of whitish scales; proboscis length 1.41–1.66 (1.52) (*n* = 4); maxillary palpus dark-scaled, length 2.28–2.05 (2.15) (*n* = 4); palpomere II with inconspicuous basal patch of whitish scales; palpomere III with small basal patch of whitish scales; palpomere IV with inconspicuous basal patch of whitish scales; palpomere V dark-scaled, with long, strong setae. Occiput with dark brown forked erect scales. *Thorax*: integument dark brown; scutum with narrow, dark brown forked scales, mainly on median prescutellar area and median scutal fossa; with whitish scales on anterior promontory and other prescutellar areas. Median scutellar lobe with 6 large, dark setae; lateral lobes each with 3 or 4 setae. Pleural setae with 2 types of colouring: dark brown: 3–6 antepronotal, 3 or 4 prealar; and pleural pale golden, slender setae: 4 or 5 upper mesokatepisternal, 4 or 5 lower mesokatepisternal, 4 or 5 upper mesepimeral; lower mesepimeron with 1 strong, long seta. Pleura with distinct patch of broad, white scales on upper mesokatepisternum; lower mesokatepisternum with few white scales. *Wing*: dark-scaled, with inconspicuous basal patch of whitish scales on vein C; large basal patch of whitish scales on vein R; wing length 2.93–2.65 (2.79) (*n* = 4). *Halter*: scabellum, pedicel and capitellum whitish. *Legs*: coxae pale; ventral surface of fore- and midfemur with a longitudinal stripe of white scales; tibiae dark-scaled; joints of femur-tibia and tibia-tarsomere I with ring of pale scales; tarsi entirely dark-scaled. *Abdomen*: tergum I with dark scales; terga III-VII dark-scaled, with basal bands of white scales. *Genitalia*: tergum IX lobes elongate, each with 7–10 slender simple setae and few apically bifid setae in median portion, apex glabrous. Distance between lobes equivalent to basal width of 1 lobe. Gonocoxite oblong, small; subapical lobe divided into 2 columnar divisions; proximal division with 2 pointed setae (*a* and *b*); seta *a* shorter, slender, inserted basal to seta *b*; seta *b* spatulate, robust and stronger than seta *a*; gonocoxite with 2 or 3 short, pointed, hyaline setae on ventromesal surface; distal division with short columnar process, with 5 setae: 3 narrow filiform, apically pointed setae of different in sizes (seta *f*), 1 longer hook-like seta (seta *h*), and 1 short, broad, asymmetrical seta arising subapically (seta *l*); 1 saber-like seta (seta *s*) arising apically. Gonostylus with broad leaf-like gonostylar claw with pointed apex, arising apically. Aedeagus with ventral process slightly convex and with spicules. Proctiger with tergum X with slightly pointed inner process.

**Remarks**

*Culex spinifer* n. sp. has spicules on the ventral process of the lateral plate similar to *Cx*. *dunni.* However, *Cx*. *spinifer* n. sp. differs from *Cx*. *dunni* in having elongate and slender ninth tergal lobes. Moreover, it has only two filiform setae on the proximal division of the subapical lobe and a somewhat triangular-shaped tergum X. *Culex spinifer* n. sp. differs from *Cx*. *comptus* n. sp., *Cx*. *longisetosus* n. sp. and *Cx*. *longistylus* n. sp. by having a short columnar process on the proximal division of the subapical lobe, a broad seta *l*, however shorter than filaments of seta *f*, and a large and broad gonostylar claw. Additionally, adults of *Cx*. *spinifer* n. sp. differ from those of *Cx*. *caribeanus* and *Cx*. *trigeminatus* in having the femora without pre-apical whitish rings and palpomere II with an inconspicuous proximal patch of whitish scales.

***Culex*(*Melanoconion*)*trigeminatus*Clastrier, 1970**

1970: *Culex* (*Melanoconion*) *trigeminatus*, Clastrier 1970: 473 [23] (♂) holotype ♂ deposited in the MNHN. Type locality: Forêt du Gallion, French Guiana.

*Culex* (*Melanoconion*) *trigeminatus* of Pecor et al. (1992: 27) [15] (distr.); Torres-Gutierrez & Sallum (2015: 18) [10] (distr.).

***Type material*:** Holotype, pinned adult male from Forêt du Gallion, French Guiana, collected on 19–20.iv.1968 (original number MNHN-3381-1), deposited in the Museum National dʼHistoire Naturelle (MNHN), Paris, France.

***Material examined*:** 56 specimens: 40 G♂, 24 Le, 37 Pe. FSP-USP, Brazil: São Paulo State, Pariquera-Açu Municipality, Braço Magro, coll. Sá et al. 2014, 20.viii.2014, det. Sá 2014: SP152-01 (Le, Pe, ♀); SP152-02 (Pe, ♂G, ♂); SP152-03 (Le, Pe, ♀); SP152-04 (Le, Pe, ♂G, ♂); SP152-05 (Le, Pe, ♂G, ♂); SP152-09 (Le, Pe, ♂G, ♂); SP152-12 (Le, Pe, ♂G, ♂); SP152-14 (Le, Pe, ♂); SP152-15 (Le, Pe, ♂G, ♂); SP152-19 (Le, Pe, ♂G, ♂); SP152-100 (Pe, ♀); SP152-101 (Pe, ♀); SP152-102 (Pe, ♀); SP152-104 (Pe, ♀); SP152-105 (Pe, ♂G, ♂); SP152-106 (Pe, ♀); SP152-107 (Pe, ♂G, ♂); SP152-108 (Pe, ♂G, ♂). São Paulo State, Pariquera-Açu Municipality, road to Braço Magro farm, Lagoon in forest environment, coll. Sá et al. 2014, 16.ix.2014, det. Sá 2014: SP157-03 (Le, Pe, ♀); SP157-08 (Le, Pe, ♂G, ♂); SP157-10 (Le, Pe, ♂G, ♂); SP157-12 (Le, Pe, ♀); SP157-16 (Le, Pe, ♂G, ♂); SP157-18 (Le, Pe, ♂G, ♂); SP157-19 (Le, Pe, ♂G, ♂); SP157-101 (♂G, ♂); SP157-104 (Pe, ♂G, ♂); SP157-107 (Pe, ♀). São Paulo State, Pariquera-Açu Municipality, road to Braço Magro farm, stream on forest A, coll. Sá et al. 16.ix.2014, det. Sá 2014: SP158A-01 (Le, Pe, ♂G, ♂); SP158A-08 (Le, Pe, ♂G, ♂); SP158A-09 (Le, Pe, ♂G, ♂); SP158A-16 (Le, Pe, ♂G, ♂). São Paulo State, Pariquera-Açu Municipality, Braço Magro, Lagoon, coll. Sá et al. 2016, det. Sá 2016: SP184-22 (♂G). São Paulo State, Cananéia Municipality, Taquari (− 25.015000, − 47.926944), coll. Forattini et al. 25.iii.1980, det. Sallum 1980: no. 241 (♂G, ♂); no. 225 (♂G, ♂). São Paulo State, Cananéia Municipality, Itapuã Farm (− 24.888783, − 47.851686), coll. Forattini et al. 6.iv.1981, det. Sallum 1981: no. 136 (♂G, ♂). São Paulo State, Pariquera-Açu Municipality (− 24.715000, − 47.880833), coll. Forattini et al. 8.i.1981, det. Sallum 1981: no. 2242 (♂G, ♂); no. 2491 (♂G, ♂). São Paulo State, Iguape Municipality (− 24.708056, − 47.555278), coll. Forattini et al. 6.x.1982, det. Sallum 1982: no. 3251 (♂G, ♂). São Paulo State, Cananéia Municipality, Folha Larga Farm − 24.89273, − 47.919048), coll. Forattini et al. 19.iv.1983, det. Sallum 1983: no. 3470 (♂G, ♂). São Paulo State, Cananéia Municipality, Vilarinho farm (− 24.951551, − 47.977989), coll. Forattini et al. 7.ii.1984, det. Sallum 1984: no. 3515 (♂G, ♂). São Paulo State, Cananéia Municipality, Itapitangui (− 24.935105, − 47.961728), coll. Forattini et al. 11.iv.1985, det. Sallum 1985: EP035-01 (Le, Pe, ♂G, ♂), EP035-08 (Le, Pe, ♀).

***Distribution*:***Culex trigeminatus* has been collected in Brazil and French Guiana [67]. In Brazil, the species was found in the municipalities of São Paulo [74], Cananéia, Iguape and Pariquera-Açu, São Paulo State and in Belém Municipality [75], Pará State.

**Description**

***Female*.***Head*: antennal length 1.28–1.70 (1.46) (*n* = 5); proboscis dark-scaled, with median, dorsal patch of whitish scales, length 1.23–1.33 (1.28) (*n* = 5); maxillary palpus dark-scaled, length 0.21–0.25 (0.23) (*n* = 5). Occiput with erect, forked, pale brown scales. *Thorax*: scutum with narrow, dark brown to black falcate scales and narrow, whitish falcate scales on scutal fossa, dorsocentral, anterior promontory and supraalar areas forming a pattern. Scutellar scales whitish, median lobe with 5 or 6 setae, lateral lobes each with 3 setae. Pleural setae with 2 types of colouring: dark brown: 4–6 antepronotal, 4 or 5 prealar; and pleural setae golden: 4 upper mesokatepisternal, 3 or 4 lower mesokatepisternal, 4 upper mesepimeral, 1 large lower mesepimeral. Pleura with distinct patch of broad, whitish scales. *Wing*: dark-scaled, vein C with small proximal patch of whitish scales, vein R with large proximal patch of whitish scales; wing length 2.63–2.84 (2.67) (*n* = 5). *Halter*: scabellum, pedicel and capitellum pale brown. *Legs*: fore- and midfemur with conspicuous preapical ring of white scales. *Abdomen*: terga II-VII with basal bands of white scales, tergum VIII dark-scaled.

**Fig. 17:**
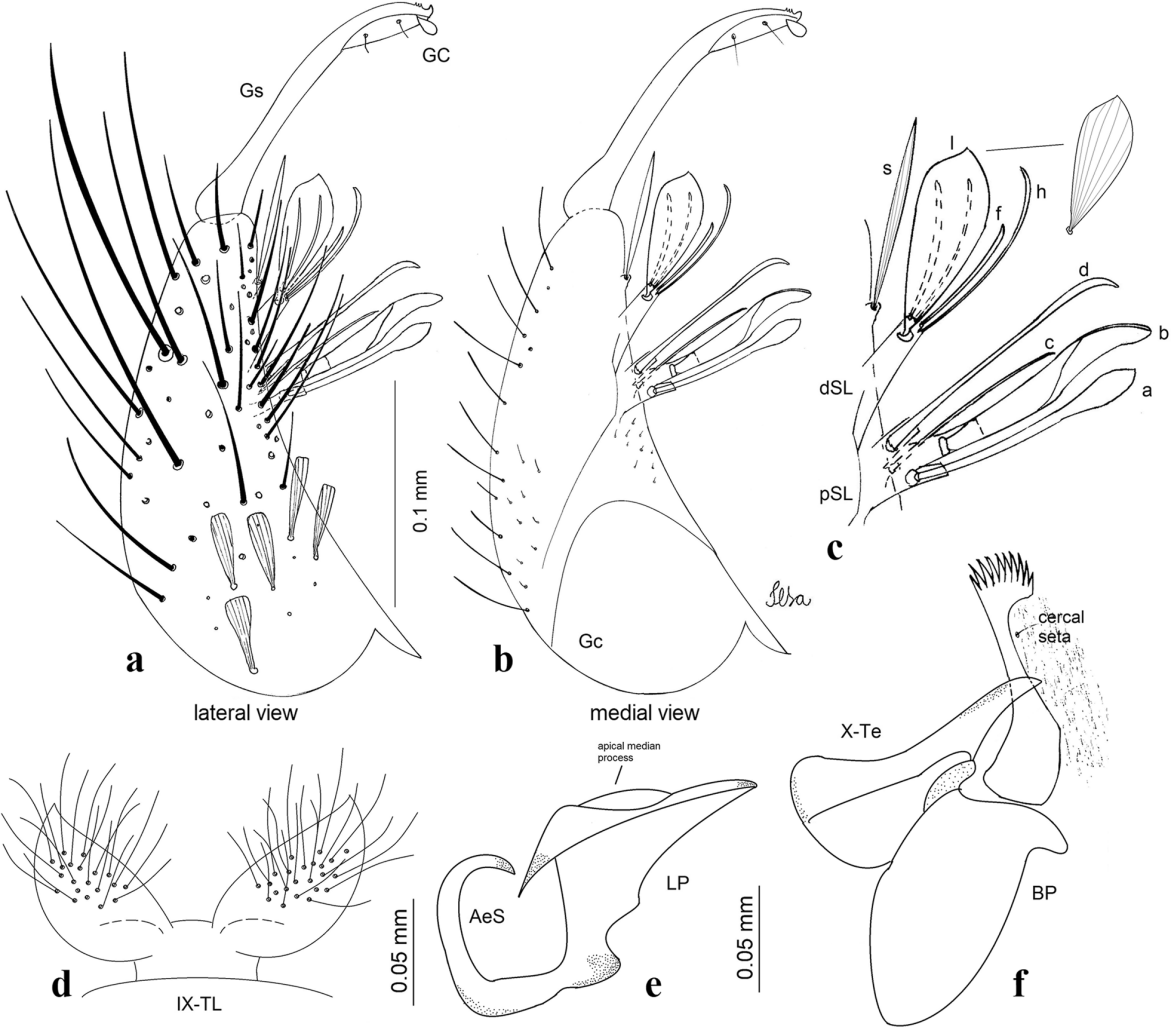
*Culex* (*Melanoconion*) *trigeminatus*, male genitalia. **a** Gonocoxite in lateral view. **b** Gonocoxite in medial view. **c** Setae on the subapical lobe of the gonocoxite. **d** Tergum IX lobe. **e** Aedeagus. **f** Proctiger. *Abbreviations*: Gc, gonocoxite; Gs, gonostylus; GC, gonostylar claw; dSL, distal division of subapical lobe; pSL, proximal division of subapical lobe; IX-TL, tergum IX lobe; AeS, aedeagal sclerite; LP, lateral plate; BP, basal plate; X-Te, tergum X

***Male*.** [Figs. 2j, 17] Essentially similar to female, except for following characters: *Head*: antenna verticillate, length 0.96–1.12 (1.11) (*n* = 5); proboscis dark-scaled, with median patch of whitish scales, proboscis length 1.47–1.63 (1.53) (*n* = 5); maxillary palpus length 1.71–2.28 (1.96) (*n* = 5), palpomere III with basal patch of whitish scales; palpomeres IV and V with small basal patch of whitish scales. *Wing*: length 2.32–2.81 (2.51) (*n* = 5). *Genitalia*: tergum IX lobes with convex outer edge, apex glabrous, median portion each with 20–22 slender, simple setae; distance between lobes shorter than half basal width of 1 lobe. Gonocoxite narrow, oblong; proximal division with 4 parallel setae (*a*, *b*, *c* and *d*): seta *a* more basal, spoon-shaped; seta *b* robust, spatulate, inserted on tubercle; seta *c* thin, slender, filiform, inserted between setae *b* and *d*; seta *d* borne on tubercle apical to seta *b*, filiform, long, with slightly narrowed apex. Distal division with medium-sized, elongate columnar process, with 5 setae: 3 filiform, narrow, pointed, apically inserted, subequal sized (seta *f*), 1 filiform, hook-like apex (seta *h*), and 1 large, broad, asymmetrical ribbed seta with apex slightly pointed on median portion, arising subapically (seta *l*); and 1 saber-like, ribbed seta (seta *s*) arising apically. Gonocoxite with slender, hyaline, short, inconspicuous setae on ventromesal surface. Gonostylus as in *Cx*. *atratus*, except for dorsal surface of the apex which may bear 2 or 3 superficial, inconspicuous emarginations. Lateral plate of aedeagus with rounded apical process, ventral process with short pointed projection directed ventrobasally. Proctiger with tergum X somewhat triangular in outline, inner process pointed and short.

***Pupa*.** [Figs. 3e, 4e] *Cephalothorax*: seta 4-CT 3-branched; seta 5-CT 4-branched; seta 8-CT 6-branched; seta 12-CT 3- or 4- branched. Trumpet long, slender; pinna small, opening circular, pinna length 0.05–0.08 (0.06) (*n* = 9), distal margin opposite meatal cleft with small notch; tracheoid area extending 0.15–0.25 (0.22) (*n* = 9) from base; trumpet index 14.8–30.5 (20.9) (*n* = 9). *Abdomen*: seta 9-VIII with 2 simple branches; paddle index 1.40–1.71 (1.50) (*n* = 9).

***Larva*.** [Figs. 5e, 6e] *Head*: length 0.65–0.73 (0.69) (*n* = 9), width 1.05–1.12 (1.09) (*n* = 9). Antennal length 0.52–0.63 (0.56) (*n* = 9); seta 1-A inserted 0.39–0.46 (0.41) (*n* = 9) from antennal base. Seta 5-C with 5 short branches not reaching 6-C insertion; seta 10-C 4-branched; seta 13-C 3-branched. *Abdomen*: comb of segment VIII with 18–22 scales of different sizes arranged in 2 or 3 irregular rows: upper rows with small, pointed scales; lower row with 7–9 large, pointed scales. Segment X length 0.30–0.36 (0.33) (*n* = 9), siphon/saddle index 4.62–5.28 (4.88) (*n* = 9). *Siphon*: long, slender, index 7.7–13.0 (10.8) (*n* = 9); pecten with 13 spines on basal 0.30 of siphon. Seta 1-S usually with 4 ventral pairs and 2 dorsal pairs.

***Bionomics*.** Immatures of *Cx*. *trigeminatus* were collected in large, shaded lagoons with aquatic vegetation and, in small, flooded, shaded depressions and floodplain terraces of streams in Atlantic Forest, associated with *Cx*. *albinensis* and *Cx*. *zeteki*.

**Remarks**

*Culex trigeminatus* was described by Clastrier [23] based on an adult male from French Guiana. *Culex trigeminatus* is more closely related to *Cx*. *caribeanus* within the Atratus Group, especially regarding adult specimens. However, *Cx*. *trigeminatus* differs from *Cx*. *caribeanus* in having palpomeres I and II dark-scaled, palpomere III with small basal whitish patch, palpomeres IV and V with inconspicuous whitish basal patches, and wings with proximal patches of white scales on veins C and R. The male genitalia of *Cx*. *trigeminatus* differ from those of *Cx*. *caribeanus* by having the median portion of the apex of seta *l* (distal division of subapical lobe) slightly pointed, robust and with blunt apex and proximal division with seta *b* strong, the lateral plate of the aedeagus having a rounded apical process and a ventral process with short, pointed projection directed ventrobasally. Fourth-instar larvae of *Cx*. *trigeminatus* differ from those of the other species of the Atratus Group by having seta 5-C with short branches that do not reach the insertion of seta 6-C and a siphon with only two pairs of dorsal setae. Furthermore, *Cx*. *trigeminatus* differs from *Cx*. *ensiformis* in possessing strongly serrated comb scales and short pecten spines. With respect to pupae, *Cx*. *trigeminatus* can be distinguished from the other species by having a slender trumpet and with the pinna small and appearing heart-shaped in dorsal view.

***Culex*(*Melanoconion*)*zeteki*Dyar, 1918**

1918: *Culex* (*Melanoconion*) *zeteki* Dyar, 1918: 122 [59] holotype ♂ (as *Cx*. *zeteci*) deposited in the USNM. Type locality: Gatún, Canal Zone, Panama.

*Culex* (*Melanoconion*) *zeteki* of Dyar (1928: 339) [19] (♂ as *Cx*. *zeteci*); Komp (1935: 9) [21] (♂ as *Cx*. *zeteci*); Rozeboom & Komp (1950: 98) [6] (tax., emend. to *Cx*. *zeteki*); Pecor et al. (1992: 56) [15] (distr.); Torres-Gutierrez & Sallum (2015: 18) [10] (distr., type info.).

***Type material*:** Holotype, pinned adult male (USNM no. 21778) with dissected genitalia (USNM no. 953) on slide, in poor condition, deposited in the Diptera Collection, National Museum of Natural History (USNM), Washington, DC, USA.

***Material examined*:** 37 specimens: 15 Le, 20 Pe, 10 ♀, 10 ♂. (FSP-USP): Brazil: São Paulo State, Pariquera-Açu Municipality, Pariquera-Mirim (− 24.729867, − 47.813300), coll. Sá et al. 2014, 21.viii.2014, det. Sá 2014: SP155-29 (Le, Pe, ♂G, ♂). São Paulo, Pariquera-Açu, coll. Sá et al. 2014, 16.ix.2014, det. Sá 2014: SP157-105 (Pe, ♀); SP158A-04 (Le, Pe, ♂G, ♂); SP158A-05 (Le, Pe, ♂G, ♂); SP158A-06 (Le, Pe, ♂G, ♂); SP158A-07 (Le, Pe, ♂G, ♂). Minas Gerais State, Cláudio Municipality, Marcelo Farm, Várzea da Rocinha (− 20.44384, − 44.76532), coll. Bergo et al. 2010, 13.iv.2010, det. Sallum 2014: MG50-10 (Le, Pe, ♂G, ♂). São Paulo State, Dourado Municipality (− 22.100000, − 48.317778), coll. Forattini et al. 7.i.1981, det. Sallum 1981: no.147 (♂G); no.150 (♂G, ♂); no.151 (♂G); no.152 (♂G, ♂); no.156 (♂G, ♂); no.157 (♂G). São Paulo, Cananéia Municipality, Iririaia-Açu (− 24.871599, − 47.907568), coll. Forattini et al. 18.i.1984, det. Sallum 1984: HEP352-07 (Le, Pe, ♀). São Paulo, Cananéia, Itapitangui (− 24.935105, − 47.961728), coll. Forattini et al. 11.iv.1984, det. Sallum 1984: HEP387-01 (Le, Pe, ♂G, ♂); HEP387-03 (Pe, ♂G); HEP387-04 (Le, Pe, ♂G, ♂); HEP394-05 (Le, Pe, ♀). São Paulo, Cananéia, Folha larga (− 24.89273, − 47.919048), coll. Forattini et al. 26.vii.1985, det. Sallum 1985: EP0005-01 (Pe, ♂G); EP0005-02 (Le, Pe, ♀); EP0005-04 (Pe); EP0005-05 (Le, Pe, ♂G); EP0005-06 (Le, Pe, ♀); EP0005-07 (Le, Pe, ♀); EP0005-09 (Le, Pe, ♀). São Paulo, Cananéia, Itapitangui (− 24.935105, − 47.961728), coll. Forattini et al. 25.x.1988, det. Sallum 1988: EP0003-06 (Pe, ♂G). Amazonas State, Humaitá Municipality, Realidade (− 7.106919, − 63.115172), coll. Chaves et al. 22.vii.2016, det. Sá 2017: Coleta07-Humaitá-03 (♀); Coleta07-Humaitá-04 (♀); Coleta07-Humaitá-05 (♀); Coleta07-Humaitá-06 (♀); Coleta07-Humaitá-74 (♀). Amazonas State, Lábrea Municipality, Umari (− 7.524958, − 64.697702) coll. Sallum et al. viii.2015, det. Sá 2017: AM47-06 (♂). (INPA): Amazonas State, Manaus Municipality, Acampamento Colosso, Fazenda Esteio (− 2.40417, − 59.86361), coll. Hutchings & Aquino 2002, det. Sallum & Hutchings: Fam-000630 (♂G); Fam-000939 (♂G); Fam-002679 (♂G). Amazonas State, Jutai Municipality, São Raimundo, Parana do Cervalho, Solimões River (− 2.70907, − 66.89931), coll. Hutchings et al. 16–17.ix.2003, det. Sá 2017: ProV-007250 (♂G); ProV-007254 (♂G).

***Distribution*:***Culex zeteki* has been found in Belize [15], Brazil [76], Colombia [65], French Guiana [67], Honduras [77], Nicaragua [15], Paraguay [15], Panama [15], Suriname [7, 15, 69] and Venezuela [15]. In Brazil, the species was collected in Amazonas State [50, 70, 76], Mato Grosso State [78], Minas Gerais State, Paraná State [79] and São Paulo State [80, 81].

**Description**

***Female*.***Head*: antennal length 0.81–1.31 (1.08) (*n* = 5); proboscis dark-scaled, length 1.07–1.33 (1.20) (*n* = 5); maxillary palpus dark-scaled, length 0.16–0.26 (0.22) (*n* = 5). Occiput with brown, erect, forked scales. *Thorax*: scutum with narrow, bronzy falcate scales. Median lobe of scutellum with 6 setae, lateral lobes each with 4 setae. Pleural setae with 2 types of colouring: dark brown: 4–6 antepronotal, 4 or 5 prealar; and pleural setae golden: 3 or 4 upper mesokatepisternal, 3 or 4 lower mesokatepisternal, 4 upper mesepimeral, 1 large lower mesepimeral. Pleura with indistinct broad patch of whitish scales. Mesepimeral integument dark, with distinct median whitish area completely separating darker upper and lower areas. *Wing*: dark-scaled, length 2.23–2.88 (2.55) (*n* = 5). *Halter*: scabellum, pedicel and capitellum whitish. *Legs*: as in *Cx*. *atratus*. *Abdomen*: tergum II-VII with basal bands of white scales, tergum VIII dark-scaled.

**Fig. 18:**
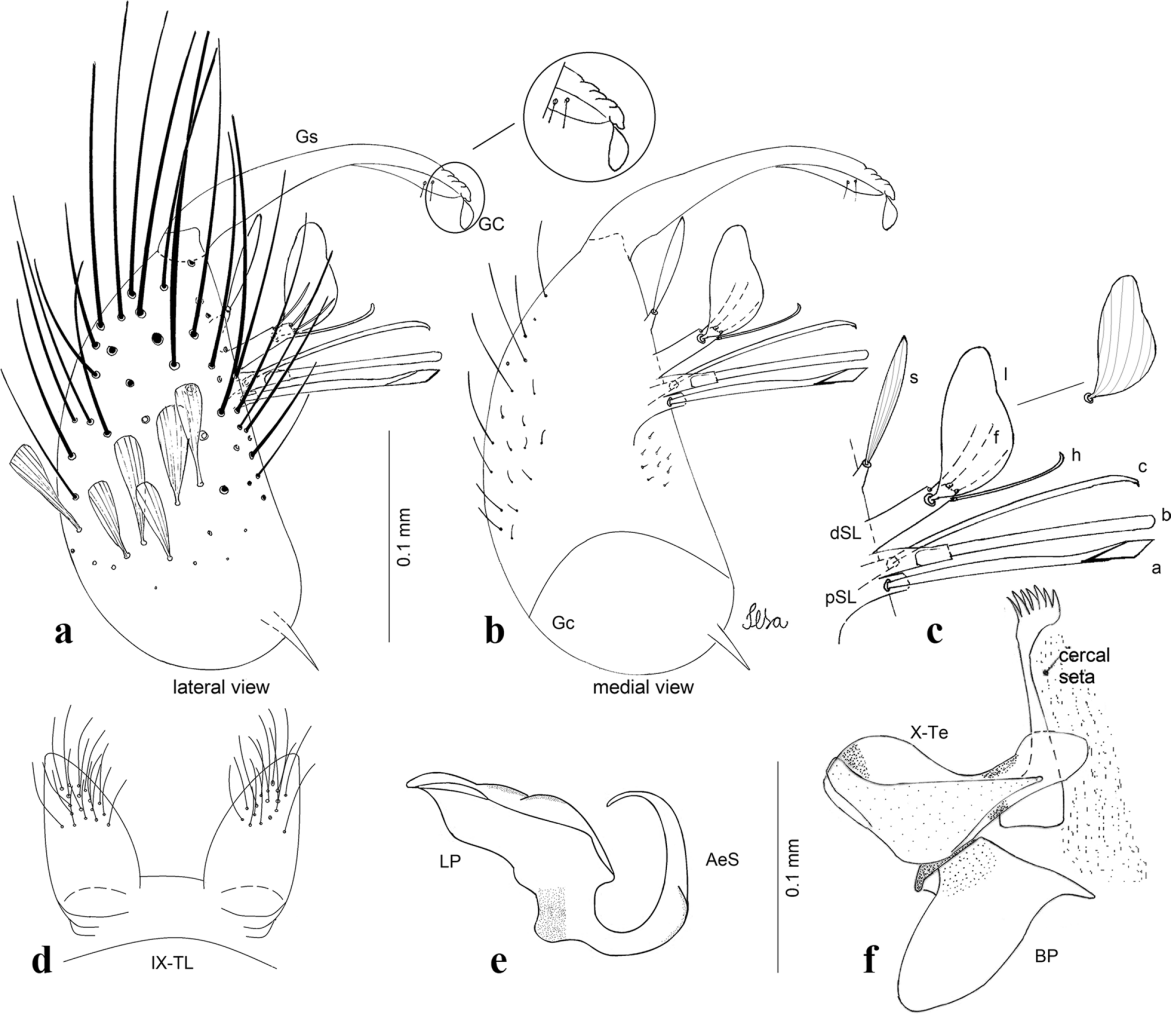
*Culex* (*Melanoconion*) *zeteki*, male genitalia. **a** Gonocoxite in lateral view. **b** Gonocoxite in medial view. **c** Setae on the subapical lobe of the gonocoxite. **d** Tergum IX lobe. **e** Aedeagus. **f** Proctiger. *Abbreviations*: Gc, gonocoxite; Gs, gonostylus; GC, gonostylar claw; dSL, distal division of subapical lobe; pSL, proximal division of subapical lobe; IX-TL, tergum IX lobe; AeS, aedeagal sclerite; LP, lateral plate; BP, basal plate; X-Te, tergum X

***Male*.** [Figs. 2k, 18] Essentially similar to female, except for following characters: *Head*: antennal length 0.96–1.32 (1.15) (*n* = 5); proboscis entirely dark-scaled, length 1.24–1.81 (1.63) (*n* = 5); maxillary palpus dark-scaled, length 1.68–2.19 (1.85) (n = 5). *Wing*: length 2.28–2.74 (2.64) (*n* = 5). *Genitalia*: tergum IX lobes elongate, each with 20–22 slender, simple setae on median portion, apex glabrous, slightly pointed. Distance between lobes shorter than basal width of 1 lobe. Gonocoxite oblong, narrow; proximal division with 3 long, parallel setae (*a*, *b* and *c*): seta *a* long, slender with “opened” apex; seta *b*, long with rounded apex; seta *c* slender, filiform, with curved apex. Distal division with median columnar process with 5 setae: 3 filiform, narrow, apically pointed and differently sized setae (setae *f*), 1 long seta hook-like at apex (seta *h*), and 1 large, long, asymmetrical seta arising subapically (seta *l*); 1 saber-like seta (seta *s*) arising apically; gonocoxite with short, inconspicuous, hyaline setae on ventromesal surface. Gonostylus as in *Cx*. *atratus*, except for dorsal surface of apex with 3 or 4 conspicuous folds and large gonostylar claw. Aedeagus with apical process with rounded ripples; ventral process slightly straight. Proctiger with tergum X asymmetrical, with rounded outer and inner processes.

***Pupa*.** [Figs. 3f, 4f] *Cephalothorax*: seta 5-CT 4-branched; seta 7-CT double. Trumpet slender; pinna small, asymmetrical, length 0.09–0.15 (0.12) (*n* = 10), distal margin opposite the meatal cleft with small, inconspicuous emargination; tracheoid area extending 0.15–0.21 (0.18) (*n* = 10) from base; trumpet index 12.0–20.0 (15.9) (*n* = 10). *Abdomen*: seta 9-VIII with 3 simple branches; paddle index 1.57–2.03 (1.69) (*n* = 10).

***Larva*.** [Figs. 5f, 6f] *Head*: length 0.66–0.77 (0.71) (*n* = 10), width 1.04–1.13 (1.09) (*n* = 10). Antennal length 0.49–0.57 (0.53) (*n* = 10); seta 1-A inserted 0.35–0.39 (0.37) (*n* = 10) from antennal base. Seta 5-C with 4 long branches; seta 13-C double; seta 14-C with 2 strong branches. *Abdomen*: comb of segment VIII with 28–34 scales equal in size arranged in 3 or 4 irregular rows. Segment X length 0.30–0.37 (0.33) (*n* = 10), siphon/saddle index 3.90–4.88 (4.43) (*n* = 10). *Siphon*: long, slender, index 6.5–8.9 (8.1) (*n* = 10); pecten with 12 spines on basal 0.30 of siphon. Seta 1-S usually with 4 ventral pairs and 4 dorsal pairs.

***Bionomics*.** Immature specimens of *Cx*. *zeteki* were collected in shaded, stagnant lagoons with abundant aquatic vegetation.

**Remarks**

*Culex zeteki* was described by Dyar [59] as *Cx*. *zeteci*. Rozeboom & Komp [6] corrected the description of the male genitalia and emended the spelling of the name to *Cx*. *zeteki*. *Culex zeteki* differs from *Cx*. *columnaris* n. sp. in having the gonostylus with folds on the dorsal surface. Fourth-instar larvae of *Cx*. *zeteki* can be distinguished by having comb scales with lateral fringes on the middle of the lateral margins and seta 5-C with 4 or 5 long branches which may reach seta 7-C insertion. Pupae of *Cx*. *zeteki* can be distinguished from the other species of the group by possessing a slender trumpet with a small pinna and having the distal margin opposite the meatal cleft with an inconspicuous rounded emargination.

The primary diagnostic characters of the larval and pupal forms are summarized in Tables 1 and 2, respectively. The diagnostic characters of the male genitalia and adults are summarized in Tables 3 and 4, respectively.

**Table 1:** Comparative data for the main morphological features of the known pupae of species of the Atratus Group

Feature	*Cx*. *atratus*	*Cx*. *comptus* n. sp.	*Cx*. *dunni*	*Cx*. *ensiformis*	*Cx*. *trigeminatus*	*Cx*. *zeteki*
Seta 9-VIII	4 aciculate branches	3 simple branches	2 simple branches	3 simple branches	2 simple branches	3 simple branches
Trumpet index	7.1	13.8–16.2 (15.4)	12.4–20.0 (14.5)	16.2–29.7 (24.2)	14.8–30.5 (20.9)	12.0–20.0 (15.9)
Pinna	Small, V-shaped	Small, irregular-shaped	Median, with large emargination on distal margin	Small, circular, with small emargination on distal margin	Small, irregular-shaped	Small, irregular-shaped, with small circular emargination on distal margin

**Table 2:** Comparative data for the main morphological features of the known fourth-instar larvae of species of the Atratus Group

Feature	*Cx*. *atratus*	*Cx*. *comptus* n. sp.	*Cx*. *dunni*	*Cx*. *ensiformis*	*Cx*. *trigeminatus*	*Cx*. *zeteki*
Seta 5-C	Reaching 6-C insertion	Exceeding 6-C insertion	Exceeding 6-C insertion	Exceeding 6-C insertion	Short, not reaching 6-C insertion	Exceeding 6-C insertion
Comb scales	Subequal in size	Subequal in size	Subequal in size	Different sizes	Different sizes	Subequal in size
Pecten spines	With large, coarse marginal denticles	Narrow, with smaller, finer marginal spicules	With large, coarse marginal denticles	Long and slender, with fine marginal denticles (serration)	Elongate, with coarse marginal denticles	Short, broad basally and gradually narrowed to apex, with coarse marginal denticles
Seta 1-S arising dorsolaterally	4 pairs	4 pairs	6 pairs	4 pairs	2 pairs	4 pairs

**Table 3:** Comparative data for the main morphological features of the male genitalia of species of the Atratus Group

Species	Columnar process pSL	Columnar process dSL	Seta *s* of gonocoxite	Tergum X	Gonostylar claw	Gonocoxite^a^	Ventral process LP	Seta *l* dSL
*Cx. atratus*	Simple	Short	With slender apex, without peduncle	Subtriangular, with pointed inner process	Long, narrow	With 4 or 5 flattened, long, broad, curved setae	Simple	Long
*Cx. caribeanus*	Simple	Long	With slender apex and short peduncle	Subtriangular, with pointed inner process	Short, narrow	With 4 or 5 long, filiform setae	With broad, long, pointed projection	Long
*Cx. columnaris* n. sp.	Long, divided subapically	Medium-sized	With wide apex, without peduncle	Irregular with rounded prominence	Short, narrow	With 2 short, hyaline setae	Simple	Long
*Cx. comptus* n. sp.	Simple	Long	With wide apex, without peduncle	Subtriangular, with less pointed inner process	Very short	With 3 or 4 median, filiform setae	Simple	Long
*Cx. dunni*	Simple	Short	With slender apex, without peduncle	Elongate, sinuous, with pointed, narrow inner process	Long, narrow	With few short, filiform setae	With spicules	Long
*Cx. ensiformis*	Simple	Short	With slender apex and short peduncle	Subtriangular, with less pointed inner process	Long, narrow	With 3 or 4 short, filiform setae	With small convexity	Short
*Cx. exedrus*	Simple	Short	With slender apex, without peduncle	Elongate, sinuous, with pointed inner process	Long, narrow	With few short, filiform setae	–	Long
*Cx. longistylus* n. sp.	Simple	Long	With slender apex, without peduncle	Subtriangular, with pointed inner process	Short, narrow	With 3 or 4 hyaline, filiform setae	Simple	Long
*Cx. loturus*	Simple	Medium-sized	With slender apex and long peduncle	–	Long, broad	With several short, filiform setae	Simple	Long
*Cx. longisetosus* n. sp.	Simple	Long	With slender apex, without peduncle	Subtriangular, with less pointed inner process	Short, tiny	With 4 large, spatulate setae	Simple	Long
*Cx. spinifer* n. sp.	Simple	Short	With slender apex, without peduncle	Subtriangular, with less pointed inner process	Long, broad	With 2 hyaline, filiform setae	With spicules	Short
*Cx. trigeminatus*	Simple	Medium-sized	With slender apex and short peduncle	Subtriangular, with pointed inner process	Long, less broad	With several short, filiform setae	With short, pointed projection	Long
*Cx. zeteki*	Simple	Medium-sized	With slender apex and long peduncle	Irregular, with rounded proeminence	Long, broad	With several short, filiform setae	Simple	Long

^a^Ventromesal surface

*Abbreviations*: pSL, proximal division of the subapical lobe; dSL, distal division of the subapical lobe; LP, lateral plate of the aedeagus; –, none examined

**Table 4:** Comparative data for the main morphological features of the adult of species of the Atratus Group

Species/feature	Palpomere I (male)	Palpomere III (male)	Proboscis	Wings dorsal scaling	Mesepimeron (tegument)	Scutum
*Cx*. *atratus*	Dark-scaled	Dark-scaled	Dark-scaled	Dark-scaled	Dark	With bronzed scales
*Cx*. *caribeanus*	White-scaled	With median large, whitish patch	With median large patch of whitish scales	Dark-scaled	Dark	With bronzed scales; whitish scales on anterior promontory, dorsocentral, supraalar, scutal fossa prescutellar areas forming a pattern
*Cx*. *columnaris* n. sp.	Dark-scaled	Dark-scaled	Dark-scaled	Dark-scaled	Dark	With brown scales with golden reflection; prescutellar area with whitish scales
*Cx*. *comptus* n. sp.	Dark-scaled	Dark-scaled	Dark-scaled	C with small proximal patch of whitish scales; Sc with indistinct basal patch of white scales; R with 2 proximal patches of whitish scales separated by median patch of dark scales	Dark	With dark brown/black scales; pale golden scales on anterior promontory, scutal fossa, dorsocentral, prescutellar, supraalar areas forming a pattern
*Cx*. *dunni*	Dark-scaled	Dark-scaled (occasionally with indistinct whitish basal patch)	Dark-scaled	C with inconspicuous proximal patch of whitish scales; R with two proximal patches of white scales separated by large patch of dark scales	Dark	With bronzed scales; occasionally, with whitish scales on dorsocentral, supraalar, scutal fossa prescutellar areas
*Cx*. *ensiformis*	Dark-scaled	Dark-scaled	Dark-scaled	C with proximal patch of white scales; R with two proximal patches of white scales separated by small patch of dark scales	Dark	With bronzed, golden scales on acrostichal, dorsocentral areas; whitish scales on anterior promontory, prescutellar fossa areas forming a pattern
*Cx*. *longistylus* n. sp.	Dark-scaled	Dark-scaled	Dark-scaled	C with proximal patch of white scales	Dark	With dark brown scales; prescutellar area with whitish scales
*Cx*. *longisetosus* n. sp.	Dark-scaled	With indistinct, whitish basal patch	Dark-scaled	Dark-scaled	Dark	With dark brown scales; anterior promontory and prescutellar areas with whitish scales
*Cx*. *spinifer* n. sp.	Dark-scaled	With small, whitish basal patch	With median, dorsal, indistinct, whitish patch	C with inconspicuous proximal patch of whitish scales; R with large proximal patch of whitish scales	Dark	With dark brown scales on median prescutellar, median scutal fossa areas; whitish scales on anterior promontory and other prescutellar areas
*Cx*. *trigeminatus*	Dark-scaled	With whitish basal patch	With median large patch of whitish scales	C with small proximal patch of whitish scales; R with large proximal patch of whitish scales	Dark	With dark brown/black scales, whitish scales on scutal fossa, dorsocentral, anterior promontory and supraalar areas forming a pattern
*Cx*. *zeteki*	Dark-scaled	Dark-scaled	Dark-scaled	Dark-scaled	Dark, with median whitish stain	With bronzed scales

**Keys for identification of the Atratus Group and the species of the group**

(i) **Keys based on adult morphology**
1 Vertex with narrow, curved or linear decumbent scales; broad decumbent scales restricted to small lateral patches…2
– Vertex with broad decumbent scales…Melanoconion Section (in part)
2(1) Vertex with few narrow decumbent scales restricted to median area; lateral patch of broad decumbent scales large, evident in dorsal view…3
– Vertex with numerous narrow decumbent scales; lateral patch of broad decumbent scales small, almost indistinct in dorsal view…Spissipes Section (in part)
3(2) Thoracic pleural integument yellowish or lighter, contrasting with brown scutal integument…Spissipes Section
– Thoracic pleural integument similar in color or lighter, not contrast sharply with scutal integument…4
4(3) Thoracic pleural integument lighter than scutal integument, with pattern of dark and pale areas on mesepimeron and mesokatepisternum; upper mesokatepisternum with a patch of white scales; legs with conspicuous or inconspicuous ring of white scales at all femur-tibia joints…Atratus Group (Melanoconion Section)
– Thoracic pleural integument without pattern of dark and pale areas; upper mesokatepisternum without or with a small patch or a few white scales; legs with or without ring or patch of white scales at femur-tibia joints…Other Groups (Melanoconion Section)

**Atratus Group**

1. Proboscis with distinct dorsal and median patches of whitish scales; fore- and midfemur with preapical ring of whitish scales (Fig. 19a)…2
2. Proboscis dark-scaled or with indistinct patch of pale scales; fore- and midfemur without preapical ring of whitish scales (Fig. 19b)…3

**Fig. 19:**
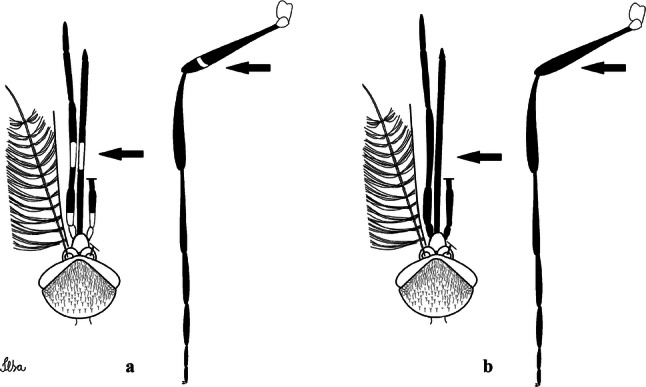
Couplet 1: head and leg features. Arrows indicate the colour pattern of scales on the proboscis and femura. **a** Thesis. **b** Antithesis
3. Costa (C) dark-scaled; vein R entirely dark-scaled. Male: palpomere I pale-scaled; palpomere III with long, distinct median patch of whitish scales on dorsal surface (Fig. 20a)…*Cx*. *caribeanus*
4. Costa (C) with proximal patch of whitish scales; vein R with a long line of whitish scales proximally. Male: palpomere I entirely dark-scaled; palpomere III with small, basal patch of whitish scales (Fig. 20b)…*Cx*. *trigeminatus*

**Fig. 20:**
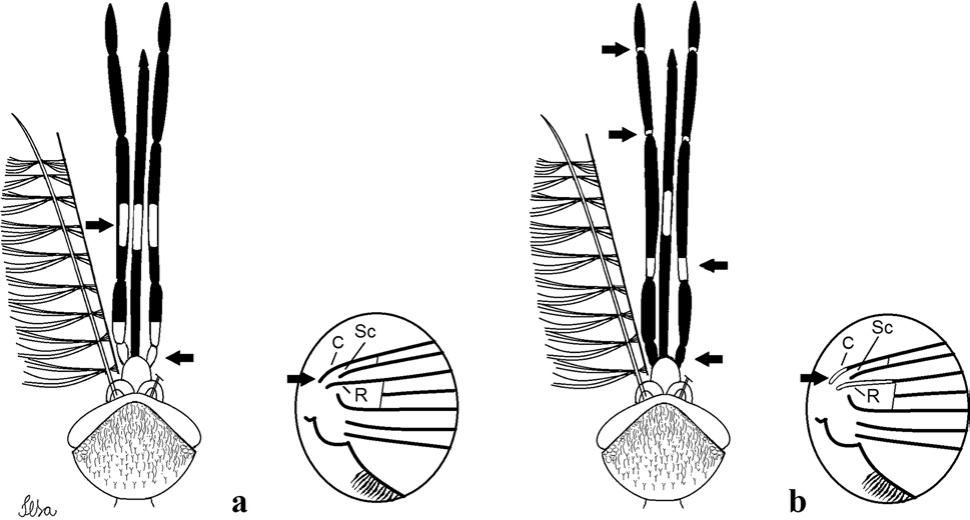
Couplet 2: head features and wing scale patterns. Arrows indicate the colour pattern of scales of the palpomeres and wings. **a** Thesis: *Culex caribeanus*. **b** Antithesis: *Culex trigeminatus*. *Abbreviations*: C, costa; R, radius; Sc, subcosta
5. Proboscis with indistinct dorsomedian patches of whitish scales; costa (C) with inconspicuous proximal patch of whitish scales; vein R with 1 long basal patch of whitish scales. Male: palpomere III with inconspicuous proximal patch of whitish scales (Fig. 21a)…*Cx*. *spinifer* n. sp.
6. Proboscis without dorsal patch of whitish scales; vein R dark-scaled or with 2 basal patches of whitish scales separated by a dark scale-patch (Fig. 21b)…4

**Fig. 21:**
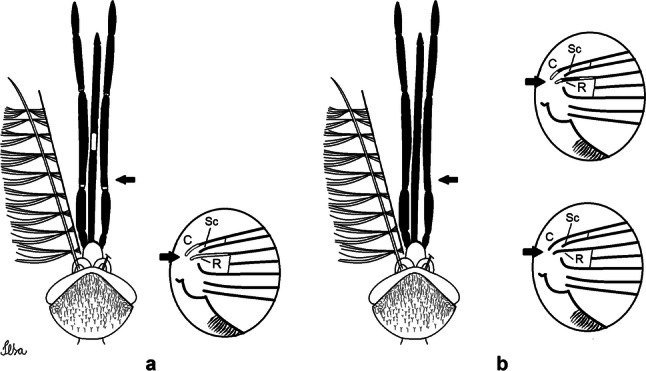
Couplet 3: head features and wing scale patterns. Arrows indicate the colour pattern of scales of the proboscis, palpomeres and wings. **a** Thesis: *Culex spinifer* n. sp. **b** Antithesis. *Abbreviations*: C, costa; R, radius; Sc, subcosta
7. Vein R with 2 basal whitish patches separated by dark-scaled patch (Fig. 22a)…5
8. Vein R entirely dark-scaled (Fig. 22b)…7

**Fig. 22:**
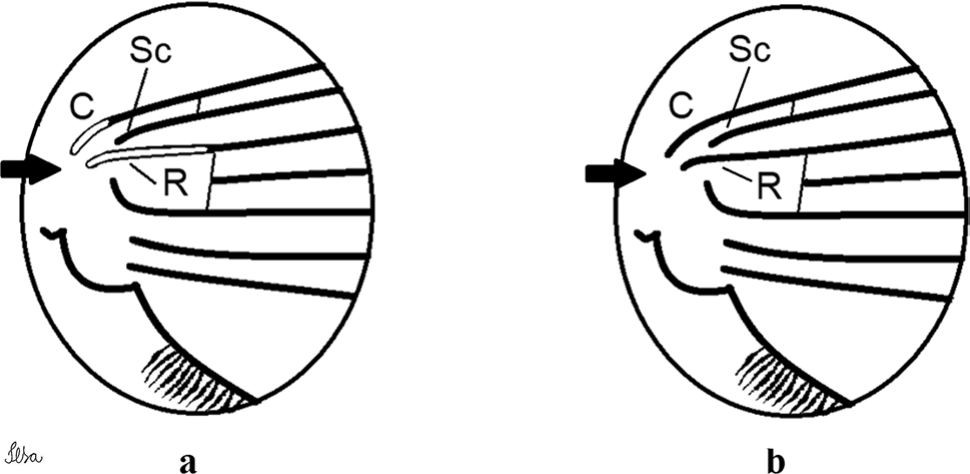
Couplet 4: wing scale patterns. Arrows indicate the colour pattern of wing scales. **a** Thesis. **b** Antithesis. *Abbreviations*: C, costa; R, radius; Sc, subcosta
9. Scutal integument dark brown/black with whitish and golden scales (as indicated by white lines in Fig. 23a) and dark scales (other areas) forming distinct ornamentation pattern. Male: palpomere III without whitish-scaled patch…6
10. Scutal integument dark brown with golden scales without distinct ornamentation pattern (Fig. 23b). Male: palpomere III with indistinct basal whitish-scaled patch…*Cx*. *dunni*/*Cx*. *exedrus*

**Fig. 23:**
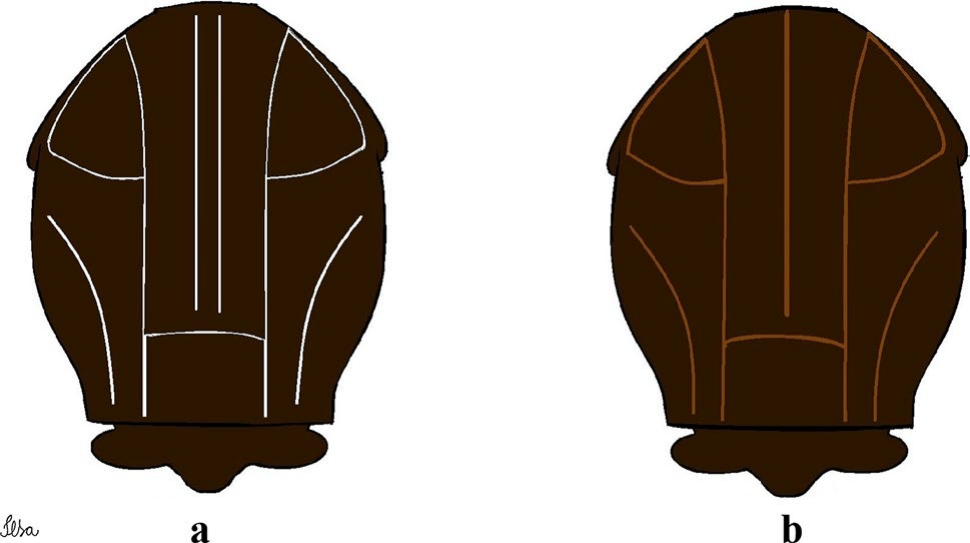
Couplet 5: ornamentation pattern of scutal scales. **a** Thesis. **b** Antithesis: *Culex dunni*/*Culex exedrus*
11. Halter with capitellum brownish (Fig. 24a); subcosta (Sc) with indistinct proximal patch of whitish scales; vein R with 2 proximal whitish patches separated by median dark-scaled patch (Fig. 24b)…*Cx*. *comptus* n. sp.
12. Halter with capitellum whitish (Fig. 24c); subcosta (Sc) without whitish patch; vein R with 2 basal whitish patches separated by small dark-scaled patch (Fig. 24d)…*Cx*. *ensiformis*

**Fig. 24:**
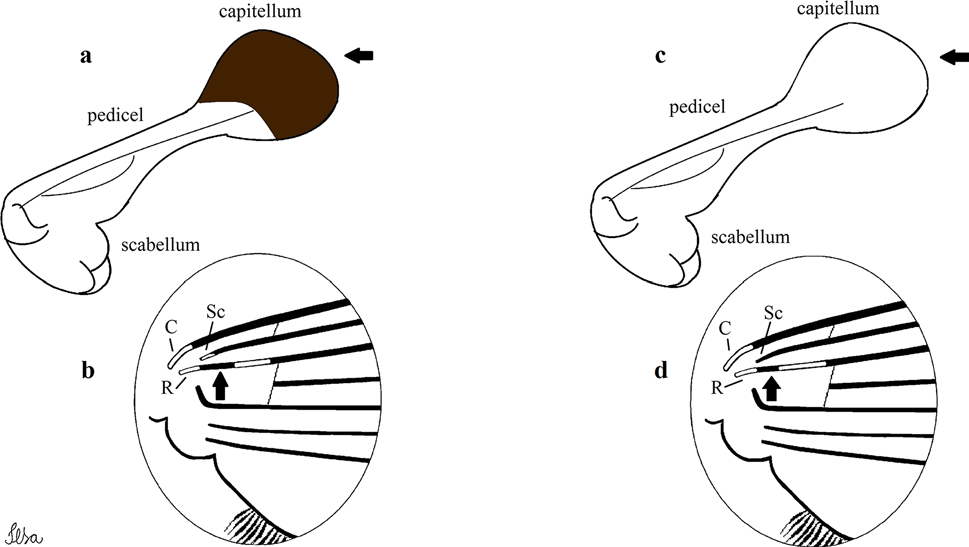
Couplet 6: halter colour and wing scale patterns. Arrows indicate the colour of capitellum integument and the colour pattern of wing scales. **a**, **b** Thesis: *Culex comptus* n. sp., capitellum colour (**a**) and wing scale patterns (**b**). **c**, **d** Antithesis: *Culex ensiformis*, capitellum colour (**c**) and wing scale patterns (**d**). *Abbreviations*: C, costa; R, radius; Sc, subcosta
13. Upper corner of mesokatepisternum (Mkm) with patch of broad whitish scales as long as the mesothoracic spiracle (MS) (Fig. 25a)…8
14. Upper corner of mesokatepisternum (Mkm) with patch of broad whitish scales shorter than the mesothoracic spiracle (MS) (Fig. 25b)…9

**Fig. 25:**
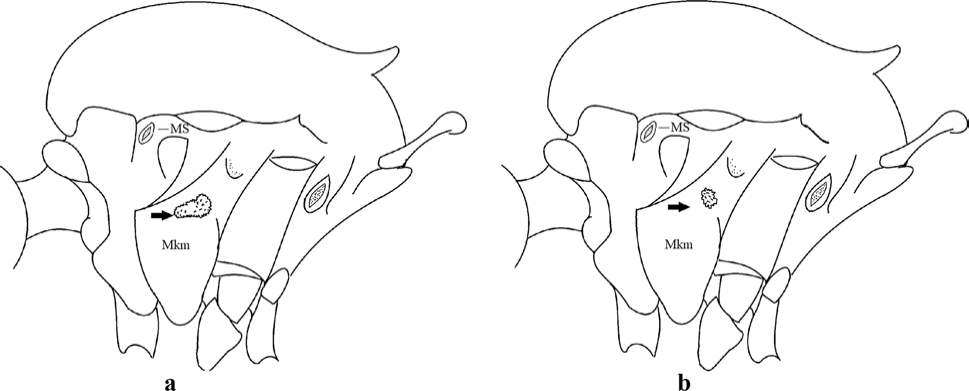
Couplet 7: thoracic pleura. Arrows indicate patches of white scales on upper mesokatepisternum. Adapted from Forattini (1996) [82]. **a** Thesis. **b** Antithesis. *Abbreviations*: Mks, mesokatepisternum; MS, mesothoracic spiracle
15. Costa entirely dark-scaled (Fig. 26a). Male: palpomere III with indistinct basal whitish scale-patch (Fig. 26b)…………………………*Cx*. *longisetosus* n. sp.
16. Costa with proximal whitish scale-patch (Fig. 26c). Male: palpomere III without basal whitish scale-patch (Fig. 26d)…*Cx*. *longistylus* n. sp.

**Fig. 26:**
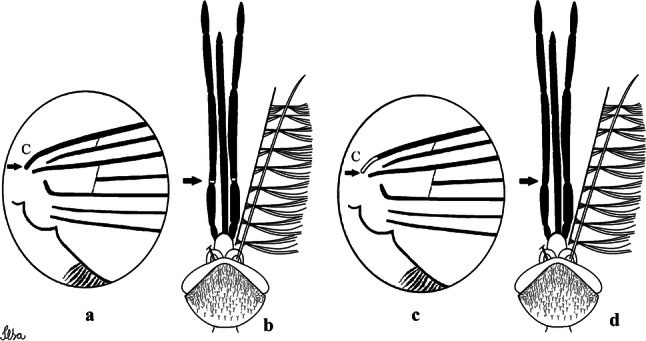
Couplet 8: head features and wing scale patterns. Arrows indicate the colour pattern of scales of palpomere III and wings. **a**, **b** Thesis: *Culex longisetosus* n. sp., costa vein (**a**) and male palpomere III (**b**). **c**, **d** Antithesis: *Culex longistylus* n. sp., costa vein (**c**) and male palpomere III (**d**). *Abbreviation*: C, costa
17. Mesepimeral integument (Mm) dark with distinct median whitish area completely separating darker upper and lower areas (Fig. 27a)…*Cx*. *zeteki* / *Cx*. *loturus*
18. Mesepimeral integument (Mm) entirely dark or with indistinct median whitish area not completely separating the dark areas (Fig. 27b)…10

**Fig. 27:**
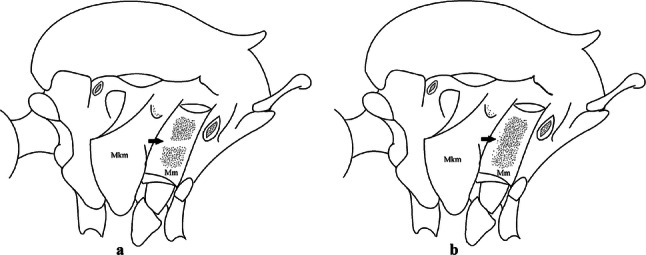
Couplet 9: thoracic pleura. Arrows indicate the mesepimeral integument. Adapted from Forattini (1996) [82]. **a** Thesis: *Culex zeteki*. **b** Antithesis. *Abbreviations*: Mkm, mesokatepisternum. Mm, mesepimeron
19. Mesepimeral integument (Mm) dark, without median pale area; terga II-VIII (II-VIII-Te) with basolateral patches of whitish scales, without basal pale bands (Fig. 28a)…*Cx*. *atratus*
20. Mesepimeral integument (Mm) dark, with indistinct median pale area that does not completely separate the dark area; terga II-VIII (II-VIII-Te) with pale basal bands (Fig. 28b)…*Cx*. *columnaris* n. sp.

**Fig. 28:**
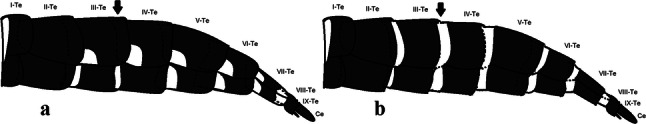
Couplet 10: abdomen. Arrows indicate the scale pattern of terga. Adapted from Harbach & Knight (1980) [25]. **a** Thesis: *Culex atratus*. **b** Antithesis: *Culex columnaris* n. sp. *Abbreviations*: I-Te, tergum I; II-Te, tergum II; III-Te, tergum III; IV-Te, tergum IV; V-Te, tergum V; VI-Te, tergum VI; VII-Te, tergum VII; VIII-Te, tergum VIII; IX-Te, tergum IX; Ce, cerca
21. ** Keys based on the morphology of male genitalia**
22. Aedeagal sclerite (AeS) broad and curved in lateral view, largely connected to dorsal process of lateral plate (LP)…Spissipes Section
23. Aedeagal sclerite (AeS) narrow and curved in lateral view, lightly connected to dorsal process of lateral plate (LP)…2
24. Gonocoxite (Gc) small, narrow, oblong; gonostylus (Gs) narrow, dorsal area without setae; lateral plate (LP) with concave ventral process and pointed apical process…Atratus Group (Melanoconion Section)
25. Gonocoxite (Gc) conical, ovoid or globose; gonostylus variously modified; lateral plate (LP) with ventral and lateral processes otherwise modified…Other Groups (Melanoconion Section)

**Atratus Group**

1. Proximal division of subapical lobe (pSL) without columnar process, setae only inserted on tubercles or directly inserted on surface of the gonocoxite (Fig. 29a)…2
2. Proximal division of subapical lobe (pSL) with long columnar process divided apically (Fig. 29b)…*Cx*. *columnaris* n. sp.

**Fig. 29:**
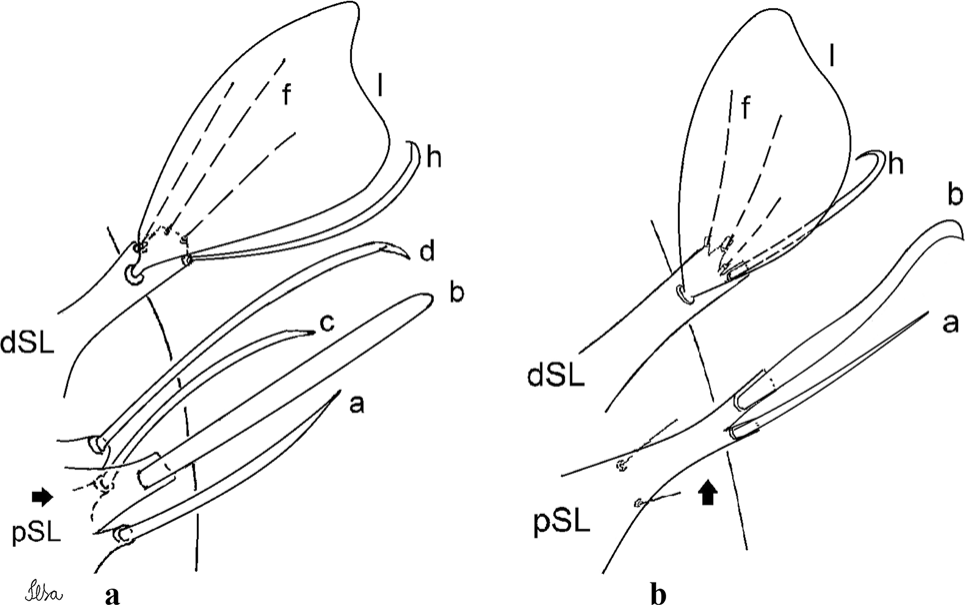
Couplet 1: setae on the subapical lobe of the gonocoxite. Arrows indicate the proximal division of the subapical lobe. **a** Thesis. **b** Antithesis: *Culex columnaris* n. sp. *Abbreviations*: a, seta *a*; b, seta *b*; c, seta *c*; d, seta *d*; f, setae *f*; h, seta *h*; l, seta *l*; pSL, proximal division of the subapical lobe; dSL, distal division of the subapical lobe
3. Proximal division of subapical lobe (pSL) with 2 setae (Fig. 30a)…3
4. Proximal division of subapical lobe (pSL) with 3 or more setae (Fig. 30b)…8

**Fig. 30:**
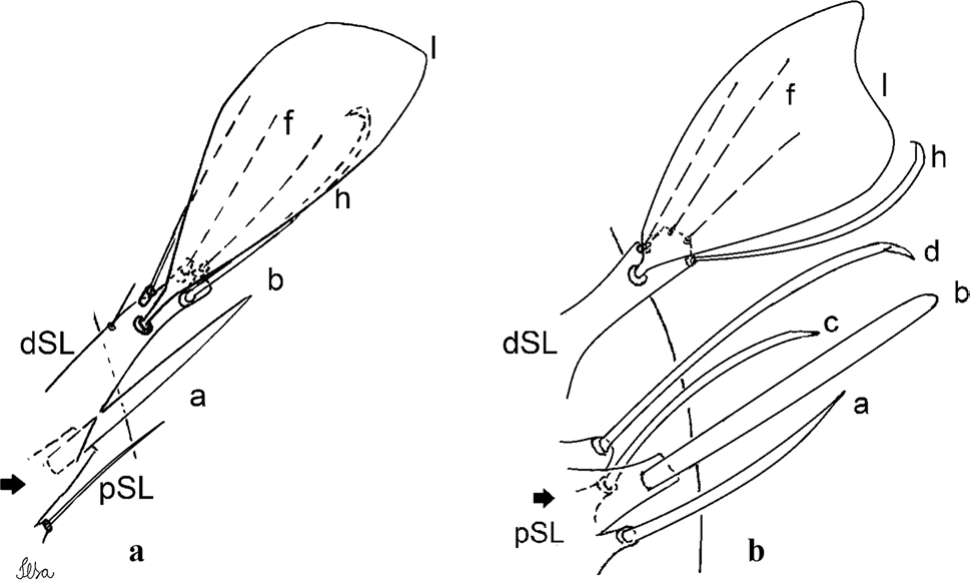
Couplet 2: setae on subapical lobe of the gonocoxite. Arrows indicate setae of the proximal division of the subapical lobe. **a** Thesis. **b** Antithesis. *Abbreviations*: a, seta *a*; b, seta *b*; c, seta *c*; d, seta *d*; f, setae *f*; h, seta *h*; l, seta *l*; pSL, proximal division of the subapical lobe; dSL, distal division of the subapical lobe
5. Distal division of subapical lobe (dSL) with a short (length less than half length of seta *b* of proximal division) columnar process (Fig. 31a)…4
6. Distal division of subapical lobe (dSL) with a long (length greater than or equal to half length of seta *b* of proximal division) columnar process (Fig. 31b)…6

**Fig. 31:**
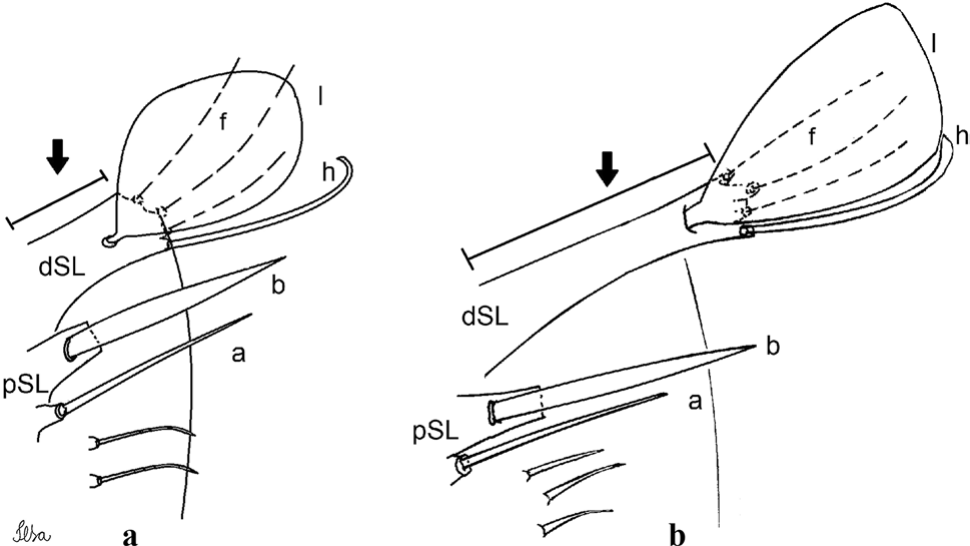
Couplet 3: setae on the subapical lobe of the gonocoxite. Arrows indicate the distal division of the subapical lobe. **a** Thesis. **b** Antithesis. *Abbreviations*: a, seta *a*; b, Seta *b*; f, Seta *f*; h, Seta *h*; l, Seta *l*; pSL, proximal division of the subapical lobe; dSL, distal division of the subapical lobe
7. Distal division (dSL) with seta *l* short (length less than seta *h* length) (Fig. 32a); tergum IX lobe (IX-TL) longer than wide, with apically bifid and/or simple setae on median portion (Fig. 32b); gonocoxite with slender and short setae on ventromesal surface (Fig. 32c)…5
8. Distal division (dSL) with seta *l* long (with length greater than or equal to seta *h* length) (Fig. 32d); tergum IX (IX-TL) lobe pear-shaped (Fig. 32e); gonocoxite with 4 or 5 broad spatulate setae on ventromesal surface (Fig. 32f)…*Cx*. *atratus*

**Fig. 32:**
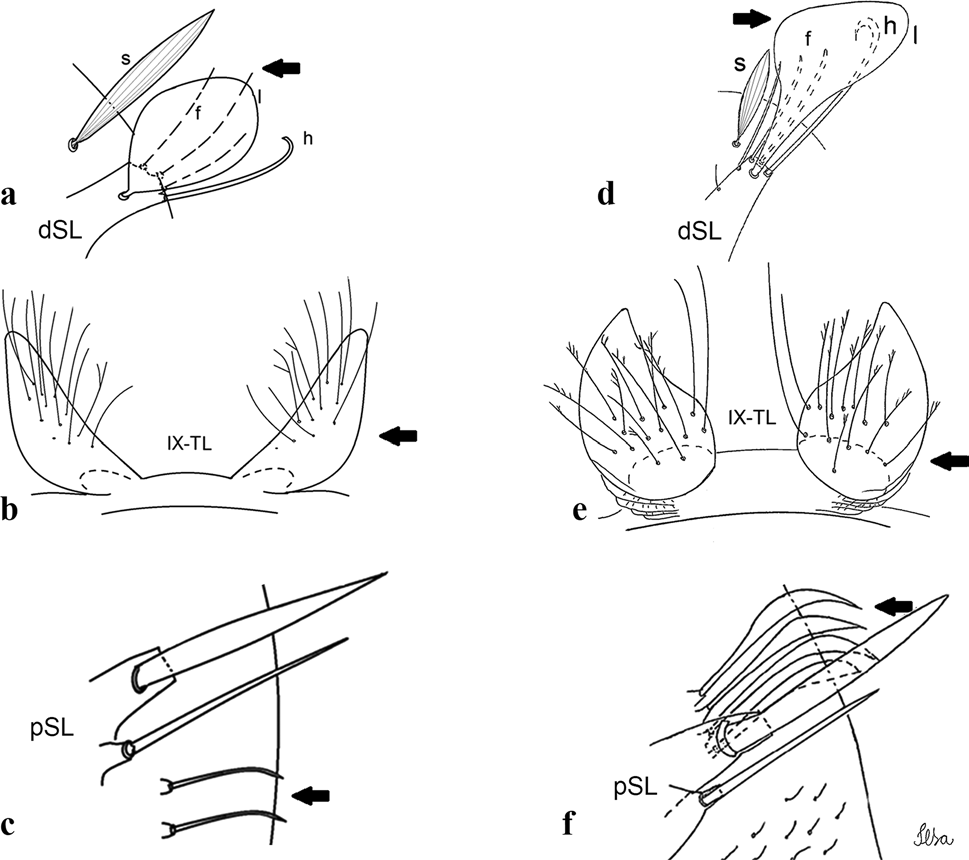
Couplet 4: setae on the subapical lobe of the gonocoxite and ninth tergal lobe. Arrows indicate the distal division of the subapical lobe, ninth tergal lobe and ventromesal surface of the gonocoxite. **a-c** Thesis: seta *l* (**a**), ninth tergal lobe (**b**) and setae on ventralmesal surface of gonocoxite (**c**). **d-f** Antithesis: *Culex atratus*, seta *l* (**d**), ninth tergal lobe (**e**) and ventromesal surface of gonocoxite (**f**). *Abbreviations*: f, setae *f*; h, seta *h*; l, seta *l*; IX-TL, ninth tergal lobe; pSL, proximal division of the subapical lobe; dSL, distal division of the subapical lobe
9. Tergum IX lobe (IX-TL) with simple setae (Fig. 33a); aedeagal sclerite (AeS) with numerous and conspicuous ventral spicules (Fig. 33b); gonostylus (Gs) with a slender gonostylar claw (GC) (Fig. 33c)…*Cx*. *ensiformis*
10. Tergum IX lobe (IX-TL) with simple and apically bifid setae (Fig. 33d); aedeagal sclerite (AeS) without ventral spicules (Fig. 33e); gonostylus (Gs) with a broad gonostylar claw (GC) (Fig. 33f)…*Cx*. *spinifer* n. sp.

**Fig. 33:**
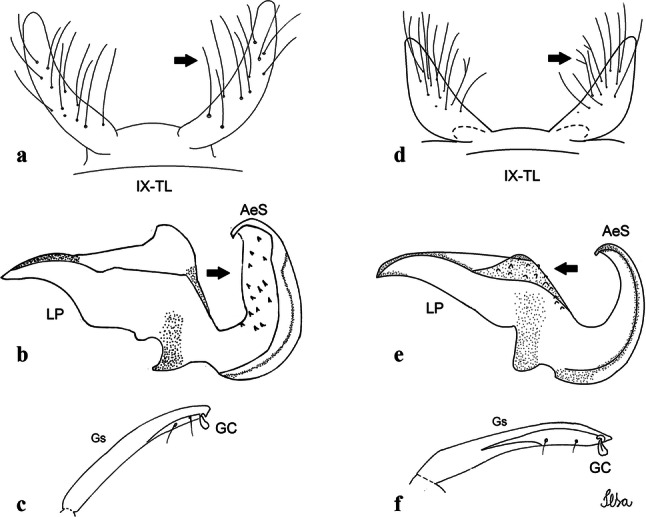
Couplet 5: ninth tergal lobes, aedeagus and gonostylus. Arrows indicate setae of the ninth tergal lobe, aedeagal sclerite, lateral plate and gonostylar claw. **a–c** Thesis: *Culex ensiformis*, setae of the ninth tergal lobe (**a**), ventral spicules on aedeagal sclerite (**b**) and gonostylar claw (**c**). **d-f** Antithesis: *Culex spinifer* n. sp., setae of the ninth tergal lobe (**d**), ventral spicules on lateral plate (**e**) and gonostylar claw (**f**). *Abbreviations:* IX-TL, ninth tergal lobe; Aes, aedeagal sclerite; LP, lateral plate; Gs, gonostylus; GC, gonostylar claw
11. Tergum IX lobe (IX-TL) elongate, longer than wide, with only simple setae or simple and few apically bifid setae (Fig. 34a)…7
12. Tergum IX lobe (IX-TL) somewhat rounded, wider than long, with few simple and several apically bifid setae (Fig. 34b)…*Cx*. *longistylus* n. sp.

**Fig. 34:**
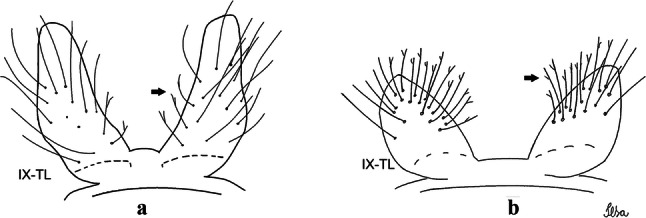
Couplet 6: ninth tergal lobes. Arrows indicate setae of the ninth tergal lobe. **a** Thesis. **b** Antithesis: *Culex longistylus* n. sp. *Abbreviations*: IX-TL, ninth tergal lobe
13. Tergum IX lobe (IX-TL) slender, with simple setae (Fig. 35a); aedeagal sclerite (AeS) with few indistinct ventral spicules (Fig. 35b); gonocoxite (Gc) with slender, short setae on ventromesal surface (Fig. 35c)…*Cx*. *comptus* n. sp.
14. Tergum IX lobe (IX-TL) slightly broad, with simple, apically bifid setae (Fig. 35d); aedeagal sclerite (AeS) without ventral spicules (Fig. 35e); gonocoxite (Gc) with 4 long, spatulate setae on ventromesal surface (Fig. 35f)…*Cx*. *longisetosus* n. sp.

**Fig. 35:**
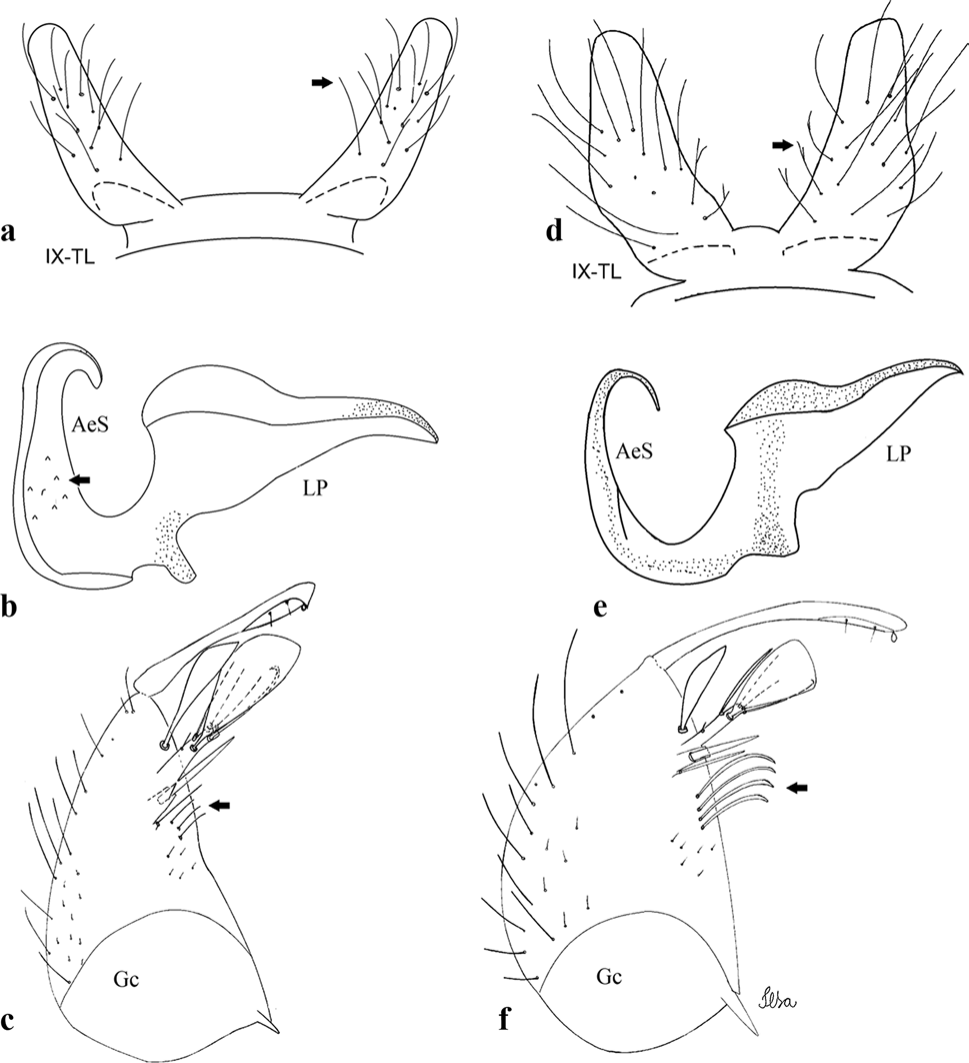
Couplet 7: ninth tergal lobe, aedeagus and gonocoxite. Arrows indicate setae of the ninth tergal lobe, aedeagal sclerite, and gonocoxite. **a-c** Thesis: *Culex comptus* n. sp., ninth tergal lobe (**a**), ventral spicules on aedeagal sclerite (**b**) and ventromesal surface of gonocoxite (**c**). **d–f** Antithesis: *Culex longisetosus* n. sp., ninth tergal lobe (**d**), aedeagal sclerite (**e**) and ventromesal surface of gonocoxite (**f**). *Abbreviations*: IX-TL, ninth tergal lobe; Aes, aedeagal sclerite; LP, lateral plate; Gc, gonocoxite
15. Lateral plate (LP) of aedeagus with spicules on ventral process (Fig. 36a); tergum X (X-Te) elongate and sinuous (Fig. 36b)…9
16. Lateral plate (LP) of aedeagus without spicules on ventral process (Fig. 36c); tergum X (X-Te) somewhat triangular or irregular in outline, with rounded prominence (Fig. 36d)…10

**Fig. 36:**
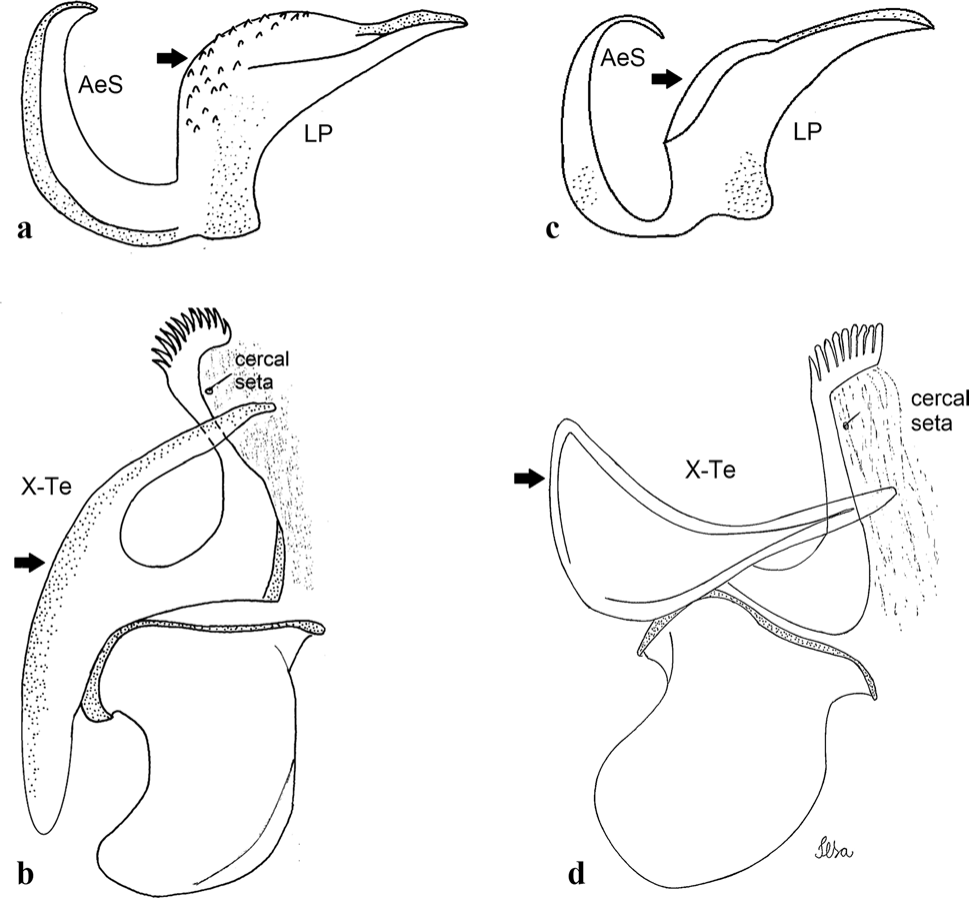
Couplet 8: aedeagus and proctiger. Arrows indicate the lateral plate and tergum X. **a**, **b** Thesis. **c**, **d** Antithesis. *Abbreviations*: Aes, aedeagal sclerite; LP, lateral plate; X-Te, tergum X
17. Gonocoxite (Gc) with long and dispersed setae on sternomesal surface (Fig. 37a); tergum X (X-Te) with long, slender inner process (Fig. 37b)…*Cx*. *dunni*
18. Gonocoxite (Gc) with long and aligned setae on sternomesal surface (Fig. 37c); tergum X (X-Te) with long, broader inner process (Fig. 37d)…*Cx*. *exedrus*

**Fig. 37:**
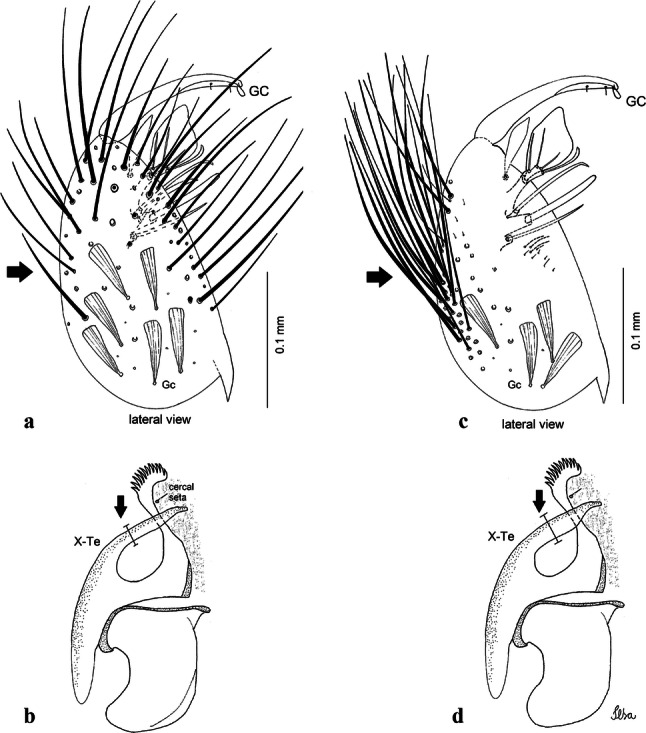
Couplet 9: gonocoxite and proctiger. Arrows indicate setae on the sternomesal surface and tergum X. **a**, **b** Thesis: *Culex dunni*, gonocoxite in lateral view (**a**) and thickness of the inner process of tergum X (**b**). **c**, **d** Antithesis: *Culex exedrus*, gonocoxite in lateral view (**c**) and thickness of the inner process of tergum X (**d**). *Abbreviations*: Gc, gonocoxite; GC, gonostylar claw; X-Te, tergum X
19. Proximal division of subapical lobe (pSL) with 3 setae (Fig. 38a); apical process of lateral plate (LP) of aedeagus with distinct or indistinct ripples on apical-median process; ventral process of lateral plate without pointed projection in lateral view (Fig. 38b)…11
20. Proximal division of subapical lobe (pSL) with 4 setae (Fig. 38c); lateral plate (LP) of aedeagus without ripples; ventral process of lateral plate with pointed projection in lateral view (Fig. 38d)…12

**Fig. 38:**
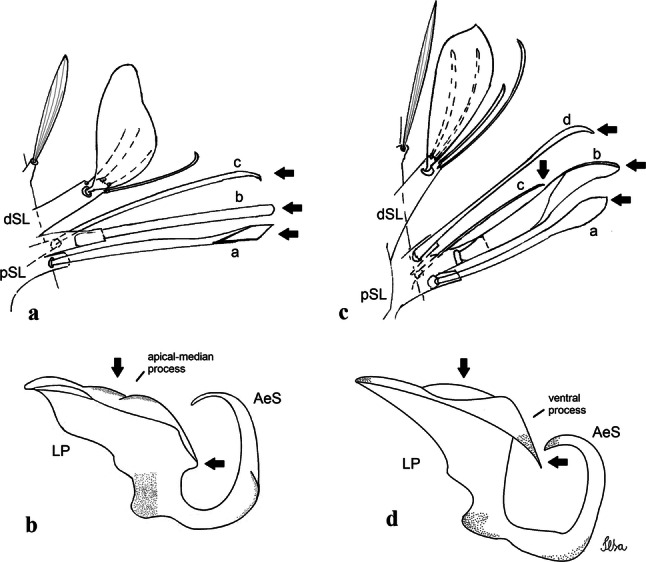
Couplet 10: subapical lobe of the gonocoxite and aedeagus. Arrows indicate the proximal division of the subapical lobe and lateral plate. **a**, **b** Thesis. **c, d** Antithesis. *Abbreviations*: a, seta *a*; b, seta *b*; c, seta *c*; Aes, aedeagal sclerite; LP, lateral plate; pSL, proximal division of the subapical lobe; dSL, distal division of the subapical lobe
21. Lateral plate (LP) of aedeagus with distinct ripples on apical process (Fig. 39a); seta *l* of distal division of subapical lobe (dSL) with rounded and laterally directed apex (Fig. 39b); gonostylus (Gs) with 3 or 4 conspicuous folds on dorsal surface (Fig. 39c)…*Cx*. *zeteki*
22. Lateral plate (LP) of aedeagus with indistinct ripples on apical process (Fig. 39d); seta *l* of distal division of subapical lobe (dSL) with pointed and laterally directed apex (Fig. 39e); gonostylus (Gs) with inconspicuous folds on dorsal surface (Fig. 39f)…*Cx*. *loturus*

**Fig. 39:**
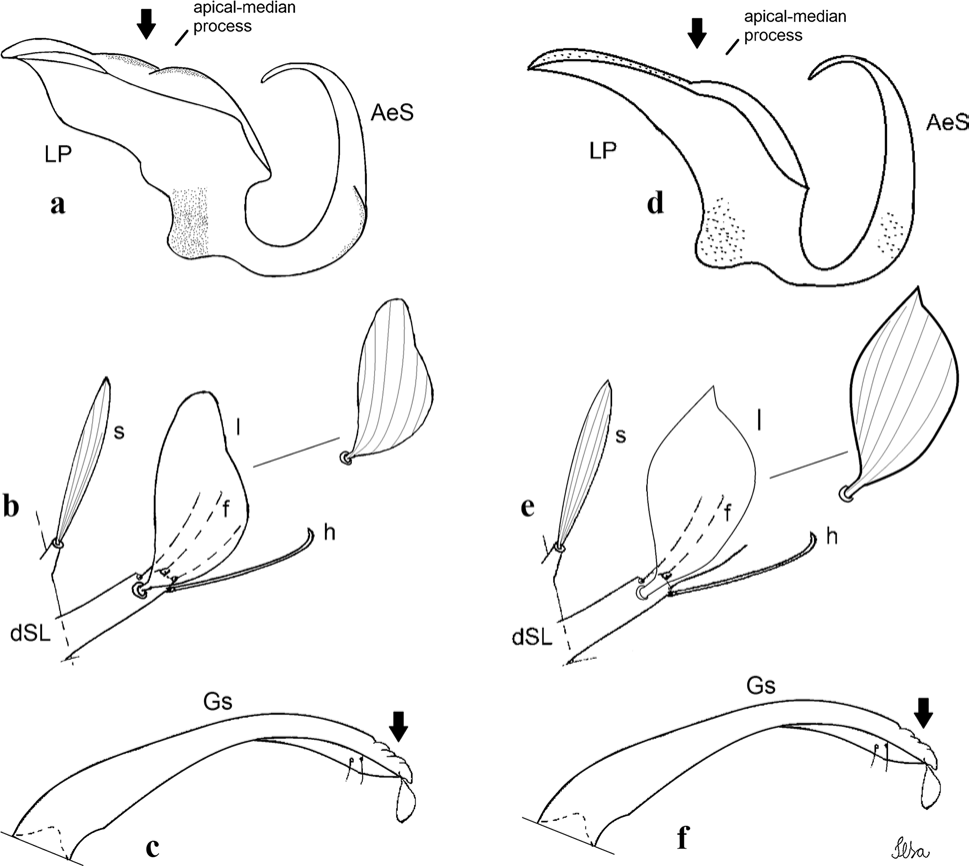
Couplet 11: aedeagus, subapical lobe of gonocoxite and gonostylus. Arrows indicate the lateral plate, distal division of the subapical lobe and gonostylus. **a–c** Thesis: *Culex zeteki*, apical-median process of the lateral plate (**a**), seta *l* of the distal division of the subapical lobe (**b**) and dorsal surface of gonostylus (**c**). **d–f** Antithesis: *Culex loturus*, apical-median process of the lateral plate (**d**), seta *l* of the distal division of subapical lobe (**e**) and dorsal surface of gonostylus (**f**). *Abbreviations*: dSL, distal division of the subapical lobe; f, seta *f*; h, seta *h*; l, seta *l*; s, seta *s*; Aes, aedeagal sclerite; LP, lateral plate; Gs, gonostylus
23. Seta *l* of distal division of subapical lobe (dSL) with rounded apex; seta *b* of proximal division of subapical lobe (pSL) long, slender, apically sinuous (Fig. 40a); lateral plate (LP) of aedeagus with large pointed projection directed ventrobasally in lateral view (Fig. 40b); tergum IX lobe (IX-TL) with aciculate setae (Fig. 40c)…*Cx*. *caribeanus*
24. Seta *l* of distal division of subapical lobe (dSL) with pointed apex; seta *b* of proximal division of subapical lobe (pSL) long and stout (Fig. 40d); lateral plate (LP) of aedeagus with small pointed projection directed ventrobasally in lateral vew (Fig. 40e); tergum IX lobe (IX-TL) with simple setae (Fig. 40f)…*Cx*. *trigeminatus*

**Fig. 40:**
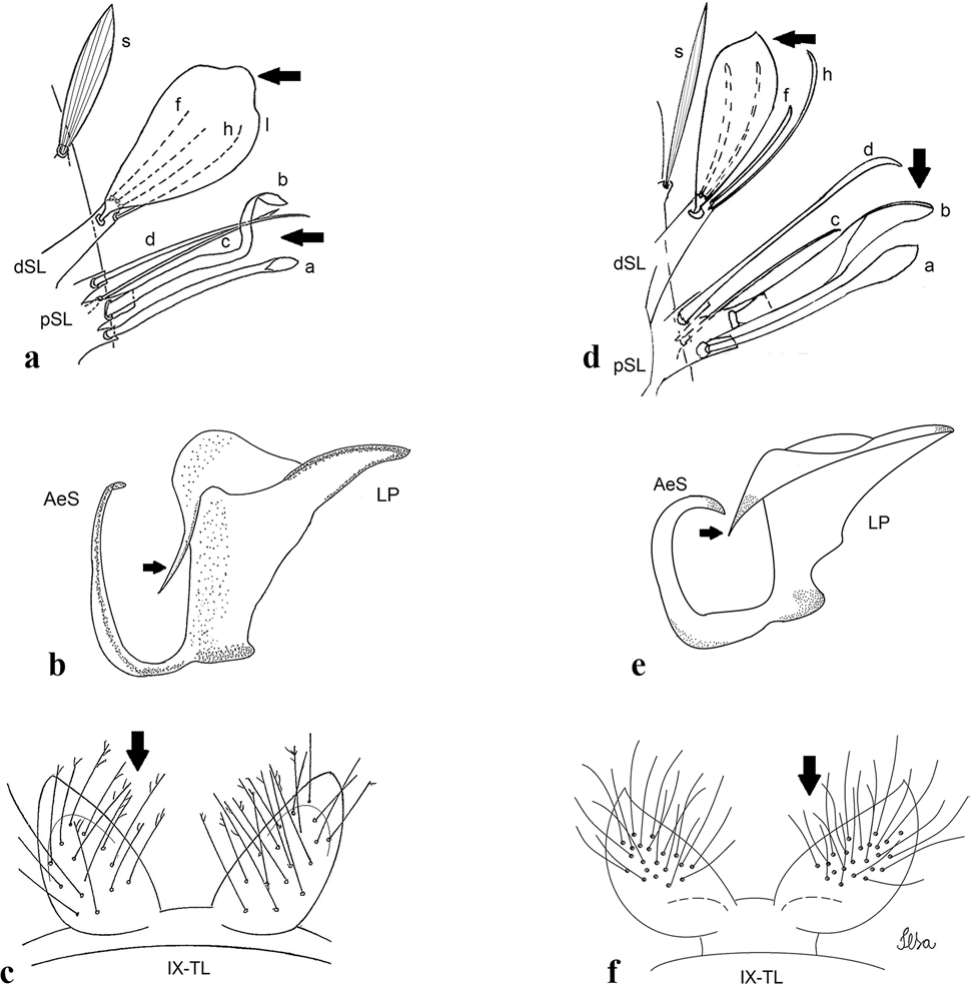
Couplet 12: subapical lobe of the gonocoxite, aedeagus and ninth tergal lobes. Arrows indicate setae of the subapical lobe, lateral plate and ninth tergal lobe. **a–c** Thesis: *Culex caribeanus*, setae of the proximal and distal divisions of the subapical lobe (**a**), lateral plate in lateral view (**b**) and ninth tergal lobe (**c**). **d–f** Antithesis: *Culex trigeminatus*, setae of the proximal and distal divisions of the subapical lobe (**d**), lateral plate in lateral view (**e**) and ninth tergal lobe (**f**). *Abbreviations*: a, seta *a*; b, seta *b*; c, seta *c*; d, seta *d*; f, seta *f*; h, seta *h*; l, seta *l*; s, seta *s*; Aes, aedeagal sclerite; LP, lateral plate; IX-TL, ninth tergal lobe; pSL, proximal division of the subapical lobe; dSL, distal division of the subapical lobe

(iii) **Keys based on pupal morphology**
1 Seta 9-VIII inserted at or near to caudolateral margin; caudolateral angle of segment VIII blunt…Spissipes Section
– Seta 9-VIII inserted above of caudolateral margin; caudolateral angle of segment VIII slightly pointed…2
2(1) Trumpet narrow, long, length/width ratio 7 to 30…Atratus Group (Melanoconion Section)
– Trumpet thick, of shorter length, length/width ratio 5 to 8…Other Groups (Melanoconion Section)

**Atratus Group**

1. Trumpet (T) with distal margin of pinna (Pi) without emargination; if present, emargination indistinct (Fig. 41a)…2
2. Trumpet (T) with distal margin of pinna with conspicuous emargination (Fig. 41b)…4

**Fig. 41:**
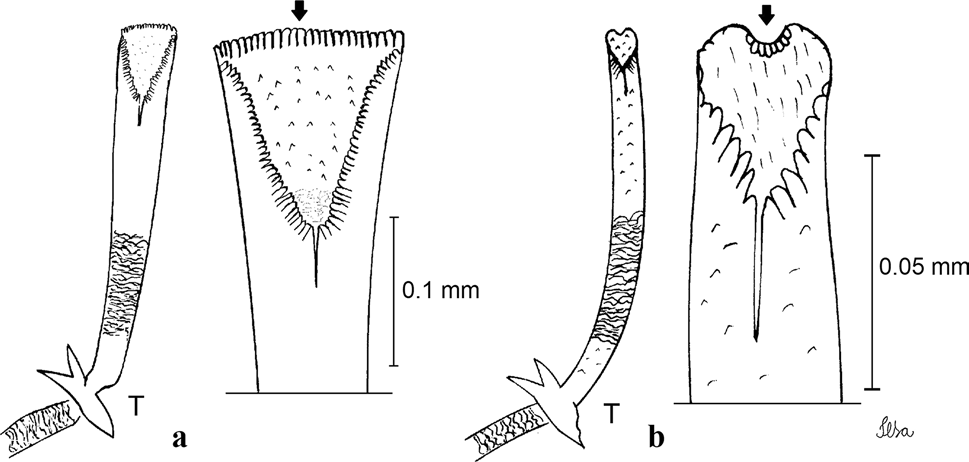
Couplet 1: trumpet and pinna. Arrows indicate the distal margin of pinna. **a** Thesis. **b** Antithesis. *Abbreviation*: T, trumpet
3. Trumpet (T) with a V-shaped pinna (Pi); trumpet large, wider at apex than at base (Fig. 42a); trumpet index 7–10; seta 9-VIII with 4 aciculate branches (Fig. 42b)…*Cx*. *atratus*
4. Pinna (Pi) small, not V-shaped; trumpet narrow from base to apex (Fig. 42c); trumpet index > 15; seta 9-VIII with simple branches (Fig. 42d)…3

**Fig. 42:**
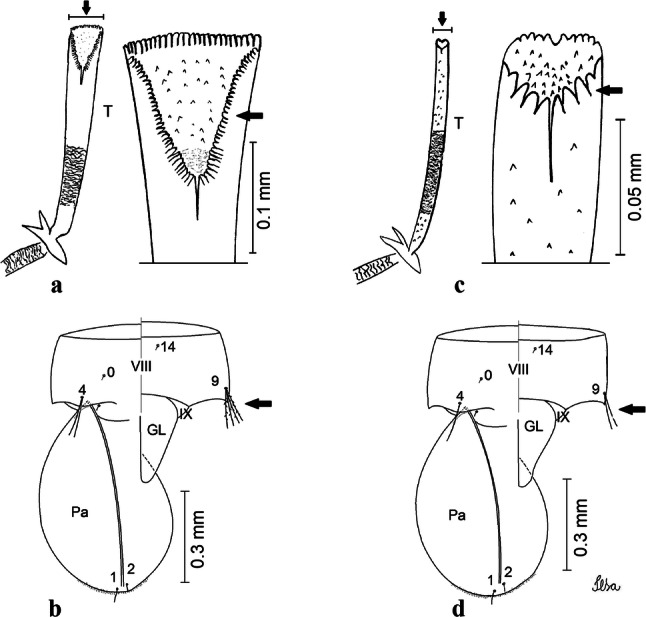
Couplet 2: trumpet, pinna and abdominal segment VIII. Arrows indicate trumpet, pinna and segment VIII. **a**, **b** Thesis: *Culex atratus*, trumpet and pinna shape (**a**) and seta 9 of abdominal segment VIII (**b**). **c**, **d** Antithesis. *Abbreviations*: T, trumpet; VIII, abdominal segment VIII; GL, genital lobe; Pa, paddle
5. Trumpet index 20; pinna (Pi) short, somewhat rounded, meatal cleft (MC) short; distal margin opposite meatal cleft with small notch (Fig. 43a)…*Cx*. *trigeminatus*
6. Trumpet index 17; pinna (Pi) heart-shaped in lateral view, becoming slender at base, meatal cleft (MC) long; distal margin opposite meatal cleft with small, rounded emargination (Fig. 43b)…*Cx*. *zeteki*

**Fig. 43:**
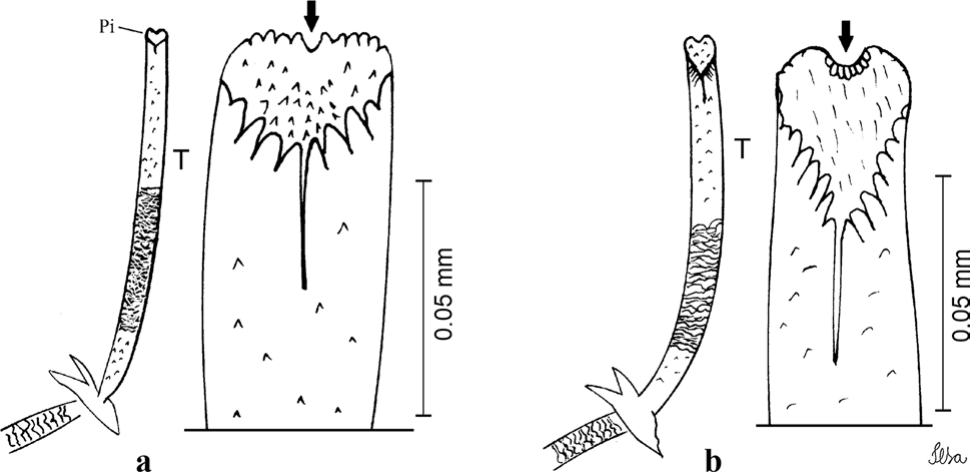
Couplet 3: trumpet and pinna. Arrows indicate the distal margin of pinna. **a** Thesis: *Culex trigeminatus*. **b** Antithesis: *Culex zeteki*. *Abbreviation*: T, trumpet
7. Trumpet (T) long; pinna (Pi) cup-shaped in lateral view; trumpet index > 20; distal margin opposite meatal cleft (MC) with indistinct, shallow transverse depression (Fig. 44a)…*Cx*. *ensiformis*
8. Trumpet (T) moderately long; pinna (Pi) narrow; trumpet index < 16; distal margin opposite meatal cleft with indistinct longitudinal notch (Fig. 44b)…5

**Fig. 44:**
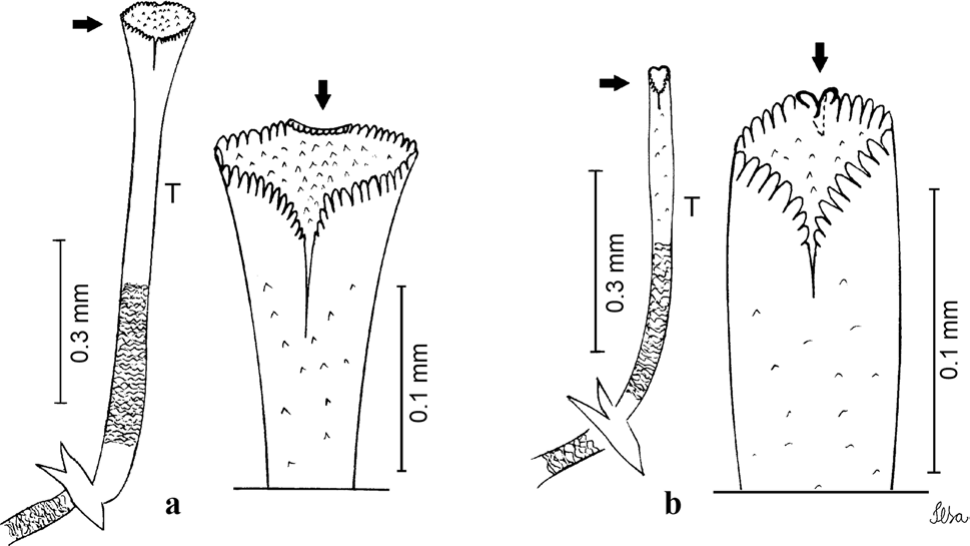
Couplet 4: trumpet and pinna. Arrows indicate the distal margin of pinna. **a** Thesis: *Culex ensiformis*. **b** Antithesis. *Abbreviation*: T, trumpet
9. Trumpet (T) widened distally; distal margin opposite meatal cleft with large emargination (Fig. 45a); trumpet index *c.*14…*Cx*. *dunni*
10. Trumpet (T) narrow; distal margin opposite meatal cleft with indistinct longitudinal fissure (Fig. 45b); trumpet index *c.*15…*Cx*. *comptus* n. sp.

**Fig. 45:**
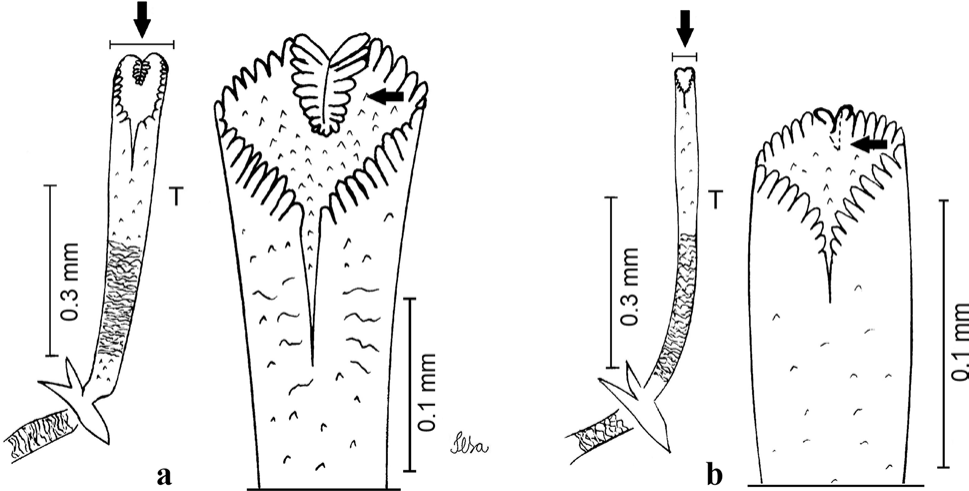
Couplet 5: trumpet and pinna. Arrows indicate the distal margin of pinna. **a** Thesis: *Culex dunni*. **b** Antithesis: *Culex comptus* n. sp. *Abbreviation*: T, trumpet

(iv) **Keys based on fourth-instar larvae morphology**
1 Seta 2-C present; seta 14-C inserted anteriorly to 15-C…Spissipes Section “partim”
– Seta 2-C absent; seta 14-C and 15-C inserted at same level or 14-C slightly anterior to 15-C…2
2(1) Siphon slender and long, index 7–10; seta 1-S with 2–4 short pairs of dorsolateral setae; thoracic and abdominal integument without spicules; segment X with few spicules on posterior margin…Atratus Group (Melanoconion Section)
– Siphon thick and short, index lower than 7; seta 1-S with 2 short or long pairs of dorsolateral setae; thoracic and abdominal integument with distinct or indistinct spicules; segment X with several spicules on posterior margin…Other Groups (Melanoconion Section)

**Atratus Group**

1. Comb scales (CS) of segment VIII different in size and shape: long, pointed and laterally fringed, and shorter apicolaterally fringed scales (Fig. 46a)…2
2. Comb scales (CS) of segment VIII similar in size and shape (Fig. 46b)…3

**Fig. 46:**
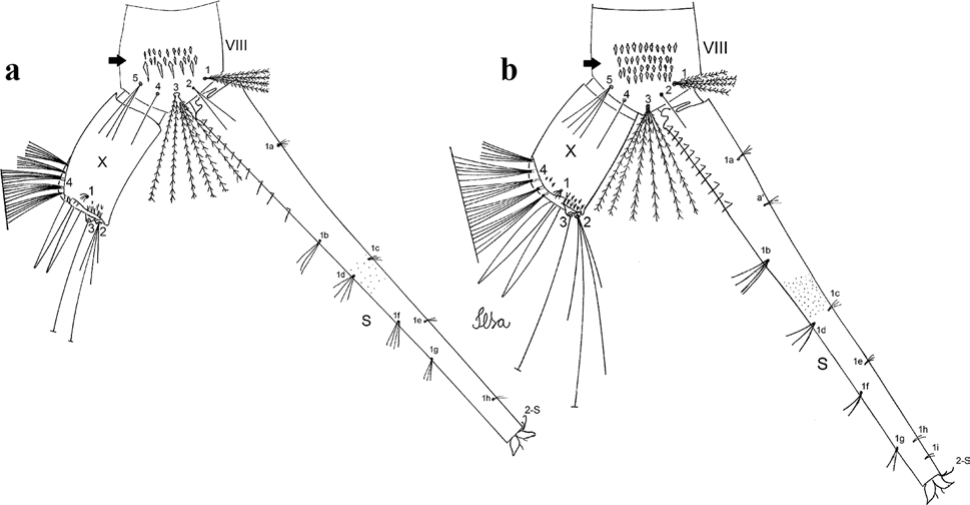
Couplet 1: abdominal segments VIII and X and siphon. Arrows indicate comb scales. **a** Thesis. **b** Antithesis. *Abbreviations*: VIII, abdominal segment VIII; X, VIII, abdominal segment X; S, siphon
3. Seta 5-C with 5 short branches not reaching 6-C insertion; seta 13-C triple (Fig. 47a); siphon (S) with 2 dorsal pairs of seta 1-S (Fig. 47b); pecten spines (PS) elongate, with coarse marginal denticles (Fig. 47c)…*Cx*. *trigeminatus*
4. Seta 5-C with 8 long branches extending beyond 6-C insertion; seta 13-C double (Fig. 47d); siphon (S) with 4 dorsal pairs of seta 1-S (Fig. 47e); pecten spines (PS) long with fine marginal denticles (serration) (Fig. 47f)…*Cx*. *ensiformis*

**Fig. 47:**
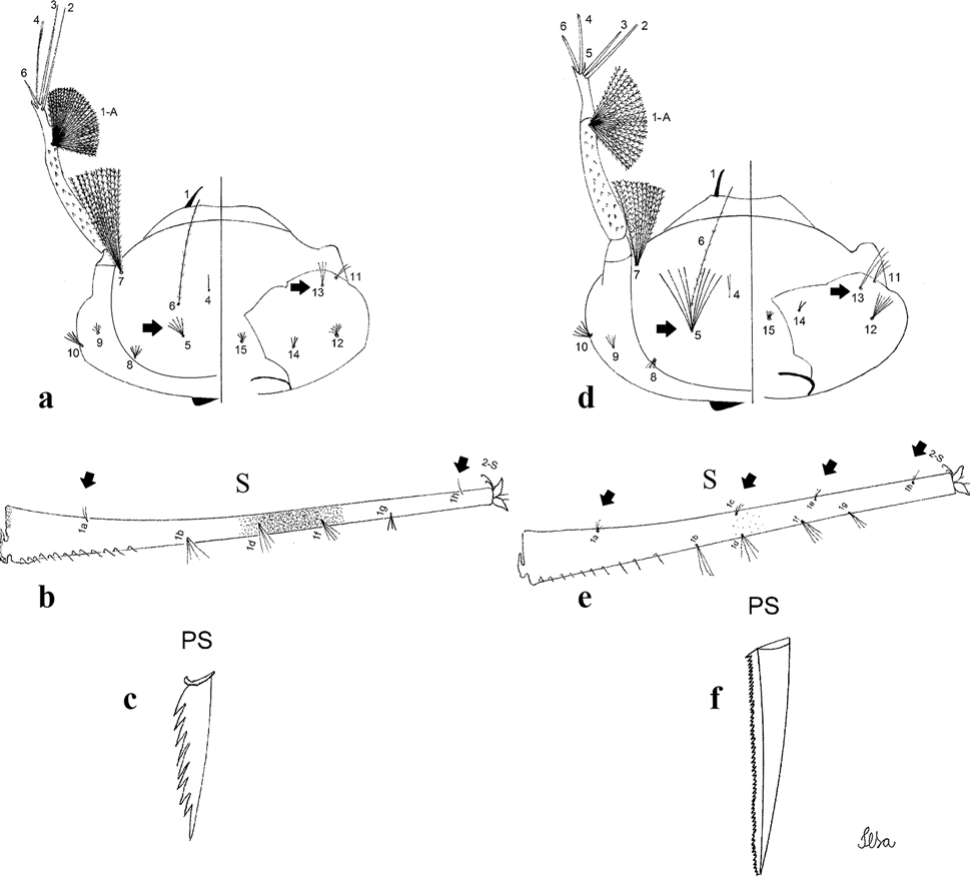
Couplet 2: head features, siphon and siphonal pecten spines. Arrows indicate head setae, siphon setae and siphonal pecten spines. **a**-**c** Thesis: *Culex trigeminatus*, setae 5 and 13 of the head (**a**), siphonal setae (**b**) and siphonal pecten spine (**c**). **d-f** Antithesis: *Culex ensiformis*, setae 5 and 13 of the head (**d**), siphonal setae (**e**) and siphonal pecten spine (**f**). *Abbreviations*: S, siphon; PS, siphonal pecten spine
5. Segment VIII with comb scales in 3 rows (Fig. 48a); seta 14-C with hyaline branches (Fig. 48b)…4
6. Segment VIII with comb scales in 4 rows (Fig. 48c); seta 14-C with 2 pigmented branches (Fig. 48d)…5

**Fig. 48:**
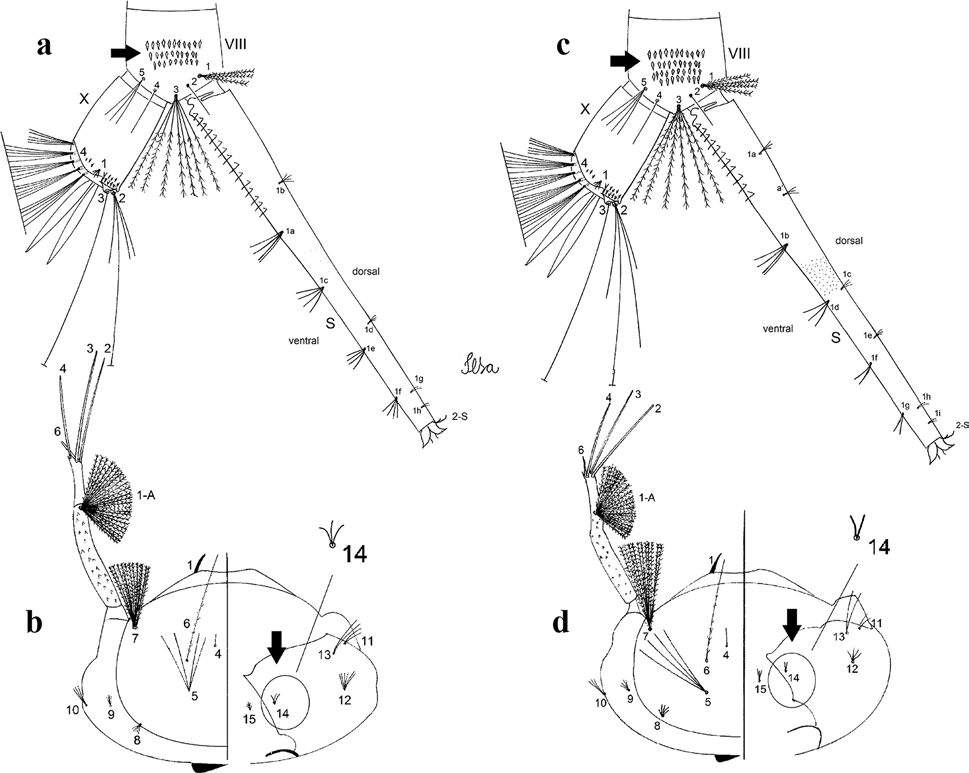
Couplet 3: abdominal segments VIII and X, siphon and head. Arrows indicate comb scales of segment VIII and head setae. **a**, **b** Thesis. **c**, **d** Antithesis. *Abbreviations*: VIII, abdominal segment VIII; X, abdominal segment X; S, siphon
7. Seta 13-C simple (Fig. 49a); pecten spines (PS) with large coarse marginal denticles (Fig. 49b)…*Cx*. *atratus*
8. Seta 13-C double (Fig. 49c); pecten spines (PS) narrow, with smaller, finer marginal spicules (Fig. 49d)…*Cx*. *comptus* n. sp.

**Fig. 49:**
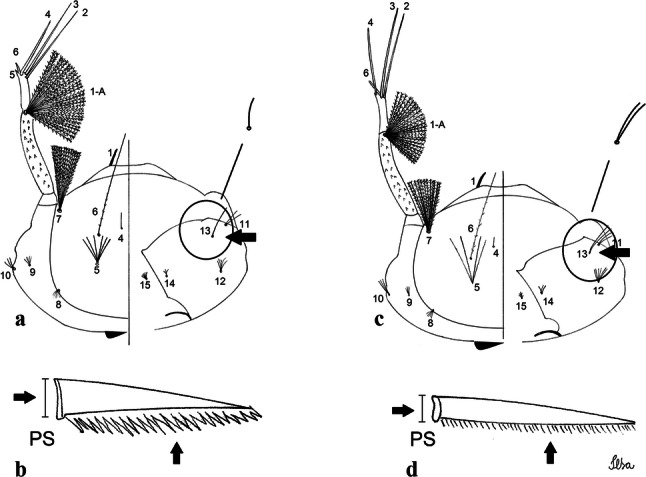
Couplet 4: head features and siphonal pecten spines. Arrows indicate head setae and siphonal pecten spine. **a**, **b** Thesis: *Culex atratus*, seta 13 of the head (**a**) and siphonal pecten spine (**b**). **c**, **d** Antithesis: *Culex comptus* n. sp., seta 13 of the head (**c**) and siphonal pecten spine (**d**). *Abbreviation*: PS, siphonal pecten spines
9. Seta 2-VIII with 2 or 3 branches; seta 5-VIII with 3 branches (Fig. 50a); comb scales (CS) with lateral and basal spicules; pecten spines (PS) short, broad basally and gradually narrowed to apex (Fig. 50b)…*Cx*. *zeteki*
10. Seta 2-VIII with 1 or 2 branches; seta 5-VIII with 3 or 4 branches (Fig. 50c); comb scales (CS) without lateral spicules; pecten spines (PS) long and narrower at base (Fig. 50d)…*Cx*. *dunni*

**Fig. 50:**
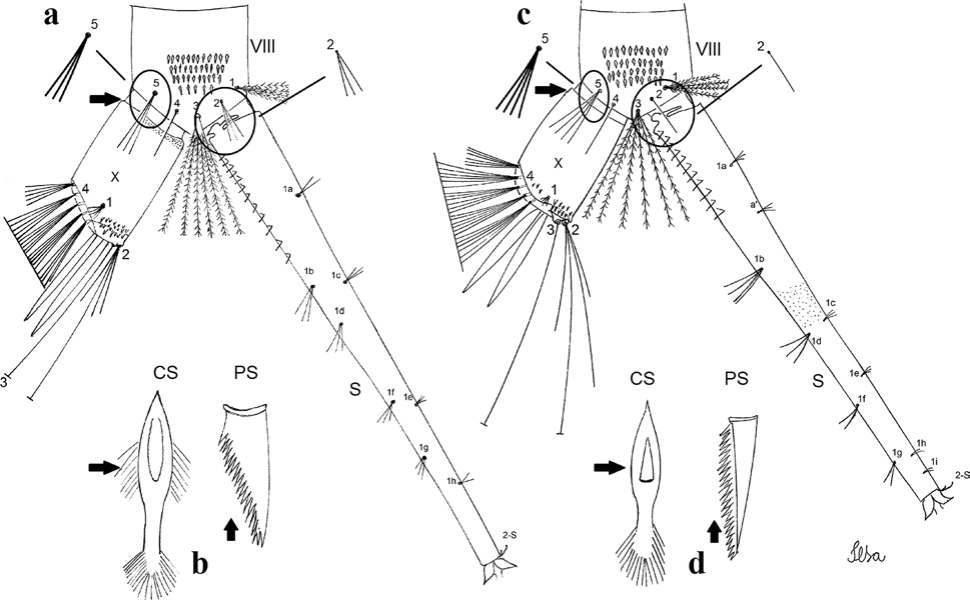
Couplet 5: abdomical segments VIII and X, siphon, comb scales and siphonal pecten spines. **a**, **b** Thesis: *Culex zeteki*, setae 2 and 5 of abdominal segment VIII (**a**) and spicules of comb scales and siphonal pecten spines (**b**). **c, d** Antithesis: *Culex dunni*, setae 2 and 5 of abdominal segment VIII (**c**) and spicules of comb scales and siphonal pecten spines (**d**). *Abbreviations*: VIII, abdominal segment VIII; X, abdominal segment X; S, siphon; CS, comb scales; PS, siphonal pecten spines

## Discussion

The Atratus Group proposed by Sirivanakarn [8] included seven valid species, namely *Cx*. *atratus*, *Cx*. *caribeanus*, *Cx*. *commevynensis*, *Cx*. *dunni*, *Cx*. *ensiformis*, *Cx*. *trigeminatus*, and *Cx*. *zeteki*. Adults of the group can be readily identified by having the vertex with narrow, decumbent scales restricted to the central area, a pleural integument with a striking pattern of dark and pale areas, and a patch of white scales on upper mesokatepisternum. In the male genitalia, the aedeagal sclerite is slender and curved in lateral view, the gonocoxite is oblong and narrow, and the gonostylus is narrow, simple and tapering to the apex. The trumpet of pupae is thin, long with a length/width ratio of 10 or higher. The fourth-instar larvae have a long, slender and tapering to apex siphon, with 3 or 4 pairs of small dorsolateral setae 1-S.

Thus, according to Sirivanakarn [8], immatures of species of the Atratus Group should have a trumpet index greater than 7.0 in pupae and possess 3 or 4 dorsolateral seta 1-S in larvae. Based on the data we have in hand, contrasting with Sirivanakarn, the pupal trumpet index of *Cx*. *atratus* is 7.0 and *Cx*. *trigeminatus* larvae possess only 2 pairs of dorsolateral setae on the siphon. These characteristics disagree with the diagnosis for the group provided by Sirivanakarn [8] but they do not invalidate the Atratus Group; they facilitate the differentiation of *Cx*. *atratus* and *Cx*. *trigeminatus* from the other species of the Group, being diagnostic of these two species.

In addition, there are several characters that are useful for identification of species of the Atratus Group. Our comparative observations of adults of all examined species clearly indicate that the presence of a ring of white scales on all of the femoro-tibial joints, represents a character that helps recognize species of the group. However, other species of the Melanoconion Section also possess white scales on the knees, such as *Cx*. *theobaldi* (Lutz).

*Culex exedrus* can be distinguished from *Cx*. *dunni* by having several long setae visibly lined up on the sternomesal surface of the gonocoxite, seta *s* of gonocoxite with a large apex, and a proctiger with a large inner process of tergum X. Therefore, these features justify the resurrection of *Cx*. *exedrus* from synonymy with *Cx*. *dunni*. Similarly, *Cx*. *loturus* was resurrected from synonymy with *Cx*. *zeteki* based on the possession of a large seta *l* borne subapically on the distal division of the subapical lobe and a slender apical process without ripples of the lateral plate of the aedeagus.

Furthermore, examination of the available material resulted in the discovery of five new species of the Atratus Group, based on morphological characters of adults, male genitalia and, where possible, of immature stages.

## Conclusions

The Atratus Group of *Culex* (*Melanoconion*) currently comprises 14 species and it has been markedly updated with respect to the number of known species, their bionomics and distribution, providing tools to facilitate the identification of the adult and immature stages of the species in the group. As a result, the current knowledge leads us to suggest the following composition of the Atratus Group: *Cx. atratus* (syns *Cx*. *advieri* and *Cx*. *falsificator*), *Cx. caribeanus*, *Cx. commevynensis*, *Cx. columnaris* n. sp., *Cx. comptus* n. sp., *Cx. dunni* (syn. *Cx*. *ruffinis*), *Cx. ensiformis*, *Cx. exedrus*, *Cx. longisetosus* n. sp., *Cx. longistylus* n. sp., *Culex loturus*, *Culex spinifer* n. sp., *Culex trigeminatus* and *Culex zeteki*. Additional studies utilizing molecular methods, particularly to investigate phylogenetic relationships within the Atratus Group, and to determine the placement of the group within the genus *Melanoconion*, are necessary.

## Data Availability

Specimens used in the present study are deposited and available in Coleção Entomológica de Referência, Faculdade de Saúde Pública, Universidade de São Paulo (FSP-USP), São Paulo State, Brazil. Specimens collected by RSGH and RWH are deposited in the Coleção de Invertebrados, Instituto Nacional de Pesquisas da Amazônia (INPA), Manaus, Amazonas State, Brazil. Holotypes and paratypes are deposited in FSP-USP under accession numbers: FSP-USP nos E-15881–E-15906. Holotypes and paratypes are deposited in INPA under accession numbers: INPA-DIP 004565–INPA-DIP 004582.
